# Genome and population sequencing of a chromosome-level genome assembly of the Chinese tapertail anchovy (*Coilia nasus*) provides novel insights into migratory adaptation

**DOI:** 10.1093/gigascience/giz157

**Published:** 2020-01-02

**Authors:** Gangchun Xu, Chao Bian, Zhijuan Nie, Jia Li, Yuyu Wang, Dongpo Xu, Xinxin You, Hongbo Liu, Jiancao Gao, Hongxia Li, Kai Liu, Jian Yang, Quanjie Li, Nailin Shao, Yanbing Zhuang, Dian Fang, Tao Jiang, Yunyun Lv, Yu Huang, Ruobo Gu, Junmin Xu, Wei Ge, Qiong Shi, Pao Xu

**Affiliations:** 1 Wuxi Fisheries College, Nanjing Agricultural University, Binhu District, Wuxi 214081, China; 2 Key Laboratory of Freshwater Fisheries and Germplasm Resources Utilization, Ministry of Agriculture, Freshwater Fisheries Research Center, Chinese Academy of Fishery Sciences, Binhu District, Wuxi, 214081, China; 3 Shenzhen Key Lab of Marine Genomics, Guangdong Provincial Key Lab of Molecular Breeding in Marine Economic Animals, BGI Academy of Marine Sciences, BGI Marine, BGI, Yantian District, Shenzhen 518083, China; 4 Centre of Reproduction, Development and Aging, Faculty of Health Sciences, University of Macau, Taipa, Macau, China; 5 BGI Education Center, University of Chinese Academy of Sciences, Yantian District, Shenzhen 518083, China; 6 Department of Biological Sciences, The George Washington University, Washington, DC 20052, USA

**Keywords:** Chinese tapertail anchovy (*Coilia nasus*), genome and population sequencing, genome assembly, migratory dimorphism and adaptation

## Abstract

**Background:**

Seasonal migration is one of the most spectacular events in nature; however, the molecular mechanisms related to this phenomenon have not been investigated in detail. The Chinese tapertail, or Japanese grenadier anchovy, *Coilia nasus*, is a valuable migratory fish of high economic importance and special migratory dimorphism (with certain individuals as non-migratory residents).

**Results:**

In this study, an 870.0-Mb high-quality genome was assembled by the combination of Illumina and Pacific Biosciences sequencing. Approximately 812.1 Mb of scaffolds were linked to 24 chromosomes using a high-density genetic map from a family of 104 full siblings and their parents. In addition, population sequencing of 96 representative individuals from diverse areas along the putative migration path identified 150 candidate genes, which are mainly enriched in 3 Ca^2+^-related pathways. Based on integrative genomic and transcriptomic analyses, we determined that the 3 Ca^2+^-related pathways are critical for promotion of migratory adaption. A large number of molecular markers were also identified, which distinguished migratory individuals and non-migratory freshwater residents.

**Conclusions:**

We assembled a chromosome-level genome for the Chinese tapertail anchovy. The genome provided a valuable genetic resource for understanding of migratory adaption and population genetics and will benefit the aquaculture and management of this economically important fish.

## Introduction

Migration is one of the most spectacular events in nature. Every year, billions of animals take part in a seasonal movement to find food or mates, avoid predators, or escape from a severe living environment. Hence, seasonal migration can influence the distribution of animals across space and time. Determining related mechanisms of migratory adaptation is critical for understanding of evolutionary processes and for facilitating management of stocks and conservation of endangered species. Many studies have aimed to understand this interesting phenomenon [1, 2]; however, the detailed molecular mechanisms are still largely unknown.

**Figure 1:**
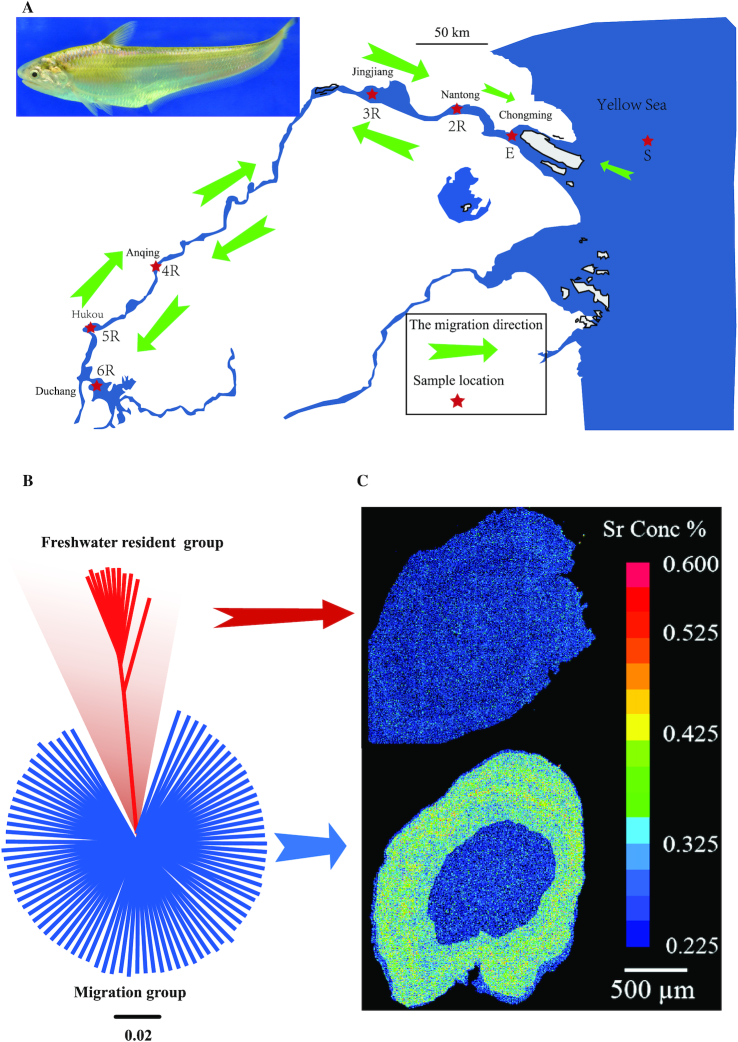
Seasonal migration and migratory dimorphism of the Chinese tapertail anchovy. (**A**) A representative image of this economically important fish and geographic distribution of the collected samples along the putative migration route. The red stars represent the sample collection sites (see details in Table   1) and the green arrows indicate the direction of reproductive migration. (B) Neighbor-joining phylogenetic tree constructed with genome-wide SNPs. The scale bar represents the similarity level. (C) Representative X-ray intensity maps of the Sr content in the otoliths of *C. nasus*. The constant blue color represents the freshwater residential pattern, while the alternative blue and green colors indicate the migratory pattern.

The Chinese tapertail anchovy, or Japanese grenadier anchovy, *Coilia nasus* (NCBI:txid365059; Fishbase ID:680; Fig. 1A), is a commercially valuable migratory fish with high economic importance in China and can be classified into 2 groups according to their living habitats. One is the routine migratory group, with a wide distribution in marine areas close to the coasts of Korea, China, and Japan. In China, this species is mainly fished from the Yellow Sea, East China Sea, and Yangtze River [3]. Similar to Pacific salmon (*Oncorhynchus* spp.) [2], *C. nasus* adults are known to migrate from February to April each year anadromously to the Yangtze River before their final gonadal maturation in order to spawn in the middle and lower reaches of the Yangtze River (details in Fig. 1A). This represents a distance of thousands of kilometers between the open ocean (for growth) and the natal stream (for reproduction) [4]. After spawning, adult fish migrate to the sea. The juveniles remain in fresh water for 3–4 months until they acquire the ability to tolerate sea water; they then follow the path of their parents and migrate to the sea [5]. The other group has been reported to be resident in some freshwater lakes during their entire lifetime [6]. This phenomenon, known as partial migration or migratory dimorphism [1], provides an opportunity to obtain insights into migratory adaptation.

Many studies have investigated this process, but most have simply described the patterns of migratory dimorphism [7] or its occurrence in a given population [8]. These data provided limited information on the related genetic variations from the perspective of the whole genome. In addition, the detailed mechanisms related to migratory dimorphism in fish are disputed and poorly understood. Thus, in this study, we first produced the chromosome-level genome assembly of *C. nasus*, based on the genetic linkage map constructed with the digest restriction site–associated DNA (RAD) sequencing [9]. After population genome sequencing of 96 individuals from diverse areas along the putative migration path (Fig. 1A and Table   1), we identified numerous single-nucleotide polymorphisms (SNPs) to detect molecular clues for adaptive variations between the migratory and freshwater resident groups. The identified candidate genes for migratory adaptation will provide valuable resources for genetic research on fish migration.

**Table 1:** Summary of sample information for the genome resequencing

Type	Locality	Sample	No.	Position
Sea	Yellow Sea	S	15	31.500 N, 122.400 E
River	Chongming	E	15	31.767 N, 121.117 E
	Nantong	2R	15	31.967 N, 120.817 E
	Jingjiang	3R	11	31.933 N, 120.233 E
	Anqing	4R	13	30.500 N, 117.067 E
Lake	Hukou	5R	13	29.733 N, 116.200 E
	Duchang	6R	14	29.233 N, 116.183 E

**Table 2:** Statistics of the genome assembly of *C. nasus*.

Genome assembly	Parameter
Contig N50 (Mb)	1.6
Contig number (>100 bp)	1,327
Scaffold N50 (Mb)	2.1
Scaffold number (>100 bp)	727
Total length (Mb)	870.0
Genome coverage (×)	404.4
Longest scaffold (Mb)	12.0
**Genome annotation**	
Protein-coding gene number	20,837
Mean transcript length (bp)	16,775.5
Mean exons per gene	10.1
Mean exon length (bp)	1,759.7
Mean intron length (bp)	1,476.0

## Results

### Sequencing, assembly, and annotation of the chromosome-level genome

We sequenced ∼277.9 Gb of short reads (100–150 bp) using the Illumina HiSeq 2500 platform (Illumina, San Diego, CA, USA) and 68.6 Gb of long reads (a mean of 14,743 bp) from the Pacific Biosciences (PacBio) RSII platform (Pacific Biosciences, Menlo Park, CA, USA) (see details in Supplementary Table 1). After removal of low-quality raw reads, we assembled a high-quality genome using the combination of Platanus (version 1.2.1, RRID:SCR_015531) and DBG2OLC results with a scaffold N50 and a contig N50 of 2.1 and 1.6 Mb, respectively (Table 2). Our genome assembly spanned ∼870.0 Mb, which is consistent with the predicted genome size of 857.5 Mb based on a *k*-mer analysis (Supplementary Fig. 1 and Supplementary Table 2) [10]. The BUSCO (University of Geneva Medical School and Swiss Institute of Bioinformatics, Geneva, Switzerland; version 3.03, RRID:SCR_015008) [11] with actinopterygii_odb9 orthologues was used to evaluate the completeness of our assembly. The assessment result of our assembly was 90.1%, where C = 87.1% [D = 4.6%], F = 3.0%, M = 9.9%, and n = 4584 (C: complete [D: duplicated], F: fragmented, M: missed, n: number of genes), thereby suggesting a high level of completeness for the *C. nasus* assembly.

**Figure 2:**
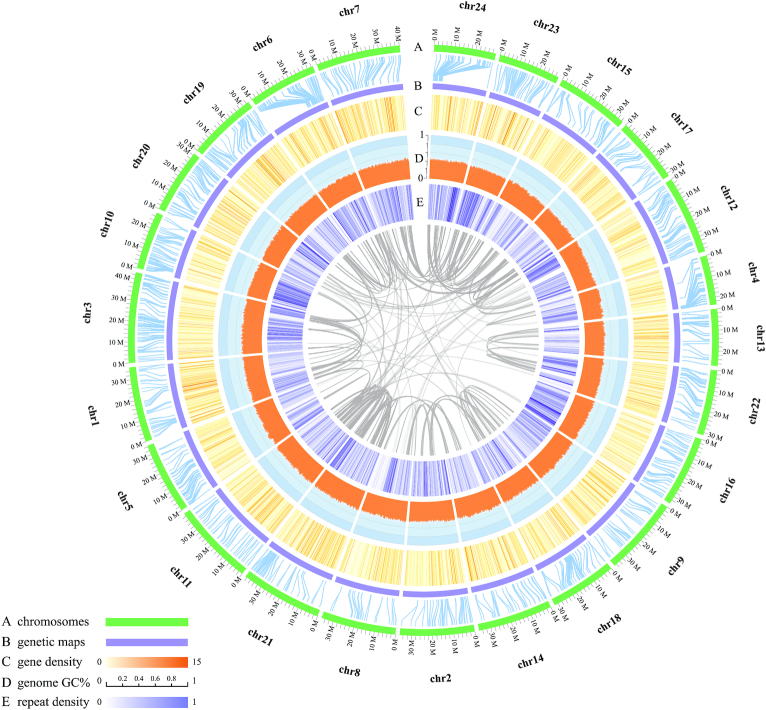
Circos plot of the genome assembly. The rings from outside to inside indicate (**A**) pseudo-chromosomes, (**B**) a genetic map, (**C**) a heat map of gene density (in orange) in 100 kb of non-overlapping windows, (**D**) line chart of the genome GC content in 100 kb of non-overlapping windows, and (**E**) a heat map of repeat density (in violet) in 100 kb of non-overlapping windows. Syntenic blocks are connected with navy lines, and each line indicates 1 paralog gene pair in the assembled genome.

In addition, a high-density linkage map of *C. nasus* based on the RAD sequencing of a family of 104 full siblings with their parent pairs was constructed. Subsequently, we localized a total of 15,300 high-quality SNPs into 24 linkage groups with a genetic distance up to 7,651.0 cM. Finally, 93.3% of the assembled genome sequences (812.1 Mb/870.0 Mb) were allocated to the 24 putative pairs of chromosomes (Fig. 2, Supplementary Fig. 2).

Repeat sequences were predicted to comprise ∼31.1% of the *C. nasus* genome (Supplementary Table 3). These repeat sequences were classified into several representative types, and it was observed that Simple and hAT repeat sequences were the most abundant types (accounting for 5.75% and 5.65%, respectively) in the genome assembly (Supplementary Table 3). We also annotated 20,837 genes with an average length of 16.8 kb (Supplementary Table 4), of which 20,300 genes have functional assignments with public databases (Supplementary Table 5). Details of the chromosomal map and markers (Supplementary Table 6), density of genes, GC content, and repeat sequences are summarized in Fig. 2.

### Population genome sequencing and identification of variations

Whole-genome population sequencing generated ∼4.5 billion 125-bp paired-end reads (i.e., 684.0 Gb of raw data). The mapping ratio (aligned reads/original reads) for each sample ranged from 64.0% to 71.0%, and the average mapping depth was determined to be ∼10 folds (Supplementary Table 8). In total, 39.4 million (M) high-confidence SNPs were called, and they were then annotated on the basis of their positions in the chromosomes. Most of the SNPs (25.3 M [64.1%]) were identified in intergenic regions, while 1.31 M of the SNPs (33.3%) were distributed in intron regions, and only 1.0 M of the SNPs (2.6%) were distributed in coding regions. Among the SNPs within coding regions, we identified 472,322 synonymous SNPs and 545,212 non-synonymous SNPs (Supplementary Table 9).

To identify the detailed divergence at the genome level among the 96 examined individuals, we constructed a phylogenetic tree based on the entire SNP set. Interestingly, the tree demonstrated that these individuals could be clearly divided into 2 groups, in which 11 were freshwater residents and 85 were migratory individuals (Fig. 1B). For confirmation of this grouping, we also used electron probe microanalysis [12] to check whether these fish were migratory. As we reported previously, the migratory group can be discriminated on the basis of the Sr (strontium) and Ca (calcium) signatures in otoliths [12–14]. Because different environmental conditions can lead to variations in the Sr contents and Sr: Ca ratios in otoliths, we used blue (Sr: Ca ratio ≤3.0), green or yellow (Sr: Ca ratio = 3.0–7.0), and red (Sr: Ca ratio > 7.0) regions in Fig. 1C and Supplementary Fig. 3 to represent freshwater, brackish water, and seawater patterns, respectively [3, 15]. Our SNP set seems to be able to clearly distinguish the divergence between these freshwater residents and migratory individuals (Fig. 1B), which was validated by the electron probe microanalysis (Fig.   1C). Therefore, this SNP set (detailed in Supplementary Table 9) can be used as genetic markers for a complement of the common performance of otolith microstructures.

### Identification of 150 candidate genes related to migratory adaption

**Figure 3:**
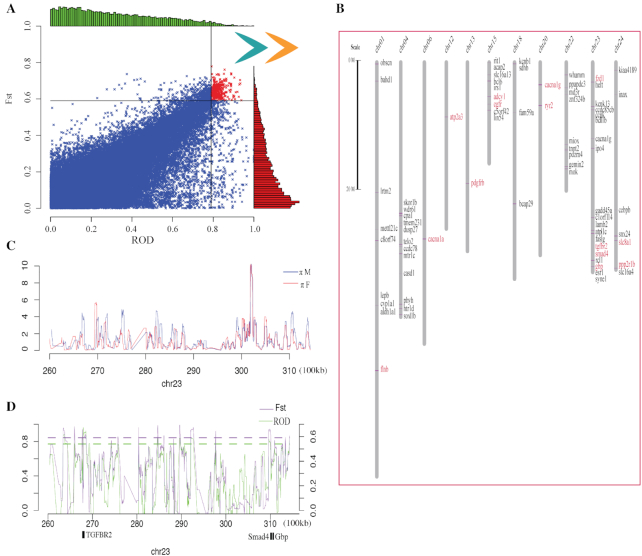
Comparison of selection sweep regions in the freshwater residential and migratory groups. (**A**) Distributions of ROD and Fst values in 5-kb non-overlapping windows. Red dots denote windows with the top 5% ROD and Fst values. (**B**) Migratory adaptation–related genes distributed on 11 chromosomes. Examples of genes (**C, D**) with selection sweep signals identified by π_migration_/π_freshwater_, Fst, and ROD values using a 5-kb sliding window. Blue and red lines represent π_migration_ and π_freshwater_, respectively. Dashed lines denote the threshold of top 5%.

We screened 661 windows with the top 5% Fst (fixation index for diversity differentiation) and ROD (reduction of diversity) values, where 150 functional genes were identified (Fig. 3A, Supplementary Table 10). These genes had potentially undergone independent selection for involvement in migratory adaptation. Interestingly, some of the selected genes were physically clustered in the assembled genome. For example, among the 150 migratory adaptation–related genes, 90 (60.0%) were distributed on 6 chromosomes (Fig. 3B, Supplementary Table 10). In particular, chromosomes 23, 4, and 15 were the 3 main chromosomes related to migratory adaptation, and 19 genes were localized on chromosome 23 (Fig. 3B). Moreover, genes with selective sweep signals were identified on the basis of π_migration_/π_freshwater_ (Fig. 3C), Fst, and ROD (Fig. 3D) using a 5-kb sliding window between the 26th and 31st Mb. Three migration-related genes, including *Tgfbr2, Smad4*, and *Gbp*, were localized within this region (Fig. 3D).

**Figure 4:**
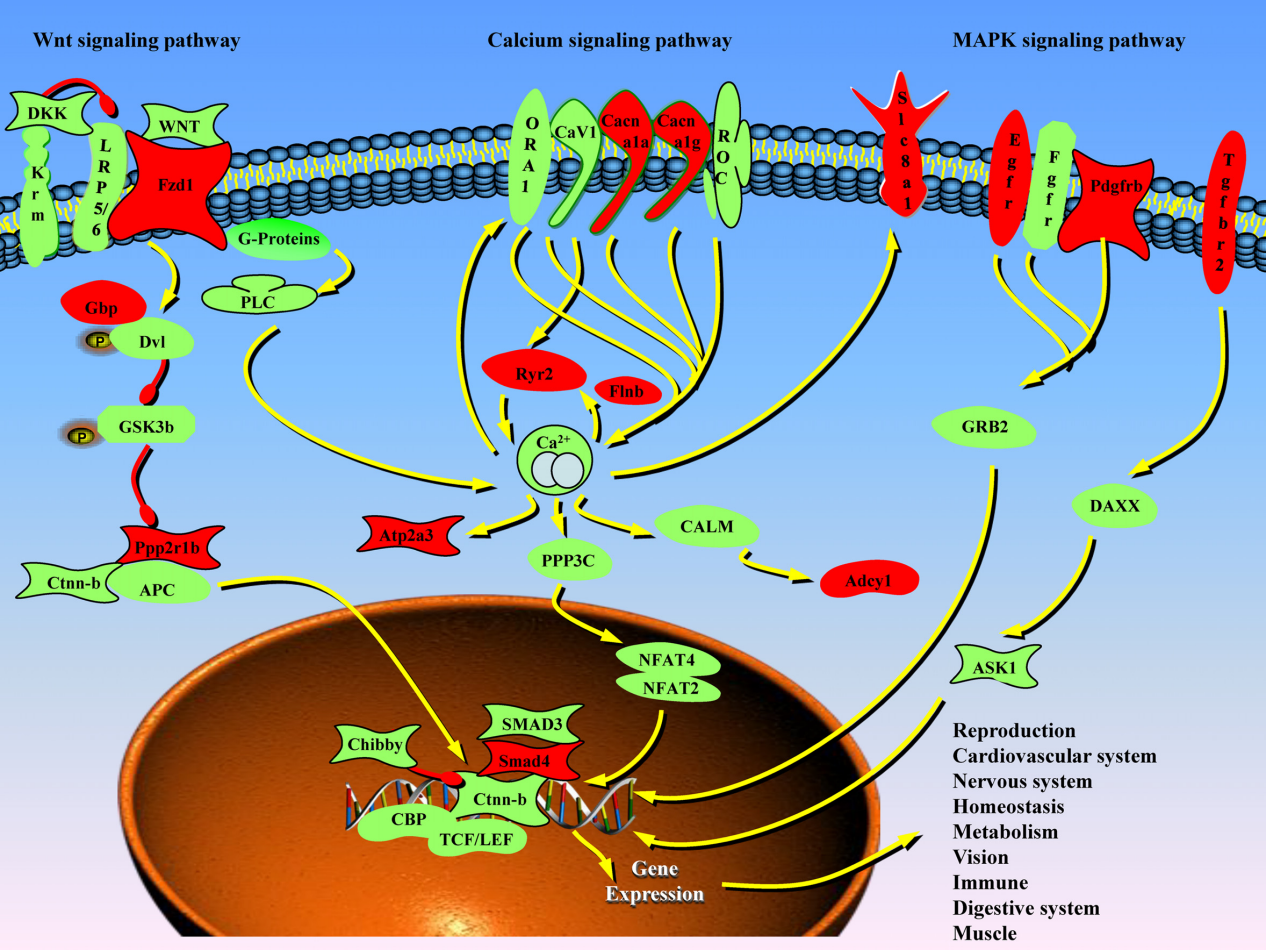
Three enriched Ca^2+^-related pathways. The genes highlighted in red were positively selected for the migratory adaptation. Green lines and arrows indicate positive regulation, and red lines, negative regulation. Interestingly, 14 of the selected genes (highlighted in red) potentially participate in the 3 critical Ca^2+^-related pathways, including calcium signaling pathway, MAKP signaling pathway, and Wnt signaling pathway.

To further clarify the functions of these 150 genes, we performed gene ontology (GO) and pathway enrichment. These genes were predicted to participate in several important functions, such as “substrate-specific transporter activity” (GO:0022892), “ion transmembrane transporter activity” (GO:0015075), “cation channel activity” (GO:0005261), “potassium channel activity” (GO:0005267), and “neuropeptide hormone activity” (GO:0005184) (Supplementary Table 11). They were significantly enriched in 11 pathways (Supplementary Table 12), which suggested that these gene terms could be related to migratory adaptation. Three pathways related to Ca^2+^ metabolism were enriched, including the calcium signaling pathway, MAPK signaling pathway, and Wnt signaling pathway (Fig. 4). These data indicate that Ca^2+^-related pathways may play key roles in adaptation to migration.

### Differentially expressed genes in the Ca^2+^-related pathways in the migratory group

**Figure 5:**
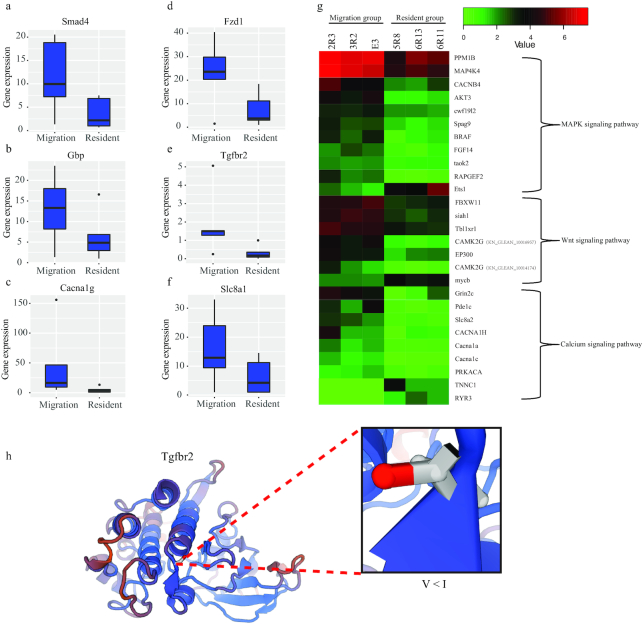
Representative mRNA transcription and protein structural changes in the selected genes with the 3 Ca^2+^-related pathways. (A-F) Quantitative RT-PCR validation of the mRNA transcription differences in 6 representative genes. (G) A heat map of the DEGs in the 3 Ca^2+^-related pathways based on the brain transcriptome. (I) Changes in the tertiary protein structure of Tgfr2.

We analyzed the variable sites in 14 genes within the 3 Ca^2+^-related pathways (red in Fig. 4). In total, 45 non-synonymous SNPs were distributed in the coding sequence regions, and their allele frequencies were significantly different (*P*-value < 0.01, Fisher exact test) in the 2 fish groups (Supplementary Table 13). To validate whether the DNA variations affected gene transcription, we quantified the messenger RNA (mRNA) changes for several randomly selected genes (Fig. 5A–F) by quantitative real-time PCR (RT-PCR). Our results demonstrated that *Smad4* (Fig. 5A), *Gbp* (Fig. 5B), *Fzd1* (Fig. 5D), *Tgfbr2* (Fig. 3B, C, and 5E), and *Slc8a1* (Fig. 5F) were transcribed more in the liver of the migratory group than that of freshwater residents (fold > 2, *P*-value < 0.05, *t*-test). Moreover, *Cacna1g*had a higher transcription level (fold > 2, *P*-value < 0.05, *t*-test; Fig. 5C) in the heart of the migratory group than the freshwater residents. We also compared the transcription values in brain tissues of the migratory and resident groups, and 648 differentially expressed genes (DEGs) were identified (*P*-value < 0.05 and fold > 2). The detailed DEG IDs and their transcription values were provided in Supplementary Table 14. In particular, 27 genes were from the 3 Ca^2+^-related pathways, and most of the genes (23) had higher transcription values in the migratory group than the freshwater residents (Fig. 5G). It seems that the migratory group maintained gene transcription of the 3 Ca^2+^-related pathways at a high level for migratory adaptation. In addition, the DNA variations may have caused changes in the tertiary structure of proteins to allow variable protein functions. For example, the 298 V site in Tgfbr2 located in the protein kinase domain (Fig. 5H) catalyzes transfer of the γ phosphate from nucleotide triphosphates to 1 or more amino acid residues in a protein substrate side chain, resulting in a conformational change to potentially affect the corresponding protein function [16, 17].

## Discussion

Fish migration is an interesting natural phenomenon. The migratory adaptation mechanisms in fish have been studied from various perspectives, such as ecology, physiology, genetics, and morphology [1, 2]. However, they have rarely been examined from a whole-genome view. After analyzing the genome sequencing and population genome sequencing data, we identified 150 candidate genes embedded in the selected sweep regions that are potentially involved in migratory adaptation. It seems that the molecular mechanisms of migratory adaptation can be interpreted at the following 3 major levels: reproductive adaptation, long-distance migratory adaptation, and complex environmental adaption.

### Genetic basis of reproductive adaptation

The main aim of migration is to spawn to ensure a wide distribution of species. Thus, migratory adaptation should first involve endocrine and reproductive adaptation. In our previous study [18], we reported that unsaturated fatty acid metabolism and steroid hormone biosynthesis are involved in the regulation of ovarian development in *C. nasus*. Of the 150 candidate genes identified in the present study, *Acoxl* is known to play an important role in the biosynthesis of unsaturated fatty acids (Supplementary Tables 10 and 11). In addition, 4 genes from oocyte meiosis and maturation pathways were included in this list of 150 genes (Supplementary Tables 10 and 11), which were also potentially involved in reproductive adaptation. Several genes with selective sweep signals in the migratory group, including *Fzd1, Ppp2r1b, Cacna1a*, and *Smad4*, were also confirmed to affect the reproductive capacity of females and males in previous knockout experiments [19–24]. Hence, they are expected to play important roles in reproductive adaptation in our migratory group.

### Positive selection of candidate genes for long-distance migratory adaptation

The *C. nasus* migratory group must undergo long-term countercurrent migration, which requires high athletic capacity. Some selective sweeping regions in the migratory group covered several important genes, such as *Atp2a3, Flnb*, and *Acna1g*, that are associated with cardiovascular, hematopoietic, and muscle functions [25–27]; these genes could participate in adaptation to long-distance migration. Moreover, genes related to nervous system development and spatial recognition, such as *Egfr, Adcy1, Flnb, Acna1g*, and *Tgfbr2*, also harbored selective sweep signals, suggesting that evolution of these genes could be important for the orientation recognition of open water in the migratory group [28, 29]. In addition, fish rarely feed during migration [30, 31]. Several digestion- and metabolism-related genes (including *Tgfbr2, Smad4, Ryr2, Cacna1a, Pdgfrb*, and *Slc8a1*) have undergone selective sweeping, which may have contributed to the highly efficient digestion and metabolism in the migratory group [24].

### Genetic adaptation to complex environments during migration

Salinity and osmotic pressure adaptations are essential for migration. It has been reported that the Ca^2+^ signaling pathways are important for regulation of osmotic pressure [32]. The critical 14 genes (red in Fig. 4) in the list of 150 DEGs with selective sweep signals were significantly enriched in the 3 central Ca^2+^-related pathways (*P* < 0.01; Supplementary Tables 12 and 13). We also observed that DNA-level variations elevated the transcription of genes in these 3 pathways to affect their functions (Fig. ). These 3 central Ca ^2+^-related pathways play a key role in cell proliferation and osmotic pressure regulation [33–35]. We also found that 6 genes with strong selective sweep signals were significantly enriched in GO terms of metal and calcium ion transport (*P* < 0.05; Supplementary Table 11), which could also be related to salinity and osmotic pressure adaptation.

In addition, some genes (such as *Flnb, Tgfbr2, Pdgfrb*, and *Smad4*; Supplementary Table 13) related to renal function and homeostasis also underwent selective sweeping, suggesting their potential contribution to the alternative adaptation to salt water and fresh water [36]. Previous studies showed that the visual and olfactory systems were essential for migratory fish [37, 38]. Interestingly, some visual and olfactory-related genes were also identified among the 150 candidate genes in the migratory group of *C. nasus* (Supplementary Table 11).

## Conclusions

In summary, we performed whole-genome sequencing of the Chinese tapertail anchovy (*C. nasus*) and constructed a high-density genetic linkage map to generate a high-quality chromosomal map. In total, 96 individuals were collected over a range of 618 km during reproductive migration for population genome sequencing. On the basis of these data and otolith X-ray electron microprobe validation, we determined 11 individuals as freshwater residents whereas the remaining individuals were migratory fish. Our high-quality reference genome and population genome sequencing data provide a good opportunity to examine the migration process and reveal a more comprehensive image of *C. nasus* population genetics that will facilitate practical aquaculture and management of this economically important fish. Identification of 150 candidate genes with significant enrichment in 3 critical Ca^2+^-related pathways potentially supports the molecular mechanisms of migratory adaptation at the following 3 major levels: reproductive adaptation, long-distance migratory adaptation, and complex environmental adaptation.

## Materials and Methods

### Sample collection and sequencing

A healthy female *C. nasus*, cultivated at our local base in Yixing city (Jiangsu Province, China) with a body weight of 167.0 g, was used for whole-genome sequencing. Skeletal muscle was collected and immediately stored in liquid nitrogen. Genomic DNA (a total of ∼90 μg) was extracted using Qiagen Genomic Tip100 (Qiagen, Hilden, Germany). The traditional whole-genome shotgun sequencing strategy was used [39]. Three short-insert libraries (250, 500, and 800 bp) and 4 long-insert libraries (2, 5, 10, and 20 kb) were constructed using Illumina reagents (Illumina, San Diego, CA, USA) in accordance with the manufacturer's instructions.

AMPure PB magnetic beads (Pacific Biosciences, Menlo Park, CA, USA) were utilized to concentrate the extracted high-quality genomic DNA for library construction with the SMRTbell template prep kit 2.1 (Pacific Biosciences). Sequencing was performed on a PacBio Sequel platform.

Based on the putative migration path of *C. nasus*, 96 individuals were sampled from different localities in the Yellow Sea, Chongming, Nantong, Jingjiang, Anqing, Hukou, and Duchang (see details in Fig. 1B and Table 1). Genomic DNA (∼3 μg DNA from each individual) was isolated from skeletal muscle using Qiagen Genomic Tip100 (Qiagen). The population genome sequencing library (average insert size of ∼350 bp) of each individual was independently constructed for DNAs from the 96 individuals, and 2 × 150 bp paired-end reads were generated by an Illumina HiSeq 2500 platform.

All animal experiments in this study were performed in accordance with the guidelines of the Animal Ethics Committee and were approved by the Institutional Review Board on Bioethics and Biosafety of BGI (No. 18134).

### Estimation of genome size and assembly of the genome

The *C. nasus* genome size (G) was estimated by a *k*-mer analysis [10] according to the following formula: G = Kmer_num/Kmer_depth, where Kmer_num is the total number of reads and Kmer_depth represents the frequency of occurring more frequently than others.

SOAPdenovo2 (version 2.04.4; RRID:SCR_014986) [40] with optimized parameters (pregraph -K 27 -d 1; contig -M 1; scaff -F -b 1.5 -p 16) was used to construct contigs and original scaffolds based on the sequenced reads. Subsequently, total reads were mapped onto the contigs by the third step of SOAPdenovo with default parameters for scaffolding according to the long-insert paired-end information, which led to linkage of contigs to scaffolds in a stepwise manner. Approximately 109.2 Gb of cleaned reads from the short-insert (250, 500, and 800 bp) libraries were then used to fill gaps in scaffolds with the GapCloser (v1.12-r6; RRID:SCR_015026; default parameters and -p set at 25). Finally, the first version of the genome assembly was generated. The BUSCO value achieved was 88.6%, where C = 86.8% [D = 4.5%], F = 1.8%, M = 11.4%, and n = 3023 (C: complete [D: duplicated], F: fragmented, M: missed, n: number of genes).

To improve the *de novo* assembly, 68.6 Gb of PacBio reads were also sequenced. Platanus (version 1.2.1, RRID:SCR_015531) [41] was used to generate a *de novo* assembly with a total of 1.0 Gb and a contig N50 of 764 bp using Illumina reads from the short-insert (250, 500, and 800 bp) libraries. Subsequently, all PacBio reads and the above assembled contigs were used for further assembly by utilizing the DBG2OLC pipeline (default version) [42] with the following parameters: LD10, MinLen 200, KmerCovTh 6, MinOverlap 80, AdaptiveTh 0.012, and RemoveChimera 1. A polishing step for this assembly was then performed using Illumina reads from the short-insert libraries. These reads were mapped onto the contigs using BWA-MEM (version 0.6.2, RRID:SCR_010910) [43]. Pilon (version 1.22, RRID:SCR_014731) [44] was also used to correct the assembly according to the alignment. SSPACE (version 3.0, RRID:SCR_005056) [45] was then used to generate scaffolds with the Illumina reads from the long-insert libraries (2, 5, 10, and 20 kb). Redundans (version 0.14a) [46] with parameters (–identity 0.3 –overlap 0.3 –minLength 1000) was used to remove redundant scaffolds caused by the high heterozygosity of the *C. nasus* genome.

### Genome annotation

For repeat annotation, Repeat Modeler version 1.04 (Repeat Modeler, RRID:SCR_015027) [47] and LTR_FINDER version 1.06 (LTR_FINDER, RRID:SCR_015247) [48] were used to construct a *de novo* repeat library with default parameters. RepeatMasker version 3.2.9 (RepeatMasker, RRID:SCR_012954) [49] was then used to search the repeat sequences against Repbase TE (version 14.04) [50] and the *de novo* repeat libraries to identify known and novel transposable elements (TEs) in the *C. nasus* genome. The tandem repeats were identified by using Tandem Repeat Finder (version 4.04) [51], where the core parameters were set as “Match = 2, Mismatch = 7, Delta = 7, PM = 80, PI = 10, Minscore = 50, and MaxPerid = 2000.” Furthermore, the relevant TE proteins were screened in the *C. nasus* assembly using RepeatProteinMask (version 3.2.2) [49].

A combined annotation pipeline of 3 separate approaches, including homology, *de novo*, and transcriptome-based annotations were used to predict gene structures and functions. For the homology annotation, protein sequences from zebrafish, Japanese fugu, spotted green pufferfish, Japanese medaka, and stickleback (Ensembl release 75) were downloaded to map onto the *C. nasus* genome using BLAT (e-value ≤1E-5; version 319, RRID:SCR_011919) [52]. Genewise version 2.2.0 (Genewise, RRID:SCR_015054) [53] was then used to predict the potential gene structures based on all the alignments generated from the previous step. Short genes (<150 bp) and prematurely terminated or frame-shifted genes were discarded. For the *de novo* annotation, 1,000 complete genes were randomly chosen from the homology annotation set to train parameters for AUGUSTUS (version 3.0.2, RRID:SCR_008417) [54]. Repeat regions were masked by “N” in our genome assembly. AUGUSTUS was then used to make *de novo* predictions based on the repeat-masked genome assembly. The *de novo* annotation results were filtered using the same method for the homology prediction. For the transcriptome-based annotation, total RNA was extracted from the muscle and liver tissues from the same female fish for whole-genome sequencing. The sequencing reads were aligned onto the genome assembly using HISAT2 version 0.1.6 (HISAT2, RRID:SCR_015530) [55]. These alignments were sorted by using the samtools software (version 1.2, RRID:SCR_002105) [56]. Cufflink (version 2.2.1, RRID:SCR_014597) [57] was then used to identify potential gene structures. The results obtained by all 3 annotation methods were merged to produce a comprehensive and non-redundant gene set using Maker (version 2.31.8, RRID:SCR_005318) [58].

All the protein sequences obtained from the Maker results were mapped onto the SwissProt and TrEMBL databases [59] by BLASTP (version 2.2.25, RRID:SCR_001010) [60] with an E-value ≤ 1e−5 to find the best hit for each protein. We also used the InterProScan (version 4.7, RRID:SCR_005829) [61] to align the protein sequences against other public databases, including Pfam [62], PRINTS [63], ProDom [64], and SMART [65], in order to determine the known motifs and domains in our protein sequences. Finally, 20,300 genes proved to contain ≥1 functional assignment from public databases, including Swiss-Prot and TrEMBL [59], GO [66], and KEGG [67] (Supplementary Table 5).

### RAD sequencing and genotyping

RAD sequencing [9] was performed to generate a set of SNP markers from a full-sibling family F1 group. In brief, the procedure is described as follows.

#### DNA extraction and sequencing

Genomic DNA from the 104 offspring individuals and their parents was separately extracted from the fin clips using a Mag Attract HMW DNA Kit (Qiagen, Gaithersburg, MD, USA). PstI restriction enzyme was used for digestion of DNA, and for constructing the RAD sequencing libraries, which were subsequently sequenced on an Illumina HiSeq 2500 platform. The adaptors of raw reads and the reads with low quality were filtered with a local perl script.

#### SNP calling

BWA-MEM (parameters: aln -n 0.04 -o 1 -e 30 -i 15 -d 10 -l 35 -k 2 -m 2000000 -t 4 -M 3 -O 11 -E 4 -R 30 -q 0 -I –f, version: 0.7.12, RRID:SCR_010910) [43] was used to align cleaned reads upon the second version of genome assembly. Subsequently, GATK (version 3.1, RRID:SCR_001876) [68] was used to perform SNP calling. Related parameters for GATK were set as “QD < 2.0 || FS > 60.0 || MQ < 40.0 || MQRankSum < -12.5 || ReadPosRankSum < -8.0.”

### Construction of the genetic linkage map, chromosomal map, and identification of synteny blocks

JoinMap (version 4.1, RRID:SCR_009248) [69] with logarithm of odds values ranging from 2 to 12 was used to evaluate the map distance under the regression mapping algorithm. Subsequently, we constructed a high-density genetic linkage map with 24 linkage groups, which is consistent with the results of a previous report [70].

Based on the SNP markers and genetic linkage map, a preliminary chromosomal-level assembly was generated. Locations of the scaffolds in each chromosome were fixed according to the following rules. For the scaffolds with sufficient SNP markers (>2), we chose the 2 markers with the highest quality to determine their location and direction. However, directions of those scaffolds with insufficient SNP markers (only 1) were not fixed, but instead they were placed directly onto the chromosomes. The protein alignments were conducted by performing BLASTP with an E-value < 1e−5. Then, MCscan (version 0.8) [71] was used to identify the gene-level syteny blocks from the BLASTP alignments with the parameter setting as “-a -e 1e-5 -s 5 -u 1.”

### SNP calling and phylogenetic analysis

SOAPfilter (version 2.2), a package from SOAPdenovo2 (version 2.04.4; RRID:SCR_014986) [40], was used to filter the population sequencing reads with adaptors, low quality, undersize inserts, or PCR duplicates. The cleaned reads were then aligned onto our genome assembly (first version) using BWA-MEM (version 0.7.1, RRID:SCR_010910) [43]. SNP calling was performed using a standard GATK (version 3.1, RRID:SCR_001876) [68]. Quality filtering was realized for the raw variant calls using GATK with the following cut-offs: QD < 2.0, MQ < 40.0, FS > 60.0, MQRankSum ≤ 12.5, ReadPosRankSum ≤ 8.0, and DP < 100. The variants with >10% missing data were excluded and used a minor allele frequency filter of 10%. Then SnpEff (version 3.4, RRID:SCR_005191) [72] was used to annotate the genetic variants and categorized the variants into coding (synonymous and non-synonymous), upstream/downstream, and intronic/intergenic classes. PLINK (version 1.07, RRID:SCR_001757) [73] with parameters “–distance 1-ibs flat-missing” was used to calculate the genetic distances among individuals, which were subsequently used to generate neighbor-joining trees with fneighbor (PHYLIPNEW v3.69.650 within the package of EMBOSS v 6.6.0.0, RRID:SCR_006244) [74].

### Identification of selective sweep regions

Reduction of diversity was defined as ROD = 1 − π_freshwater_/π_migration_, where π_freshwater_ and π_migration_ are the average numbers of nucleotide differences per site [75] from the freshwater and the migratory groups, respectively. The Fst and ROD values in a sliding window of 5 kb along the genome assembly were calculated using the entire SNP set. Genomic regions located in the top right corner of Fig. 3A, corresponding to a 5% significant level of the Fst and ROD values (>0.79 and 0.59, respectively), were considered the selective sweep regions. Finally, 150 genes were identified in this region and these genes were enriched in GO terms using the Enrich Pipeline as described previously [76]. EnrichmentPipeline analysis [77] for a given gene list was carried out based on the algorithm implemented in GOstat, with the whole annotated gene set as the background. GOstat tests for GO terms that are represented by significantly more genes in a given gene set using χ^2^ test. Fisher exact test was used when expected counts were <5, which makes the χ^2^ test inaccurate.

### Transcriptome analysis of freshwater and migratory individuals and validation by quantitative RT-PCR

For transcriptome sequencing, total RNA was extracted from the brain tissues of 3 randomly selected individuals in the migratory or freshwater groups using TRIzol reagent (Invitrogen, Carlsbad, CA, USA). Using a HiSeq 4000 platform, 125-bp paired-end Illumina reads were generated for transcriptome sequencing. Raw data produced from the sequencing platform were filtered by removing reads contaminated with adaptors, >10% of N bases, and >50% of low-quality bases (base quality score ≤ 10). These cleaned RNA reads were aligned onto the reference genome (first version) using HISAT2 (version 0.1.6, RRID:SCR_015530) with parameters “–phred33 –sensitive –no-discordant –no-mixed -I 1 -X 1000” [55]. Expression values were calculated by Cufflink (version 2.2.1, RRID:SCR_014597) with defaulted parameters [57]. The Cuffdiff in the Cufflink package with parameters “-FDR 0.05 –geometric-norm TRUE –c 10” was used to identify the significant DEGs. The edgeR software (RRID:SCR_012802) [78] was used to draw the heat map view with the threshold *P*-value < 0.05 and folds > 2. Finally, the enriched GO terms were identified for these DEGs using the Enrich Pipeline as described previously [76].

For the quantitative RT-PCR, brain tissues were obtained from 5 individuals in each group, and total RNA was extracted separately with TRIzol reagent (Invitrogen). First-strand complementary DNA was subsequently synthesized using a PrimeScript™ RT reagent kit with gDNA Eraser (Takara, Kusatsu, Shiga, Japan), and 18S RNA was used as the internal control. Sequences of the primer pairs are provided in Supplementary Table 15. Transcription of the target genes was calculated as the relative increase according to the 2^–ΔΔCT^ method [79]. Normal distribution and homogeneity of variance of data was tested with the Shapiro-Wilk and Levene tests (α = 0.05), respectively. Then differences in the mRNA levels were compared by the Student *t*-test using IBM SPSS Statistics 22.0 (IBM Inc., Chicago, IL, USA). *P* values of <0.05 were considered statistically significant.

### Measurement of Sr and Ca content in otoliths

The Sr and Ca content in otoliths were measured as described in our previous report [3]. In brief, the otoliths were embedded in epoxy resin (EpoFix, Struers, Copenhagen, Denmark) for grinding and polishing to expose their cores with an automated grinding machine (Roto Pol-35, Struers, KY, USA). After cleaning in an ultrasonic bath, rinsing by deionized water, and carbon-coating with a high-vacuum evaporator (JEE-420, JEOL Ltd., Tokyo, Japan), the samples were measured using a wave-dispersive X-ray electron probe micro-analyzer (JXA-8100, JEOL Ltd., Welwyn Garden City, UK). Tausonite (SrTiO_3_) and calcite (CaCO_3_) were used as the internal standards.

## Availability of Supporting Data and Materials

Genome assemblies reported here have been deposited at the GenBank under the project ID PRJNA421870. Genome *de novo*, population genome sequencing, RAD, and transcriptome sequencing data have been deposited at the NCBI SRA under the project ID PRJNA422339. Supporting data and materials are also available in the *GigaScience* GigaDB database [80].

## Additional Files

**Supplementary Figure 1**. A *k*-mer view of the sequenced *Coilia nasus* genome.

**Supplementary Figure 2**. Construction of linkage groups (or pseudo-chromosomes) of *C. nasus* by RAD sequencing. The light green bars depict the pseudo-chromosomes, and the blue lines represent the position of SNP markers in each chromosome.

**Supplementary Figure 3**. X-ray intensity maps of Sr content in otoliths of *C. nasus*. The constant blue color represents the freshwater resident pattern, while the alternating blue and green colors indicate the migratory pattern.

**Supplementary Table 1**. Statistics of sequencing reads from Illumina HiSeq 2500 and PacBio platforms

**Supplementary Table 2**. Summary of the *k*-mer data

**Supplementary Table 3**. Detailed classifications of repeat sequences

**Supplementary Table 4**. Summary of gene annotations

**Supplementary Table 5**. Summary of function annotations

**Supplementary Table 6**. Summary of marker number, genetic distance, and physical length of each pseudochromosome

**Supplementary Table 7**. Statistics of the SOAPdenovo assembly of *C. nasus*

**Supplementary Table 8**. Summary of map ratio for the 96 resequenced samples

**Supplementary Table 9**. Location sites of the genetic variants

**Supplementary Table 10**. List of the 150 candidate genes

**Supplementary Table 11**. GO functions of the 150 candidate genes (see the Supplementary Spreadsheet 1)

**Supplementary Table 12**. Pathway enrichments of the 150 candidate genes

**Supplementary Table 13**. Summary of the 277 non-synonymous SNPs in the CDS regions of the 14 Ca^2^^+^-related genes (see the separate excel file)

**Supplementary Table 14**. Differential expression genes between the migratory group and the freshwater resident group (see the separate excel files).

**Supplementary Table 15**. Sequences of the primer pairs for quantitative RT-PCR

giz157_GIGA-D-19-00179_Original_Submission

giz157_GIGA-D-19-00179_Revision_1

giz157_GIGA-D-19-00179_Revision_2

giz157_Response_to_Reviewer_Comments_Original_Submission

giz157_Response_to_Reviewer_Comments_Revision_1

giz157_Reviewer_1_Report_Original_Submission Ole K TÃ,rresen -- 7/1/2019 Reviewed

giz157_Reviewer_1_Report_Revision_1 Ole K TÃ,rresen -- 10/21/2019 Reviewed

giz157_Reviewer_2_Report_Original_Submission Bruno Louro -- 7/8/2019 Reviewed

giz157_Reviewer_2_Report_Revision_1 Bruno Louro -- 11/2/2019 Reviewed

giz157_Supplemental_Figures_and_Tables

## Abbreviations

*Adcy1*: adenylate cyclase 1; *Acoxl*: acyl-coenzyme A oxidase-like protein; *Atp2a3*: ATPase sarcoplasmic/endoplasmic reticulum Ca2+ transporting 3; BLAST: Basic Local Alignment Search Tool; BLAT: BLAST-Like Alignment Tool; bp: base pairs; BUSCO: Benchmarking Universal Single-Copy Orthologs; BWA: Burrows-Wheeler Aligner; Ca: calcium; *Cacna1a*: calcium voltage-gated channel subunit α1 A; *Cacnalg*: voltage-dependent T-type calcium channel subunitα-1 G; DEG: differentially expressed gene; *Egfr*: epidermal growth factor receptor; Fst: fixation index for diversity differentiation; *Flnb*: filamin B; *Fzd1*: frizzled-1; GATK: Genome Analysis Tool Kit: Gb: gigabase pairs; Gbp: GSK-3-binding protein; GC: guanine-cytosine; GO: Gene Ontology; kb: kilobase pairs; KEGG: Kyoto Encyclopedia of Genes and Genomes; Mb: megabase pairs; mRNA: messenger RNA; NCBI: National Center for Biotechnology Information; PacBio: Pacific Biosciences; *Pdgfrb*: platelet-derived growth factor receptor β; Ppp2r1b: serine/threonine-protein phosphatase 2A 65 kDa regulatory subunit A β isoform; RAD: restriction-site–associated DNA; ROD: reduction of diversity; *Ryr2*: ryanodine receptor 2; *Slc8a1*: solute carrier family 8 member A1; *Smad4*: SMAD family member 4; SNP: single-nucleotide polymorphism; Sr: strontium; SRA: Sequence Read Archive; TE: transposable element; *Tgfbr2*: transforming growth factor β receptor 2.

## Competing Interests

The authors declare that they have no competing interests.

## Funding

This study was supported by grants from the National Natural Science Foundation of China (Nos. 31672643, 31372533, 31502152), the General Program of Natural Science Foundation of Jiangsu Province of China (No. BK20191145), Three New Projects of Agricultural Aquaculture Program of Jiangsu Province (No. Y2018-17), and the Special Fund of Jiangsu Province for the Transformation of Scientific and Technological Achievements (No. BA2015167).

## Authors' Contributions

P.X. conceived the study and designed the project. G.X. managed the project. K.L., D.X., Y.W., Q.L., N.S., Y.Z., and Z.J.N. prepared all samples used in this study. C.B. performed genome assembly, annotation, resequencing data analyses, and transcriptome expression calculation. J.L. constructed the genetic map and chromosomal map. Y.H. and Y.L. implemented phylogenetic analysis. H. Li and H. Liu, J.Y., J.G., D.F., and T.J. measured the Sr and Ca content of otoliths. P.X., Q.S., G.X., C.B., J.L., X.Y., R.G., W.G., and J.X. discussed the data. C.B., G.X., and J.L. wrote the manuscript. Q.S., G.X., and P.X. revised the manuscript. All authors contributed to data interpretation.
