# Supplementary material for: Genome and population sequencing of a chromosome-level genome assembly of the Chinese tapertail anchovy (Coilia nasus) provides novel insights into migratory adaptation
Source: Gigascience. 2020 Jan 2;9(1):giz157. doi: 10.1093/gigascience/giz157 (PMC6939831; doi:10.1093/gigascience/giz157)
Supplement: giz157_GIGA-D-19-00179_Original_Submission [file giz157_giga-d-19-00179_original_submission.pdf]

GigaScience

Genome sequencing and resequencing for a chromosome-level genome assembly of Chinese tapertail anchovy (Coilia nasus) to provide novel insights into migratory adaptation

--Manuscript Draft--

**Manuscript Number:**	GIGA-D-19-00179
**Full Title:**	Genome sequencing and resequencing for a chromosome-level genome assembly of Chinese tapertail anchovy (Coilia nasus) to provide novel insights into migratory adaptation
**Article Type:**	Research
**Funding Information:**	the National Natural Science Foundation of China (31672643) — Dr Gangchun xu
**Abstract:**	Background<br><br>Seasonal migration is one of the most spectacular events in nature, but detailed mechanisms related to this interesting phenomenon have not been clearly investigated. Chinese tapertail anchovy, Coilia nasus, is a valuable migratory fish with high economic importance and special migratory dimorphism (with certain individuals as non-migratory residents).<br><br>Results<br><br>In this study, we assembled an 870.0-Mb high-quality genome by combination of Illumina and PacBio sequencing for this fish, in which 812.1 Mb of scaffolds were linked to 24 chromosomes with assistance of a high-density genetic map from a family of 104 full siblings and their parents. In addition, whole-genome resequencing of 96 representative individuals from diverse areas along the putative migration path clarified involvement of 150 genes in the migratory adaption. Based on integrative genomic and transcriptomic analyses, we determined that three Ca2+-related pathways are critical for promotion of the migratory adaption. Meanwhile, we identified a large amount of molecular markers for distinguishing of migratory individuals and non-migratory freshwater residents.<br><br>Conclusions<br><br>We assembled a chromosome-level genome for the Chinese tapertail anchovy. It provides a valuable genetic resource for understanding the migratory adaption and population genetics, and will benefit for aquaculture and management of this economically important fish.
**Corresponding Author:**	Qiong Shi, PhD<br>BGI<br>Shenzhen, CHINA
**Corresponding Author Secondary Information:**	
**Corresponding Author's Institution:**	BGI
**Corresponding Author's Secondary Institution:**	
**First Author:**	Qiong Shi, PhD
**First Author Secondary Information:**	
**Order of Authors:**	Qiong Shi, PhD
	Gangchun xu
	Chao Bian

Powered by Editorial Manager® and ProduXion Manager® from Aries Systems Corporation

	Zhijuan Nie
	Yuyu Wang
	Dongpo Xu
	Xinxin You
	Hongbo Liu
	Jiancao Gao
	Changyou Song
	Kai Liu
	Jian Yang
	Quanjie Li
	Nailin Shao
	Yanbing Zhuang
	Dian Fang
	Tao Jiang
	Yunyun Lv
	Yu Huang
	Ruobo Gu
	Junmin Xu
	Wei Ge
	Pao Xu
**Order of Authors Secondary Information:**	
**Additional Information:**	
**Question**	**Response**
Are you submitting this manuscript to a special series or article collection?	No
**Experimental design and statistics**<br><br>Full details of the experimental design and statistical methods used should be given in the Methods section, as detailed in our Minimum Standards Reporting Checklist. Information essential to interpreting the data presented should be made available in the figure legends.<br><br>Have you included all the information requested in your manuscript?	Yes
**Resources**<br><br>A description of all resources used,	Yes

Powered by Editorial Manager® and ProduXion Manager® from Aries Systems Corporation

including antibodies, cell lines, animals and software tools, with enough information to allow them to be uniquely identified, should be included in the Methods section. Authors are strongly encouraged to cite Research Resource Identifiers (RRIDs) for antibodies, model organisms and tools, where possible.<br><br>Have you included the information requested as detailed in our Minimum Standards Reporting Checklist?	
**Availability of data and materials**<br><br>All datasets and code on which the conclusions of the paper rely must be either included in your submission or deposited in publicly available repositories (where available and ethically appropriate), referencing such data using a unique identifier in the references and in the "Availability of Data and Materials" section of your manuscript.<br><br>Have you have met the above requirement as detailed in our Minimum Standards Reporting Checklist?	Yes

Manuscript Click here to download Manuscript manuscript.docx

Article

Genome sequencing and resequencing for a chromosome-level genome assembly of Chinese tapertail anchovy (*Coilia nasus*) to provide novel insights into migratory adaptation

Gangchun Xu[1,2†], Chao Bian[3,4†], Zhijuan Nie[2†], Jia Li[3†], Yuyu Wang[2], Dongpo Xu[2], Xinxin You[3,5], Hongbo Liu[2], Jiancao Gao[2], Changyou Song[2], Kai Liu[2], Jian Yang[2], Quanjie Li[2], Nailin Shao[2], Yanbing Zhuang[2], Dian Fang[2], Tao Jiang[2], Yunyun Lv[3,5], Yu Huang[3,5], Ruobo Gu[2], Junmin Xu[3], Wei Ge[4], Qiong Shi[3,5,*], Pao Xu[1,2,*]

[1]Wuxi Fisheries College, Nanjing Agricultural University, Wuxi, Jiangsu 214081, China.
[2]Key Laboratory of Freshwater Fisheries and Germplasm Resources Utilization, Ministry of Agriculture, Freshwater Fisheries Research Center, Chinese Academy of Fishery Sciences, Wuxi, Jiangsu 214081, China.
[3]Shenzhen Key Lab of Marine Genomics, Guangdong Provincial Key Lab of Molecular Breeding in Marine Economic Animals, BGI Academy of Marine Sciences, BGI Marine, BGI, Shenzhen, Guangdong 518083, China.
[4]Centre of Reproduction, Development and Aging, Faculty of Health Sciences, University of Macau, Taipa, Macau, China.
[5]BGI Education center, University of Chinese Academy of Sciences, Shenzhen, Guangdong 518083, China.

[*]Correspondence address. Pao Xu, Freshwater Fisheries Research Center, Chinese Academy of Fishery Sciences, Wuxi, Jiangsu 214081, China. Tel: +86-138 0619 0669; E-mail: xup@ffrc.cn; Qiong Shi, BGI Academy of Marine Sciences, BGI Marine, BGI, Shenzhen, Guangdong 518083, China. Tel: +86-185 6627 9826; E-mail: shiqiong@genomics.cn
[†]Contributed equally to this work.

Abstract

Background: Seasonal migration is one of the most spectacular events in nature, but detailed mechanisms related to this interesting phenomenon have not been clearly investigated. Chinese tapertail anchovy, *Coilia nasus*, is a valuable migratory fish with high economic importance and special migratory dimorphism (with certain individuals as non-migratory residents). **Results:** In this study, we assembled an 870.0-Mb high-quality genome by combination of Illumina and PacBio sequencing for this fish, in which 812.1 Mb of scaffolds were linked to 24 chromosomes with assistance of a high-density genetic map from a family of 104 full siblings and their parents. In addition, whole-genome resequencing of 96 representative individuals from diverse areas along the putative migration path clarified involvement of 150 genes in the migratory adaption. Based on integrative genomic and transcriptomic analyses, we determined that three Ca^{2+}-related pathways are critical for promotion of the migratory adaption. Meanwhile, we identified a large amount of molecular markers for distinguishing of migratory individuals and non-migratory freshwater residents. **Conclusions:** We assembled a chromosome-level genome for the Chinese tapertail anchovy. It provides a valuable genetic resource for understanding the migratory adaption and population genetics, and will benefit for aquaculture and management of this economically important fish.

Keywords: Chinese tapertail anchovy (*Coilia nasus*); genome sequencing; resequencing; genome assembly; migratory dimorphism; migratory adaptation

Introduction

Migration is one of the most spectacular events in nature. Every year, billions of animals start a seasonal movement to chase foods or mates, avoid predators, or escape from a

severe living environment. Hence, seasonal migration can influence the distribution of animals across space and time. Uncovering of related mechanisms for migratory adaptation is critical for understanding of evolutionary processes, as well as for facilitating management of stocks and conservation of endangered species. Many studies have aimed to understand this interesting phenomenon [1, 2]; however, the detailed molecular mechanisms have still been unknown.

Chinese tapertail anchovy, *Coilia nasus* (Figure 1a), is a valuable migratory fish with high economic importance in China. It can be classified into two groups according to their living habitats. One is the routine migratory group, with a wide distribution in sea areas close to Korea, China and Japan. In China, this fish is mainly captured from the Yellow Sea, East China Sea, and Yangtze River [3]. Its commercial importance is related to its nutritional contents and delicate flavor [4]. Similar to Pacific salmons (*Oncorhynchus* spp.) [2], *C. nasus* adults are known to migrate from February to April in each year anadromously to the Yangtze River before their final gonadal maturation in order to spawn in the middle and lower reaches of the Yangtze River (see more details in Figure 1b). There is a long distance of thousands of kilometers between the open ocean (for growth) and the natal stream (for reproduction) [5]. After spawning, the adult fish migrate downstream to the sea again; the juveniles, however, stay in the fresh water for 3~4 more months until they acquire the ability to tolerate sea water, and since then they follow the path of their parents to migrate downstream to the sea [6]. The other group has been reported to be always resident in some freshwater lakes during their whole lifetime [7]. This phenomenon, known as partial migration or migratory dimorphism [1], provides a good opportunity for us to obtain insights into migratory adaptation.

Many studies have investigated this process but most simply described the patterns of migratory dimorphism [8] or its occurrence in a given population [9]. These data

provided little information about the related genetic variations from the perspective of the whole genome. In addition, the detailed mechanisms related to migratory dimorphism in fish are disputed and poorly understood. Thus, in this study, we have generated a high-quality chromosome-level reference genome assembly of *C. nasus* by whole genome sequencing and digest restriction-site associated DNA (RAD) sequencing [10]. After resequencing of 96 individuals from diverse areas along the putative migration path (Figure 1b and Table 1), we identified numerous single nucleotide polymorphisms (SNPs) with high-evidence for examination of the detailed molecular divergence and adaptive mechanisms between the migratory and freshwater residential groups. Genome-wide identification of candidate genes for the migratory adaptation will benefit for biological and genetic studies of fish migration.

Results

Sequencing and assembly for a chromosome-level genome

We sequenced about 277.9 gigabases (Gb) of short reads (100-150 bp) by an Illumina Hiseq 2500 platform (Illumina, San Diego, CA, USA) and 68.6 Gb of long reads (an average of 14,743 bp) from a PacBio RSII platform (Pacific Biosciences, Menlo Park, CA, USA) (see more details in Supplementary Table 1). After removal of low-quality raw reads, we assembled a high-quality genome with a scaffold N50 and a contig N50 of 2.1 Mb and 1.6 Mb, respectively. Our genome assembly spanned about 870.0 Mb, which is consistent with the predicted genome size of 857.5 Mb based on a *K*-mer analysis [11] (Supplementary Figure 1 and Supplementary Table 2). A BUSCO (version 3.03, RRID:SCR_015008) [12] evaluation of our assembly was 90.1%, where C=87.1% [D=4.6%], F=3.0%, M=9.9%, and n=4584 (C: complete [D: duplicated], F: fragmented, M: missed, n:

genes), thereby suggesting a high level of completeness for the *C. nasus* assembly.

Meanwhile, we constructed a high-density genetic linkage map of *C. nasus* based on a RAD sequencing of a family of 104 full siblings with their parent pairs. Subsequently, we localized a total of 15,300 high-quality SNPs into 24 linkage groups with a genetic distance up to 7,651.0 cM. Finally, 93.3% of the assembled genome sequences (812.1 Mb/870.0 Mb) were allocated to the 24 putative pairs of chromosomes (Figure 2, Supplementary Figure 2).

Repeat sequences were predicted to comprise approximately 31.1% of the *C. nasus* genome (Supplementary Table 3). We classified these repeat sequences into several representative types, and observed that Simple and hAT repeat sequences were the most abundant types (accounting for 5.75% and 5.65%, respectively) in the genome assembly (see Supplementary Table 3). We also annotated 20,837 genes with an average length of 16.8 kb (Supplementary Table 4), of which 20,300 genes have fuctional assignments with public databases (Supplementary Table 5). Details of the chromosomal map and markers (Supplementary Table 6), density of genes, GC content, and repeat sequences are summarized in Figure 2.

Genome resequencing and identification of variations

Whole-genome resequencing generated approximately 4.5 billion of 125-bp paired-end reads (i.e., 684.0 Gb of raw data). The mapping ratio for each sample ranged from 64.0% to 71.0%, and the average mapping depth was determined as about 10 folds (Supplementary Table 7). In total, 39,415,696 high-confidence SNPs were called, and then they were annotated based on their positions in the chromosomes. Most of the SNPs (25.3 Mb, 64.1%) were identified in intergenic regions, while 1.31 Mb of the SNPs (33.3%) were distributed in intron regions, and only 1.0 Mb of the SNPs (2.6%) were presented in

coding regions. Among these SNPs within coding regions, we identified 472,322 synonymous SNPs and 545,212 non-synonymous SNPs (Supplementary Table 8).

In order to identify the detailed divergence at the genome level among the 96 examined individuals, we constructed a phylogenetic tree based on the entire SNP set. Interestingly, the tree demonstrated that these individuals could be divided clearly into two groups, in which 11 were freshwater residents and 85 were migratory individuals (Figure 1c). For confirmation of this grouping, we also employed electron probe microanalysis [13] to check whether these fish were migratory or not. As we reported previously, the migratory group can be discriminated based on the Sr (Strontium) and Ca (Calcium) signatures in otoliths [13-15]. Since different environment conditions can lead to variations in the Sr contents and Sr:Ca ratios in otoliths, we employed blue (Sr:Ca ratio ≤ 3.0), green or yellow (Sr:Ca ratio = 3.0~7.0), and red (Sr:Ca ratio > 7.0) regions in Figure 1d and Supplementary Figure 3 to represent fresh water, brackish water, and sea water patterns, respectively [3, 4]. It appears that our SNP set could clearly distinguish the divergence between these freshwater residents and migratory individuals (Figure 1c), which was validated by the electron probe microanalysis (Figure 1d). Therefore, this SNP set (a detailed list in Supplementary Table 8) can be used as genetic markers for a complement of the common performance of otoliths microstructures.

Identification of 150 genes related to the migratory adaption

We screened 661 windows with the top 5% Fst (fixation index for diversity differentiation) and ROD (reduction of diversity) values, where 150 functional genes were identified (Figure 3a, Supplementary Table 9). These genes had potentially undergone independent selection for involvement in the migratory adaptation. Interestingly, some of these selected genes were physically clustered in the assembled

genome. For example, among the 150 migratory adaptation-related genes, 90 (60.0%) were distributed on six chromosomes (Figure 3b, Supplementary Table 9), thereby suggesting that the migratory adaptation may require a preference for selected genes in adjacent locations. In particular, the chromosomes 23, 4, and 15 were the three main chromosomes related to the migratory adaptation, and 19 genes were localized on the chromosome 23 (Figure 3b). Moreover, genes with selective sweep signals were identified based on $\pi_{migration}/\pi_{freshwater}$ (Figure 3c), Fst, and ROD (Figure 3d) using a 5-kb sliding window between the 26th Mb and 31th Mb. Three migration-related genes, including *Tgfbr2*, *Smad4*, and *Gbp* were localized within this region (Figure 3d).

In order to further clarify the functions of these 150 genes, we performed GO (gene ontology) and pathway enrichments. These genes were predicted to participate in several important functions, such as "substrate-specific transporter activity" (GO:0022892), "ion transmembrane transporter activity" (GO:0015075), "cation channel activity" (GO:0005261), "potassium channel activity" (GO:0005267), and "neuropeptide hormone activity" (GO:0005184) (Supplementary Table 10). They were significantly enriched in 11 pathways (Supplementary Tables 11), which suggests that the migratory adaptation-related genes were clustered in terms of both physical (chromosomal) positions and physiological functions. Interestingly, three pathways related to Ca^{2+} metabolism were enriched, including the calcium signaling pathway, MAPK signaling pathway, and Wnt signaling pathway (Figure 4). These data indicate that Ca^{2+}-related pathways may play key roles in the adaptation to migration.

Differentially expressed genes (DEGs) in the Ca^{2+}-related pathways for the migratory group

We analyzed the variable sites in the 14 genes within the three Ca^{2+}-related pathways (red in Figure 4). In total, 277 non-synonymous SNPs were distributed in the coding

sequence regions, and their allele frequencies were significantly different in the two fish groups (Supplementary Table 12). In order to validate whether the DNA variations affected gene transcription, we quantified the mRNA changes for several randomly selected genes (Figure 5a–d) by real-time quantitative RT-PCR. Our results demonstrated that *Smad4* (Figure 5a), *Gbp* (Figure 5b), *Fzd1* (Figure 5d), *Tgfbr2* (Figures 3b, 3c, and 5e), and *Slc8a1* (Figure 5f) were transcribed more in the liver of migratory group than that of freshwater residents. Moreover, *Cacnalg* has a higher transcription level (Figure 5c) in the heart of the migratory group than the freshwater residents. We also compared the transcription values in the brains of the migratory and the residential groups, and 648 DEGs were identified (Supplementary Table 13). In particular, 27 genes were from the three Ca^{2+}-related pathways, and most of the genes (23) had higher transcription values in the migratory group than the freshwater residents (Figure 5g). It seems that the migratory group maintained the gene transcription of the three Ca^{2+}-related pathways at high levels for the migratory adaptation. In addition, the DNA variations may have caused changes in the tertiary structure of proteins to allow variable functions. For example, the 298V site in Tgfbr2 located in the protein kinase domain (Figure 5h), which catalyzes transfer of the gamma phosphate from nucleotide triphosphates to one or more amino acid residues in a protein substrate side chain, resulting in a conformational change to potentially affect the corresponding protein function [16, 17].

Discussion

Fish migration is an interesting natural phenomenon. The migratory adaptation mechanisms in fish have been studied from various perspectives, such as ecology, physiology, genetics, and morphology [1, 2]. However, they have rarely been examined

from a whole genome view. After analyzing the genome sequencing and resequencing data, we identified 150 candidate genes embedded in the selected sweep regions that are potentially involved in the migratory adaptation. It seems that the molecular mechanisms of the migratory adaptation can be interpreted at three major levels, including reproductive adaptation, long-distance migratory adaptation, and complex environmental adaption.

Genetic basis of reproductive adaptation

The main aim of migration is to spawn for a wide distribution of species. Thus, the migratory adaptation should first deal with endocrine and reproductive adaptation. In our previous study [18], we reported that the unsaturated fatty acid metabolism and steroid hormone biosynthesis are involved in the regulation of ovarian development in *C. nasus*. Among the 150 candidate genes identified in the present study, *Acoxl* is known to play an important role in the biosynthesis of unsaturated fatty acids (Supplementary Tables 9 and 10). As well as, four genes from oocyte meiosis and maturation pathways were included in this list of 150 genes (Supplementary Table 9 and 10), which were also potentially involved in the reproductive adaptation. In addition, several genes with selective sweep signals in the migratory group, including *Fzd1*, *Ppp2r1b*, *Cacna1a* and *Smad4*, that were well confirmed to affect the reproductive capacity of females and males by previous knockout experiments [19-24]. Hence, they are expected to play important roles in the reproductive adaptation for our migratory group.

Positive selection of candidate genes for the long-distance migratory adaptation

Migration group of *C. nasus* must undergo a long-term countercurrent migration, which requires a high athletic capacity. Some selective sweeping regions of the migratory group covered several important genes, such as *Atp2a3*, *Flnb* and *Acna1g*, that were associated

with cardiovascular, hematopoietic and muscle functions [25-27]; these genes could participate in the adaptation to this long distant migration. Moreover, genes related to nervous system development and spatial recognition, like *Egfr*, *Adcy1*, *Flnb*, *Acna1g* and *Tgfbr2*, also harbored selective sweep signals, suggesting that evolution of these genes could be important for the orientation recognition of open waters in the migratory group [28, 29]. In addition, fishes rarely feed during their migration [30, 31]. Several digestion- and metabolism-related genes (including *Tgfbr2*, *Smad4*, *Ryr2*, *Cacna1a*, *Pdgfrb*, and *Slc8a1*) have undergone the selective sweeping, which may contribute to the highly efficient digestion and metabolism in the migratory group [24].

Genetic adaptation to complex environments during migration

Salinity and osmotic pressure adaptations are essential for migration. It has been reported that the Ca^{2+} signaling pathway is important for regulation of osmotic pressure [32]. The critical 14 genes (red in Figure 4) in the list of 150 DEGs with selective sweep signals were significantly enriched in the three central Ca^{2+}-related pathways ($P<0.01$; Supplementary Tables 11 and 12). We also observed that DNA-level variations elevated the transcription of genes in these three pathways to affect their functions (Figure 5). These three central Ca^{2+}-related pathways have key roles for cell proliferation and osmotic pressure regulation [33-35]. We also found that six genes with strong selective sweep signals were significantly enriched in GO terms of metal and calcium ion transport ($P<0.05$; Supplementary Table 10), which could also be related to the salinity and osmotic pressure adaptation.

Meanwhile, some genes (such as *Flnb*, *Tgfbr2*, *Pdgfrb*, and *Smad4*; Supplementary Table 12) related to renal function and homeostasis also underwent selective sweeping, suggesting their potential contribution to the alternative adaptation of salty and

freshwater [36]. Previous studies showed that the visual and olfactory systems were essential for the migratory fishes [37, 38]. Interestingly, some visual and olfactory related genes were also identified among the 150 candidate genes in the migratory group of *C. nasus* (Supplementary Table 10).

Conclusions

In summary, we performed whole genome sequencing of the Chinese tapertail anchovy (*C. nasus*) and constructed a high-density genetic linkage map to generate a high-quality chromosomal map. In total, 96 individuals were collected over a range of 618 km during their reproductive migration for resequencing. Based on the resequencing data and otolith X-ray electron microprobe validation, we determined 11 individuals as freshwater residents whereas the remainders were migratory fishes. Our high-quality reference genome and big amount of resequencing data provide a good opportunity to examine the migration process, and also reveal a more comprehensive image of *C. nasus* population genetics that will facilitate practical aquaculture and management of this economically important fish. Identification of 150 candidate genes with a significant enrichment in three critical Ca^{2+}-related pathways supports the molecular mechanisms of the migratory adaptation at three major levels, including reproductive adaptation, long-distance migratory adaptation and complex environmental adaptation.

Materials and Methods

Sample collection and sequencing

A healthy female *C. nasus*, cultivated at our local base in Yixing city (Jiangsu Province, China) with a body weight of 167.0 g, was used for the whole genome

sequencing. Its skeletal muscle was collected and stored immediately in liquid nitrogen. Genomic DNA was extracted using Qiagen GenomicTip100 (Qiagen, Hilden, Germany). The traditional whole-genome shotgun sequencing strategy [39] was employed. We constructed three short-insert libraries (250, 500, and 800 bp) and four long-insert libraries (2, 5, 10, and 20 kb) according to the manufacturer’s instructions (Illumina). A total of 277.9 Gb of raw paired-end sequencing reads (Supplementary Table 1) were generated by an Illumina HiSeq 2500 platform.

Meanwhile, AMPure PB magnetic beads (Pacific Biosciences) were utilized to concentrate the extracted high-quality genomic DNA for library construction with the SMRTbell template prep kit 2.1 (Pacific Biosciences). Sequencing was performed on a Pacific Bioscience (PacBio) Sequel platform. A total of about 68.6 Gb of PacBio raw reads (Supplementary Table 1) were generated.

Based on the putative migration path of *C. nasus*, we sampled 96 individuals from different localities in the Yellow Sea, Chongming, Nantong, Jingjiang, Anqing, Hukou, and Duchang (see more details in Figure 1b and Table 1). Genomic DNA was isolated from skeletal muscle using Qiagen GenomicTip100. Resequencing libraries (average insert of about 350 bp) were constructed for each genomic DNA pool of the 96 individuals, and 2 × 150 bp paired-end reads were generated by an Illumina HiSeq2500 platform.

All animal experiments in this study were performed in accordance with the guidelines of the Animal Ethics Committee and were approved by the Institutional Review Board on Bioethics and Biosafety of BGI (No. 18134).

Estimation of genome size and assembly of the genome

The *C. nasus* genome size (G) was estimated by a k-mer analysis [11] according to the

following formula: G = Kmer_num/Kmer_depth, where Kmer_num is the total number of reads and Kmer_depth represents the frequency of occurring more frequently than others. The estimated genome size of *C. nasus* was 857.5 Mb (Supplementary Table 2).

SOAP*denovo*2 (version 2.04.4; RRID:SCR_014986) [40] with optimized parameters (pregraph -K 27 -d 1; contig -M 1; scaff -F -b 1.5 -p 16) was employed to construct contigs and original scaffolds based on the sequenced reads. Subsequently, total reads were mapped onto the contigs for scaffolding according to the long-insert paired-end information, which lead to linkage of contigs to scaffolds in a stepwise manner. We then used about 109.2 Gb of clean reads from the short-insert (250, 500, and 800 bp) libraries to fill gaps in scaffolds with GapCloser (v1.12-r6; RRID:SCR_015026; default parameters and -p set at 25). Finally, we generated the first version of genome assembly that contained 786.3 Mb of scaffolds with scaffold and contig N50 values of 1.8 Mb and 19.0 kb respectively.

To improve the *de novo* assembly, we also sequenced 68.6 Gb of PacBio reads. We firstly used Platanus (version 1.2.1, RRID:SCR_015531) [41] to generate a *de novo* assembly with a total of 1.0 Gb in length and a contig N50 of 764 bp using Illumina reads from the short-insert (250, 500, and 800 bp) libraries. Subsequently, all PacBio reads and above assembled contigs were used for further assembling by utilizing of the DBG2OLC pipeline (default version) [42] with following parameters: LD10, MinLen 200, KmerCovTh 6, MinOverlap 80, AdaptiveTh 0.012, and RemoveChimera 1. This step generated a preliminary genome assembly of 1.0 Gb with a contig N50 of 1.5 Mb. A polishing step of this assembly was then performed using Illumina reads from the short-insert libraries. These reads were mapped onto the contigs using BWA-MEM (version 0.6.2, RRID:SCR_010910) [43]. Pilon (version 1.22, RRID:SCR_014731) [44] was also used to correct the assembly according to the alignment. SSPACE (version 3.0,

RRID:SCR_005056) [45] was then used for generation of scaffolds with the Illumina reads from the long-insert libraries (2 kb, 5 kb, 10 kb and 20 kb). In this step, we obtained an assembly of 1.0 Gb with a scaffold N50 of 1.6 Mb. We then used Redundans (version 0.14a) [46] with parameters (--identity 0.3 --overlap 0.3 --minLength 1000) to remove redundant scaffolds caused by the high heterozygosity of *C. nasus* genome. After this step, we obtained a final assembly of 870.0 Mb with improved scaffold and contig N50 values of 2.1 Mb and 1.6 Mb respectively.

Genome annotation

For repeat annotation, Repeat Modeller (version 1.04, RRID:SCR_015027) [47] and LTR_FINDER (version 1.06, RRID:SCR_015247) [48] were employed to construct a *de novo* repeat library with default parameters. RepeatMasker (version 3.2.9; RRID:SCR_012954) [49] was then used to search the repeat sequences against Repbase TE (version 14.04) [50] and the *de novo* repeat libraries in order to identify known and novel transposable elements (TEs) in *C. nasus* genome. We identified tandem repeats using Tandem Repeat Finder (version 4.04) [51], where the core parameters were set as "Match = 2, Mismatch = 7, Delta = 7, PM = 80, PI = 10, Minscore = 50, and MaxPerid = 2000." Furthermore, the relevant TE proteins were screened in the *C. nasus* assembly using RepeatProteinMask (version 3.2.2) [49].

To predict gene structures and functions, we employed a combined annotation pipeline of three separate approaches, including homology, *de novo*, and transcriptome-based annotations. For the homology annotation, protein sequences from zebrafish, Japanese fugu, spotted green pufferfish, Japanese medaka, and stickleback (Ensembl release 75) were downloaded to map onto the *C. nasus* genome using Blat (e-value ≤ 1E-5; version 319, RRID:SCR_011919) [52]. Genewise (version 2.2.0, RRID:SCR_015054) [53]

was then employed to predict the potential gene structures based on all the alignments generated from the previous step. Short genes (less than 150 bp) and prematurely terminated or frame-shifted genes were discarded. For the *de novo* annotation, 1,000 complete genes were randomly chosen from the homology annotation set to train parameters for AUGUSTUS (version 3.0.2, RRID:SCR_008417) [54]. Repeat regions were masked by "N" in our genome assembly. We then utilized AUGUSTUS to make *de novo* predictions based on the repeat-masked genome assembly. We filtered the *de novo* annotation results using the same method for the homology prediction. For the transcriptome-based annotation, total RNA was extracted from the muscle and liver tissues from the same female fish for whole genome sequencing. The sequencing reads were aligned onto the genome assembly using HISAT2 (version 0.1.6, RRID:SCR_015530) [55]. We sorted and integrated these alignments, and then employed Cufflink (version 2.2.1, RRID:SCR_014597) [56] to identify potential gene structures. The results obtained by all three annotation methods were merged to produce a comprehensive and non-redundant gene set using Maker (version 2.31.8, RRID:SCR_005318) [57].

All the protein sequences obtained from the Maker results were mapped onto the SwissProt and TrEMBL databases [58] by BLASTP (version 2.2.25, RRID:SCR_001010) [59] with an E-value ≤ 1e-5 to find the best hit for each protein. We also employed InterProScan (version 4.7, RRID:SCR_005829) [60] to align the protein sequences against other public databases, including Pfam [61], PRINTS [62], ProDom [63] and SMART [64], in order to determine the known motifs and domains in our protein sequences. Finally, 20,300 genes were proved to contain at least one functional assignment from public databases, including Swiss-Prot and TrEMBL [58], Gene Ontology (GO; [65]) and Kyoto Encyclopedia of Genes and Genomes (KEGG) [66] (Supplementary Table 5).

RAD sequencing and genotyping

RAD sequencing [10] was performed to generate a set of SNP markers from a full-sib family F1 group. In brief, the procedure was described as follows.

(1) *DNA extraction and sequencing*. Genomic DNA from the 104 offspring individuals and their parents was separately extracted from the fin clips using a Mag Attract HMW DNA Kit (Qiagen, Gaithersburg, MD, USA). *Pst*I restriction enzyme was used for digestion of DNA. We then constructed the RAD sequencing libraries, which were subsequently sequenced on an Illumina HiSeq 2500 platform. The adapters of raw reads and those reads with low quality were filtered with a local perl script.

(2) *SNP calling*. We used BWA-MEM (version:0.7.12, RRID:SCR_010910) [43] to align cleaned reads upon the second version of genome assembly. Subsequently, we employed GATK (version: 3.1, RRID:SCR_001876) [67] to perform SNP calling. Related parameters for GATK was set as "QD < 2.0 || FS > 60.0 || MQ <40.0 || MQRankSum < -12.5 || ReadPosRankSum < -8.0".

Construction of the genetic linkage map and chromosomal map

JoinMap (version 4.1, RRID:SCR_009248) [68] with logarithm of odds values ranging from 2 to 12 was employed to evaluate the map distance under regression mapping algorithm. Subsequently, we constructed a high-density genetic linkage map with 24 linkage groups, which is consistent with the results of a previous report [69].

Based on the SNP markers and genetic linkage map, we obtained a preliminary chromosomal-level assembly. Locations of the scaffolds in each chromosome were fixed according to the following rules. For the scaffolds with sufficient SNP markers (more than two), we chose two markers with the highest quality to determinate their location and direction. However, directions of those scaffolds with insufficient SNP markers (only one) were not fixed, but instead they were placed directly onto the chromosomes.

In the first genetic map based on the first version of genome assembly (assembled with only the Illumina reads), we localized a total of 2,988 high-quality SNPs into 24 linkage groups with a genetic distance up to 3,988.5 cM. Finally, 86.9% of the genome sequences (683.3 Mb/786.3 Mb) were allocated to the 24 putative pairs of chromosomes (first version). Subsequently, we generated an improved high-density genetic map based on the second version of genome assembly. A total of 15,300 high-quality SNPs were anchored into the 24 linkage groups with a genetic distance up to 7,651.0 cM. In this version, 93.3% of the genome sequences (812.1 Mb/870.0 Mb) were assembled into the 24 putative pairs of chromosomes.

SNP calling and phylogenetic analysis

The resequencing reads were aligned onto our genome assembly (first version) using BWA-MEM (version 0.7.1, RRID:SCR_010910) [43]. SNP calling was performed using a standard GATK (version 3.1, RRID:SCR_001876) [67]. Quality filtering was realized for the raw variant calls using GATK with the following cut-offs: QD < 2.0, MQ < 40.0, FS > 60.0, MQRankSum ≤ 12.5, ReadPosRankSum$< –8.0$, and DP < 100. We excluded variants with more than 10% missing data and used a minor allele frequency filter of 10%. We then employed SnpEff (version 3.4, RRID:SCR_005191) [70] to annotate the genetic variants and categorize these variants into coding (synonymous and nonsynonymous), upstream/downstream, and intronic/intergenic classes. PLINK (version 1.07, RRID:SCR_001757) [71] was applied to calculate the genetic distances between individuals, which were subsequently used to generate neighbor-joining trees with fneighbor (PHYLIPNEW v3.69.650 within the package of EMBOSS v6.6.0.0, RRID:SCR_006244) [72].

Identification of selective sweep regions

Reduction of diversity was defined as ROD = 1− $\pi_{freshwater}/\pi_{migration}$, in which the $\pi_{freshwater}$ and $\pi_{migration}$ are the average numbers of nucleotide differences per site [73] from the freshwater and the migratory groups respectively. We calculated the Fst and ROD values in a sliding window of 5 kb along the genome assembly using the entire SNP set. Genomic regions located in the top right corner of Figure 3a, corresponding to a 5% significant level of the FST and ROD values (above 0.79 and 0.59, respectively), were considered as the selective sweep regions.

Transcriptome analysis for freshwater and migratory individuals and validation by quantitative RT-PCRs

For transcriptome sequencing, total RNA was extracted from brain tissues of three randomly selected individuals in the migratory or freshwater groups using TRIzol reagent (Invitrogen, Carlsbad, CA, USA). We generated 125-bp paired-end Illumina reads on a HiSeq 4000 platform for transcriptome sequencing. These sequenced RNA reads were aligned onto our reference genome (First version) using HISAT2 (version 0.1.6, RRID:SCR_015530) [55]. Unigene expression values were calculated with Cufflink (version 2.2.1, RRID:SCR_014597) [56]. The Cuffdiff package in Cufflink [56] was employed to identify the differentially expressed genes (DEGs). Finally, we identified the enriched GO terms for these DEGs using the Enrich Pipeline as described previously [74].

For the quantitative RT-PCRs, samples were obtained from five individuals in each group, and total RNA was extracted separately with TRIzol reagent (Invitrogen, Carlsbad, CA, USA). First-strand cDNA was subsequently synthesized using a PrimeScript™ RT reagent kit with gDNA Eraser (Takara, Kusatsu, Shiga, Japan), and 18S RNA was used as the internal control. Sequences of the primer pairs are provided in Supplementary Table 14. Transcription of the target genes was calculated as the relative

increase according to the $2^{-\Delta\Delta CT}$ method [75]. Differences in the mRNA levels were compared by the Student's *t*-test and those with $p \leq 0.05$ were considered significant.

Measurement of Sr and Ca contents in otoliths

The Sr and Ca contents in otoliths were measured as described in our previous report [3]. In brief, the otoliths were embedded in epoxy resin (EpoFix, Struers, Copenhagen, Denmark) for grinding and polishing to expose their cores with an automated grinding machine (Roto Pol-35, Struers, Kentucky, USA). After cleaned in an ultrasonic bath, rinsed by deionized water, and carbon-coated with a high-vacuum evaporator (JEE-420, JEOL Ltd., Tokyo, Japan), the samples were measured using a wave-dispersive X-ray electron probe Micro-analyzer (JXA-8100, JEOL Ltd, Welwyn Garden City, UK). Tausonite ($SrTiO_3$) and calcite ($CaCO_3$) were used as the internal standards.

Supplementary materials

Supplementary data associated with this article can be found in the online version.

Availability of supporting data

Genome assemblies reported here have been deposited at the NCBI GenBank under the project ID PRJNA421870. Genome *denovo*, resequencing, RAD and transcriptome sequencing data have been deposited at the NCBI Short Read Archive (SRA) under the project ID PRJNA422339.

Abbreviations

Adcy1, Adenylate Cyclase 1; *Acoxl*, Acyl-coenzyme A oxidase-like protein; *Atp2a3*, ATPase Sarcoplasmic/Endoplasmic Reticulum Ca2+ Transporting 3; Ca, Calcium; *Cacna1a*, Calcium Voltage-Gated Channel Subunit Alpha1 A; *Cacnalg*, Voltage-dependent T-type calcium channel subunit alpha-1G; DEGs, differentially expressed genes; *Egfr*, Epidermal Growth Factor Receptor; Fst, Fixation index for diversity differentiation; *Flnb*, Filamin B;

Fzd1, Frizzled-1; Gb, gigabase; Gbp, GSK-3-binding protein; GO, gene ontology; KEGG, Kyoto Encyclopedia of Genes and Genomes; *Pdgfrb*, Platelet Derived Growth Factor Receptor Beta; Ppp2r1b, Serine/threonine-protein phosphatase 2A 65 kDa regulatory subunit A beta isoform; RAD, restriction-site associated DNA; ROD, reduction of diversity; *Ryr2*, Ryanodine Receptor 2; *Slc8a1*, Solute Carrier Family 8 Member A1; *Smad4*, SMAD Family Member 4; SNP, single nucleotide polymorphism; Sr, strontium; TE, transposable element; *Tgfbr2*, Transforming Growth Factor Beta Receptor 2.

Competing interests

The authors declare no competing financial interests.

Funding

This study was supported by grants from the National Natural Science Foundation of China (Nos. 31672643, 31372533, 31502152), Three New Projects of Agricultural Aquaculture Program of Jiangsu Province (No. Y2018-17), and the Special Fund of Jiangsu Province for the Transformation of Scientific and Technological Achievements (No. BA2015167).

Author contributions

P.X. conceived the study and designed the project. G.X. managed the project. K.L., D.X., Y.W., Q.L., N.S., J. C., C. S., Y. Z. and Z.J.N. prepared the all samples used in this study. C.B. performed genome assembly, annotation, resequencing data analyses and transcriptome expression calculation. J.L. constructed the genetic map and chromosomal map. Y.H. and Y.L. implemented phylogenetic analysis. H.L., J.Y. and T.J. measured the Sr and Ca contents of otoliths. P.X., Q.S., G.X., C.B., J.L., X.Y, R.G., W.G. and J.X. discussed the data. C.B., G.X. and J.L. wrote the manuscript. Q.S., G.X and P.X. revised the manuscript. All authors contributed to data interpretation.

References

1. Chapman BB, Hulthen K, Brodersen J, et al. Partial migration in fishes: causes and consequences. J Fish Biol 2012; **81**:456–478.
2. Ueda H. Physiological mechanism of homing migration in Pacific salmon from behavioral to molecular biological approaches. Gen Comp Endocrinol 2011; **170**(2):222–232.
3. Jiang T, Yang J, Lu MJ, et al. Discovery of a spawning area for anadromous *Coilia nasus* Temminck et Schlegel, 1846 in Poyang Lake, China. J Appl Ichthyol 2017; **33**:189–192.
4. Chen TT, Jiang T, Liu HB, et al. Do all long supermaxilla-type estuarine tapertail anchovies (*Coilia nasus* Temminck et Schlegel, 1846) migrate anadromously? J Appl Ichthyol 2017; **33**:270–273.
5. Jiang T, Yang J, Liu H, et al. Life history of *Coilia nasus* from the Yellow Sea inferred from otolith Sr:Ca ratios. Environ Biol Fish 2012; **95**:503–508.
6. Li WX, Song R, Wu SG, et al. Seasonal Occurrence of Helminths in the Anadromous Fish *Coilia nasus*. J Para 1937; **97**:192–196.
7. Liu D, Li Y, Tang W, et al. Population structure of *Coilia nasus* in the Yangtze River revealed by insertion of short interspersed elements. Biochem Syst Ecol 2014; **54**:103–112.
8. Secor DH, Kerr L. Lexicon of life cycle diversity in diadromous and other fishes. Am Fish So Sym 2009; **69**:537–556.
9. Chapman BB, Skov C, Hulthén K, et al. Partial migration in fishes: definitions, methodologies and taxonomic distribution. J Fish Biol 2012; **81**:479–499.
10. Yu H, You X, Li J, et al., A genome-wide association study on growth traits in orange-spotted grouper *(Epinephelus coioides*) with RAD-seq genotyping. Sci China Life Sci 2018; **61**: 934-946.
11. Song L, Bian C, Luo Y, et al. Draft genome of the Chinese mitten crab, *Eriocheir sinensis*. GigaScience 2016; **5**:5.
12. Simao FA, Waterhouse RM, Ioannidis P, et al. BUSCO: assessing genome assembly and annotation completeness with single-copy orthologs. Bioinformatics, 2015;**31**:3210-3212.
13. Brown RJ, Severin KP. Otolith chemistry analyses indicate that water Sr:Ca is the primary factor influencing otolith Sr:Ca for freshwater and diadromous fish but not for marine fish. Can J Fish Aquat Sci 2009; **66**:1790–1808.
14. Limburg KE, Olson C, Walther Y, et al. Tracking Baltic hypoxia and cod migration over millennia with natural tags. P Natl Acad Sci USA 2011; **108**:177–182.
15. Yang J. Jiang T, Liu H. Are there habitat salinity markers of the Sr:Ca ratio in the otolith of wild diadromous fishes? A literature survey. Ichthyol Res 2011; **58**:291–294.

16. Knighton DR, Zheng JH, Eyck LT, et al. Crystal structure of the catalytic subunit of cyclic adenosine monophosphate-dependent protein kinase. Science 1991; **253**:407–414.
17. Kohli G, Hu S, Clelland E, et al. Cloning of transforming growth factor-β1 (TGF-β1) and its type II receptor from zebrafish ovary and role of TGF-β1 in oocyte maturation. Endocrinology 2003; **144**:1931–1941.
18. Xu G, Du F, Li Y, et al. Integrated application of transcriptomics and metabolomics yields insights into population-asynchronous ovary development in *Coilia nasus*. Sci Rep 2016; **6**:31835.
19. Lapointe E, Boyer A, Rico C, et al. FZD1 regulates cumulus expansion genes and is required for normal female fertility in mice. Biol Reprod 2012; **87**:1–12.
20. Dickinson ME, Flenniken AM, Ji X, et al. High-throughput discovery of novel developmental phenotypes. Nature 2016; **537**:508–514.
21. Zwingman TA, Neumann PE, Noebels JL, et al. Rocker is a new variant of the voltage-dependent calcium channel gene Cacna1a. J Neurosci 2001; **21**:1169–1178.
22. Miki T, Zwingman TA, Wakamori M, et al. Two novel alleles of tottering with distinct Ca(v)2.1 calcium channel neuropathologies. Neurosci, 2008;**155**:31-44.
23. Yamaguchi T, Kato M, Fukui M, et al. Rolling mouse Nagoya as a mutant animal model of basal ganglia dysfunction: determination of absolute rates of local cerebral glucose utilization. Brain Res 1992; **598**:38–44.
24. Alberici P, Jagmohan-Changur S, De Pater E, et al. Smad4 haploinsufficiency in mouse models for intestinal cancer. Oncogene 2006; **25**:1841–1851.
25. Elaib Z, Adam F, Berrou E, et al., Full activation of mouse platelets requires ADP secretion regulated by SERCA3 ATPase-dependent calcium stores. Blood 2016; **128**:1129-1138.
26. Zhou X, Tian F, Sandzen J, et al., Filamin B deficiency in mice results in skeletal malformations and impaired microvascular development. Proc Natl Acad Sci USA 2007; **104**:3919-3924.
27. Yamaguchi N, Takahashi N, Xu L, et al., Early cardiac hypertrophy in mice with impaired calmodulin regulation of cardiac muscle Ca release channel. J Clin Invest 2007; **117**:1344-53.
28. Threadgill DW, Dlugosz AA, Hansen LA, et al. Targeted disruption of mouse EGF receptor: effect of genetic background on mutant phenotype. Science 1995; **269**:230–234.
29. Koitabashi N, Bedja D, Zaiman AL, et al. Avoidance of transient cardiomyopathy in cardiomyocyte-targeted tamoxifen-induced MerCreMer gene deletion models. Circ Res 2009; **105**:12–15.
30. Neverman D., Wurtsbaugh WA. The thermoregulatory function of diel vertical migration for a juvenile fish, *Cottus extensus*. Oecologia 1994; **98**:247–256.
31. Liu SH, Zhao-Li XU, Tian FG. Study on feeding habit of Coilia mystus in Yangtze River Estuary. J Shanghai Ocean U 2012; **21**:589–97.

32. Cooke CJ, Smith CJ, Newton RP, et al., Binding saturation analysis of inositol-1,4,5-trisphosphate in suspension cultures of lucerne cells. Biochem Soc Trans 1991; **19**:359–359.
33. Calcium Signaling Pathways. Biophys J 2008; **94**:150–157.
34. Seger R, Krebs EG. The MAPK signaling cascade. FASEB J 1995; **9**:726–735.
35. Komiya Y, Habas R. Wnt signal transduction pathways. Organogenesis 2008; **4**:68–75.
36. Jianying NI. Rencular structural indices and urinary concentrating capacity of Neophocaena phocaenoides. Acta Zool Sin 1988; **3**:2–3.
37. Wang FY, Fu WC, Wang IL, et al. The giant mottled eel, Anguilla marmorata, uses blue-shifted rod photoreceptors during upstream migration. PLoS ONE 2014; **9**:e103953.
38. Gardiner JM, Whitney NM, Hueter RE. Smells like home: The role of olfactory cues in the homing behavior of Blacktip sharks, Carcharhinus limbatus. Integr Comp Biol 2015; **55**:495–506.
39. Lin Q, Qiu Y, Gu R, et al., Draft genome of the lined seahorse, *Hippocampus erectus.* Gigascience, 2017; **6**:1-6.
40. Luo R, Liu B, Xie Y, et al. Erratum: SOAPdenovo2: an empirically improved memory-efficient short-read de novo assembler. Gigascience 2015; **4**:30.
41. Kajitani R, Toshimoto K, Noguchi H, et al. Efficient de novo assembly of highly heterozygous genomes from whole-genome shotgun short reads. Genome Res 2014; **24**:1384–1395.
42. Ye C, Hill CM, Wu S, et al. DBG2OLC: Efficient assembly of large genomes using long erroneous reads of the third generation sequencing technologies. Sci Rep 2016; **6**:31900.
43. Li H., Durbin R. Fast and accurate short read alignment with Burrows-Wheeler transform. Bioinformatics 2009; **25**:1754–1760.
44. Walker BJ, Abeel T, Shea T, et al. Pilon: An integrated tool for comprehensive microbial variant detection and genome assembly improvement. PLoS ONE 2014; **9**:e112963.
45. Boetzer M, Henkel CV, Jansen HJ, et al. Scaffolding pre-assembled contigs using SSPACE. Bioinformatics 2011; **27**:578–579.
46. Pryszcz LP, Gabaldon T. Redundans: an assembly pipeline for highly heterozygous genomes. Nucleic Acids Res 2016; **44**:113-113.
47. Chen N. Using RepeatMasker to identify repetitive elements in genomic sequences. Curr Protoc Bioinformatics 2004; **Chapter 4**:Unit 4 p.10.
48. Xu Z, Wang H. LTR_FINDER: an efficient tool for the prediction of full-length LTR retrotransposons. Nucleic Acids Res 2007; **35**:265–268.
49. Tarailo-Graovac M, Chen N. Using RepeatMasker to identify repetitive elements in genomic sequences. Curr Protoc Bioinformatics 2009; **Chapter 4**:Unit 4 p.10.
50. Jurka J, Kapitonov VV, Pavlicek A, et al. Repbase Update, a database of eukaryotic repetitive elements. Cytogenet Genome Res 2005; **110**:462–467.

51. Benson G. Tandem repeats finder: a program to analyze DNA sequences. Nucleic Acids Res 1999; **27**:573–580.
52. Bhagwat M, Young L, Robison RR. Using BLAT to find sequence similarity in closely related genomes. Curr Protoc Bioinformatics 2012; **Chapter 10**: Unit10 p.8.
53. Birney E, Clamp M, Durbin R. GeneWise and Genomewise. Genome Res 2004; **14**:988–995.
54. Stanke M, Keller O, Gunduz I, et al. AUGUSTUS: ab initio prediction of alternative transcripts. Nucleic Acids Res 2006; **34**:435–439.
55. Kim D, Langmead B, Salzberg SL. HISAT: a fast spliced aligner with low memory requirements. Nat Methods 2015; **12**:357–360.
56. Trapnell C, Hendrickson DG, Sauvageau M, et al. Differential analysis of gene regulation at transcript resolution with RNA-seq. Nat Biotechnol 2013; **31**:46–53.
57. Cantarel BL, Korf I, Robb SM, et al. MAKER: an easy-to-use annotation pipeline designed for emerging model organism genomes. Genome Res 2008; **18**:188–196.
58. Bairoch A, Apweiler R. The SWISS-PROT protein sequence database and its supplement TrEMBL in 2000. Nucleic Acids Res 2000; **28**:45–48.
59. Mount DW. Using the Basic Local Alignment Search Tool (BLAST). CSH Protoc 2007; **2007**:top17.
60. Zdobnov EM, Apweiler R. InterProScan--an integration platform for the signature-recognition methods in InterPro. Bioinformatics 2001; **17**:847–848.
61. Finn RD, Tate J, Mistry J, et al., The Pfam protein families database. Nucleic Acids Res 2008; **36**:281-288.
62. Attwood TK, Croning MD, Flower DR, et al., PRINTS-S: the database formerly known as PRINTS. Nucleic Acids Res 2000; **28**: 225-227.
63. Bru C, Courcelle E, Carrere S, et al., The ProDom database of protein domain families: more emphasis on 3D. Nucleic Acids Res 2005; **33**: 212-215.
64. Letunic I, Copley RR, Pils B, et al., SMART 5: domains in the context of genomes and networks. Nucleic Acids Res 2006; **34**: 257-260.
65. Ashburner M, Ball CA, Blake JA, et al. Gene ontology: tool for the unification of biology. The Gene Ontology Consortium. Nat Genet 2000; **25**:25–29.
66. Kanehisa M, Goto S. KEGG: kyoto encyclopedia of genes and genomes. Nucleic Acids Res 2000; **28**:27–30.
67. Mckenna A, Hanna M, Banks E, et al. The Genome Analysis Toolkit: a MapReduce framework for analyzing next-generation DNA sequencing data. Genome Res 2010; **20**:1297–1303.
68. Stam, P., Construction of integrated genetic linkage maps by means of a new computer package: Join Map. The Plant J, 2005;**3**:739-44.
69. Xu S, Fu G, Wu H, et al. Chromosome karyotype analysis of *Coilia nasus*. Guangdong Agr Sci 2014; **7**:155–157.
70. Cingolani P, Platts A, Wang L, et al. A program for annotating and predicting the effects of single nucleotide polymorphisms, SnpEff: SNPs in the genome of Drosophila melanogaster strain w1118; iso-2; iso-3. Fly 2012; **6**:80–92.

71. Purcell S, Neale B, Todd-Brown K, et al. PLINK: a tool set for whole-genome association and population-based linkage analyses. Am J Hum Genet 2007; **81**:559–575.

72. Retief J.D. Phylogenetic analysis using PHYLIP. Methods Mol Biol 2000; **132**:243–258.

73. Berg PR, Jentoft S, Star B, et al. Adaptation to Low Salinity Promotes Genomic Divergence in Atlantic Cod (*Gadus morhua* L.). Genome Biol Evol 2015; **7**:1644–1663.

74. Chen S, Yang P, Jiang F, et al. De novo analysis of transcriptome dynamics in the migratory locust during the development of phase traits. PLoS One 2010; **5**:e15633.

75. Livak KJ, Schmittgen TD. Analysis of relative gene expression data using real-time quantitative PCR and the 2(-Delta Delta C(T)) Method. Methods 2001; **25**:402–408.

Figures legends

Figure 1. Seasonal migration and migratory dimorphism of the Chinese tapertail anchovy. (**a**) A representative image of this economically important fish. (**b**) Geographic distribution of the collected samples along the putative migration route. The red five pointed stars represent the sample collection sites (see more details in Table 1) and the green arrows indicate the direction of migration. (**c**) A Neighbor-joining phylogenetic tree constructed with genome-wide SNPs. The scale bar represents the similarity level. (**d**) Representative X-ray intensity maps of the Sr content in the otoliths of *C. nasus*. The constant blue color represents the freshwater residential pattern, while the alternative blue and green colors indicate the migratory pattern.

Figure 2. **A** Circos figure of the genome assembly. The rings from the outside to the inside are in the order of (**A**) pseudo-chromosomes, (**B**) a genetic map, (**C**) a heat map of gene density (in orange) in 100 kb of non-overlapping windows, (**D**) a line chart of the genome GC content in 100 kb of non-overlapping windows, and (**E**) a heatmap of repeat density (in violet) in 100 kb of non-overlapping windows. Syntenic blocks are connected with navy lines and each line indicates one paralog gene pair in the assembled genome.

Figure 3. Comparisons of selection sweep regions in the freshwater residential and migratory groups. (**a**) Distributions of ROD and Fst values in 5-kb non-overlapping windows. Red dots denote windows with the top 5% ROD and Fst values. (**b**) Migratory adaptation related genes distributed on 11 chromosomes. Examples of genes (**c, d**) with selection sweep signals identified by $\pi_{migration}/\pi_{freshwater}$, Fst, and ROD values using a 5-kb sliding window. Blue and red lines represent the $\pi_{migration}$ and $\pi_{freshwater}$, respectively. Dashed lines denote the threshold of top 5%.

Figure 4. Three enriched Ca^{2+}-related pathways. The genes highlighted in red were positively selected for the migratory adaptation. The green lines and arrows indicate

positive regulation and the red ones indicate negative regulation. Interestingly, 14 of the selected genes (highlighted in red) potentially participate in the three critical Ca^{2+}-related pathways, including calcium signaling pathway, MAKP signaling pathway, and Wnt signaling pathway.

Figure 5. Representative mRNA transcription and protein structural changes in the selected genes within the three Ca^{2+}-related pathways. (**a–f**) Quantitative RT-PCR validation of the mRNA transcription differences in six representative genes. (**g**) A heatmap of DEGs in the three Ca^{2+}-related pathways based on the brain transcriptome. (**i**) Changes in the tertiary protein structure of Tgfr2.

Tables

Table 1. Summary of sample information for the genome resequencing.

**Type**	**Locality**	**Sample**	**No.**	**Position**
**Sea**	Yellow Sea	S	15	N 31°30′ E 122°24′
**River**	Chongming	E	15	N 31°46′ E 121°7′
	Nantong	2R	15	N 31°58′ E 120°49′
	Jingjiang	3R	11	N 31°56′ E 120°14′
	Anqing	4R	13	N 30°30′ E 117°4′
**Lake**	Hukou	5R	13	N 29°44′ E 116°12′
	Duchang	6R	14	N 29°14′ E 116°11′

Table 2. Statistics of the genome assembly of *C. nasus*.

**Genome assembly**	**Parameter**
Contig N50 (Mb)	1.6
Contig number (>100 bp)	1,327
Scaffold N50 (Mb)	2.1
Scaffold number (>100 bp)	727
Total length (Mb)	870.0
Genome coverage (×)	404.4
The longest scaffold (Mb)	12.0
**Genome annotation**	
Protein-coding gene number	20,837
Mean transcript length (bp)	16,775.5
Mean exons per gene	10.1
Mean exon length (bp)	1,759.7
Mean intron length (bp)	1,476.0

Figure 1 Click here to download Figure Figure 1.pdf

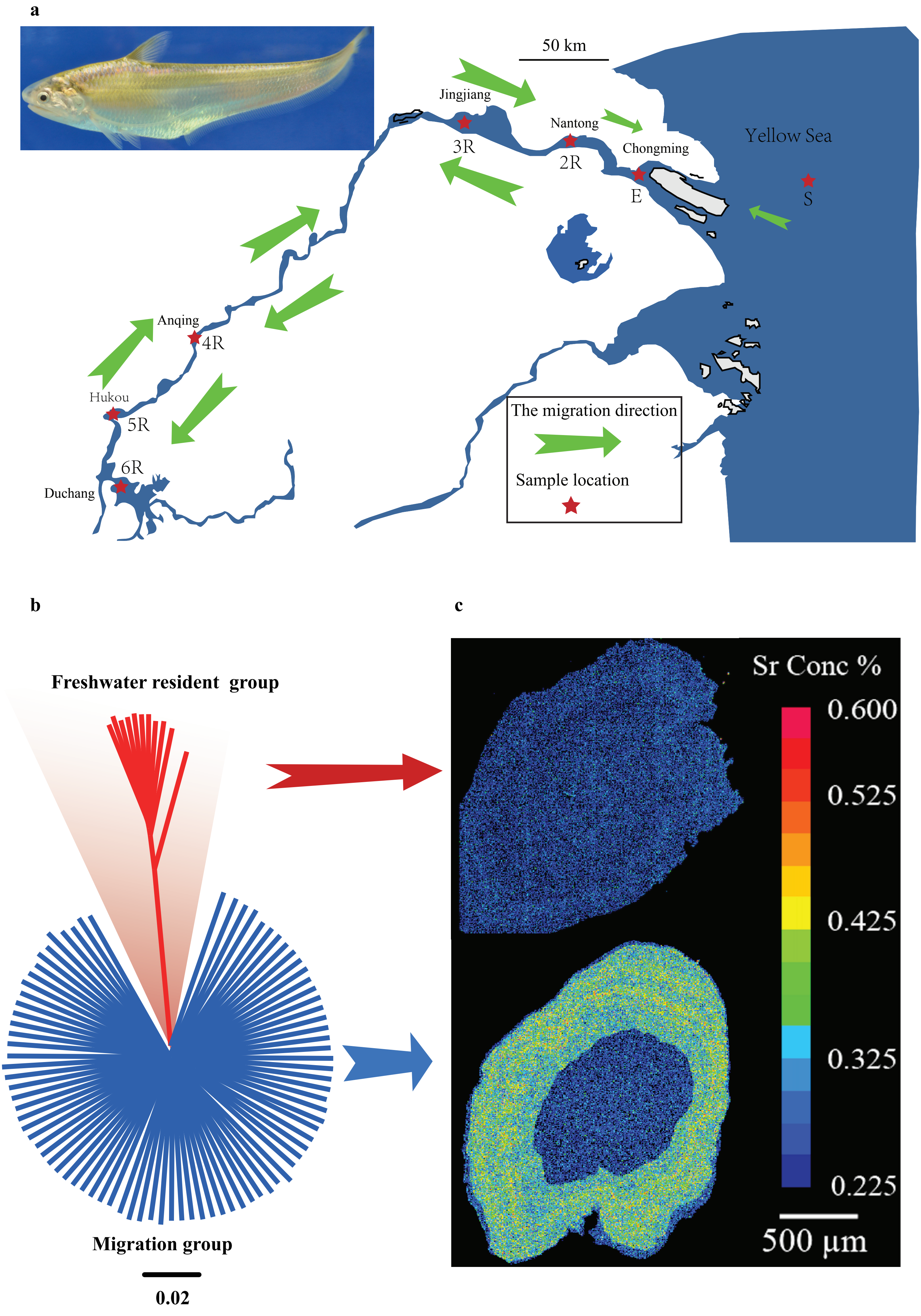

Figure 2

Click here to download Figure Figure 2.pdf

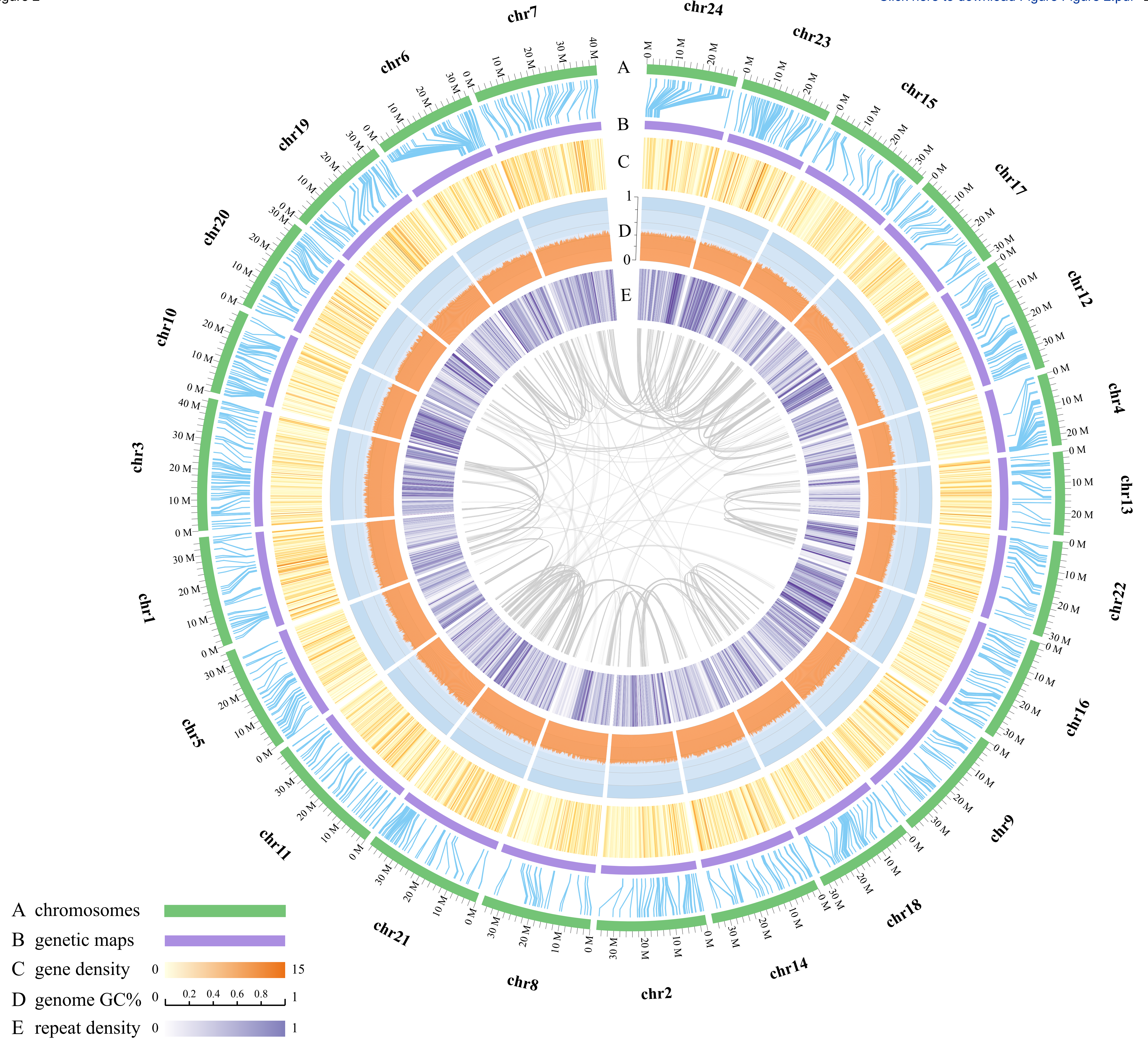

Figure 3

Click here to download Figure Figure 3.pdf

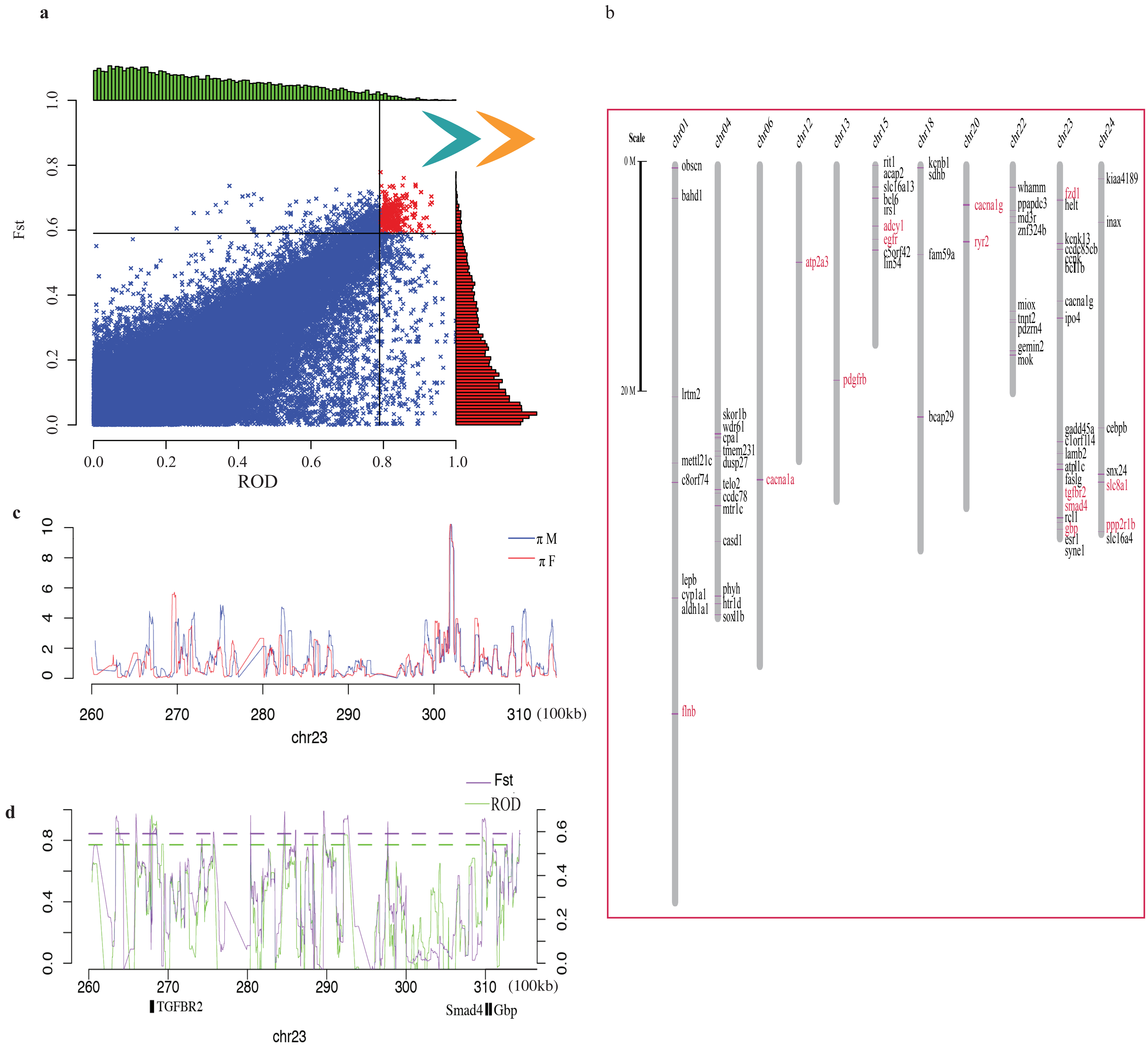

Figure 4

Click here to download Figure Figure 4.pdf

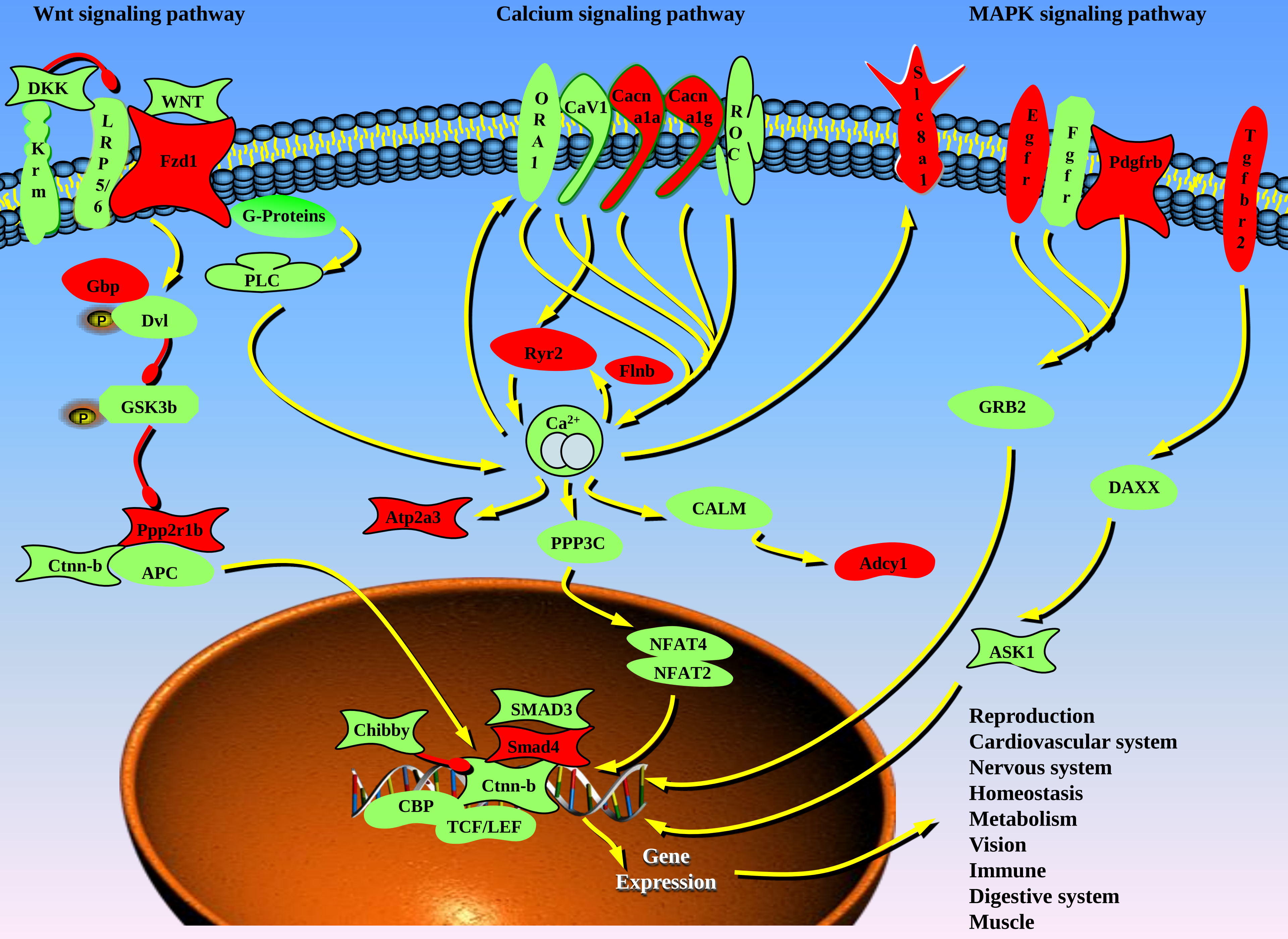

Figure 5 Click here to download Figure Figure 5.pdf

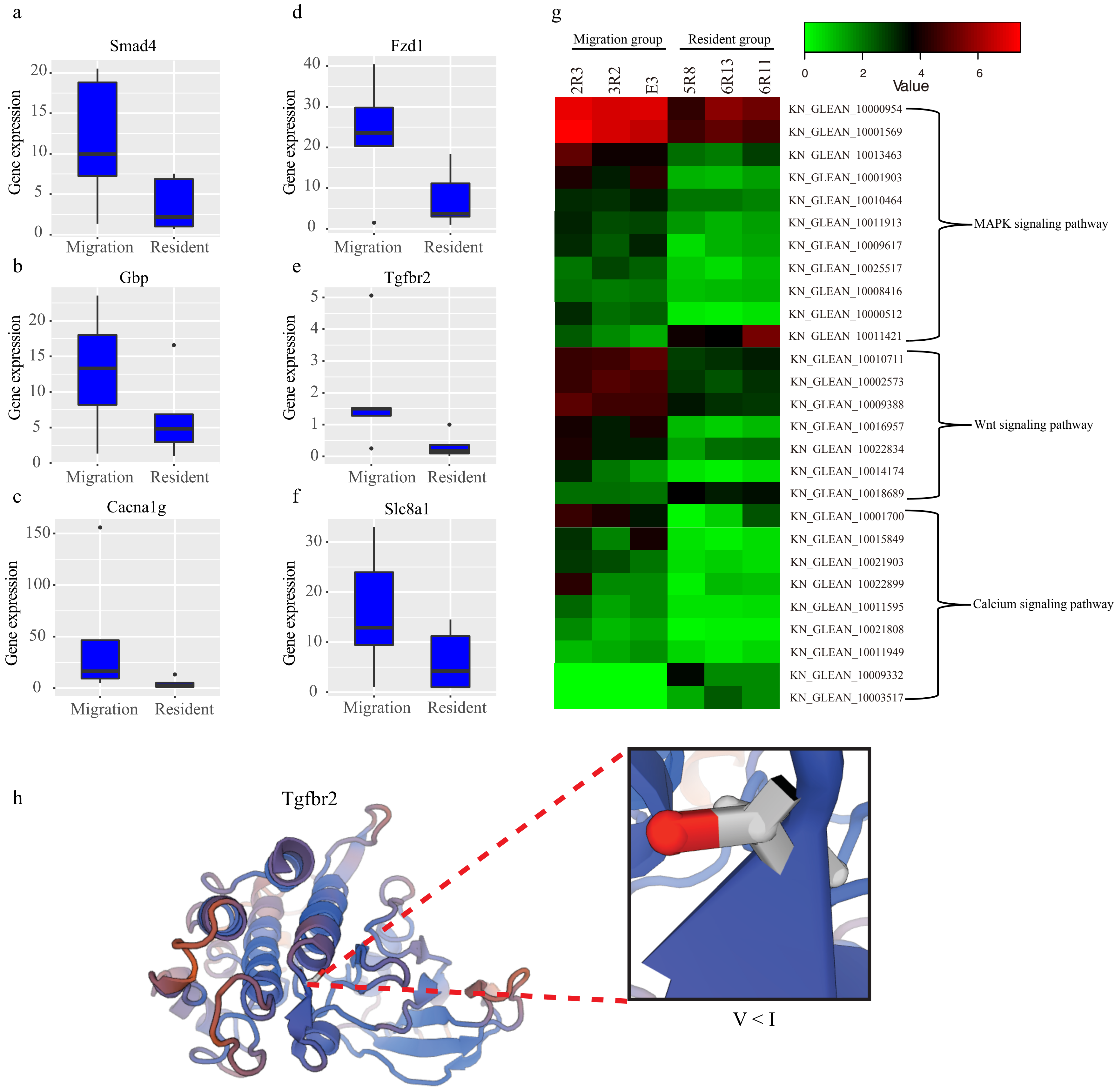

Click here to access/download
Supplementary Material
Supplementary materials.docx

Supplementary Table 10

Click here to access/download
Supplementary Material
Supplementary Table 10.xlsx

Supplementary Table 12

Click here to access/download
Supplementary Material
Supplementary Table 12.xlsx

Supplementary Table 13

Click here to access/download
Supplementary Material
Supplementary Table 13.xlsx
