# Supplementary material for: Genome and population sequencing of a chromosome-level genome assembly of the Chinese tapertail anchovy (Coilia nasus) provides novel insights into migratory adaptation
Source: Gigascience. 2020 Jan 2;9(1):giz157. doi: 10.1093/gigascience/giz157 (PMC6939831; doi:10.1093/gigascience/giz157)
Supplement: giz157_GIGA-D-19-00179_Revision_1 [file giz157_giga-d-19-00179_revision_1.pdf]

GigaScience

Genome and population sequencing of a chromosome-level genome assembly of Chinese tapertail anchovy (Coilia nasus) provides novel insights into migratory adaptation

--Manuscript Draft--

**Manuscript Number:**	GIGA-D-19-00179R1
**Full Title:**	Genome and population sequencing of a chromosome-level genome assembly of Chinese tapertail anchovy (Coilia nasus) provides novel insights into migratory adaptation
**Article Type:**	Research
**Funding Information:**	the National Natural Science Foundation of China (31672643) — Dr Gangchun xu
**Abstract:**	Background: Seasonal migration is one of the most spectacular events in nature; however, detailed mechanisms related to this interesting phenomenon have not been investigated in detail. Chinese tapertail anchovy, Coilia nasus, is a valuable migratory fish of high economic importance and special migratory dimorphism (with certain individuals as non-migratory residents). Results: In this study, an 870.0 Mb high-quality genome was assembled by the combination of Illumina and PacBio sequencing. 812.1 Mb of scaffolds were linked to 24 chromosomes using a high-density genetic map from a family of 104 full siblings and their parents. In addition, population sequencing of 96 representative individuals from diverse areas along the putative migration path confirmed the involvement of 150 genes in migratory adaption. Based on integrative genomic and transcriptomic analyses, we determined that three Ca2+-related pathways are critical for the promotion of migratory adaption. A large number of molecular markers were also identified, which distinguished migratory individuals and non-migratory freshwater residents. Conclusions: We assembled a chromosome-level genome for the Chinese tapertail anchovy. The genome provided a valuable genetic resource for understanding migratory adaption and population genetics, and will benefit the aquaculture and management of this economically important fish.
**Corresponding Author:**	Qiong Shi, PhD<br>BGI<br>Shenzhen, CHINA
**Corresponding Author Secondary Information:**	
**Corresponding Author's Institution:**	BGI
**Corresponding Author's Secondary Institution:**	
**First Author:**	Qiong Shi, PhD
**First Author Secondary Information:**	
**Order of Authors:**	Qiong Shi, PhD
	Gangchun xu
	Chao Bian
	Zhijuan Nie
	Yuyu Wang
	Dongpo Xu
	Xinxin You
	Hongbo Liu
	Jiancao Gao

Powered by Editorial Manager® and ProduXion Manager® from Aries Systems Corporation

	Changyou Song
	Kai Liu
	Jian Yang
	Quanjie Li
	Nailin Shao
	Yanbing Zhuang
	Dian Fang
	Tao Jiang
	Yunyun Lv
	Yu Huang
	Ruobo Gu
	Junmin Xu
	Wei Ge
	Pao Xu
**Order of Authors Secondary Information:**	
**Response to Reviewers:**	Reviewer #1:<br>## General comments ##<br>The authors have sequenced and assembled the Chinese tapertail anchovy (Coilia nasus). The Chinese tapertail anchovy is intriguing because it has both migratory and non-migratory populations, facilitating investigation into the genetic basis for migration. By sequencing 96 individuals (85 migratory and 11 non-migratory), the authors find 150 genes that have vastly different alleles between the migratory and non-migratory ecotypes.<br>There are multiple instances where the language is unclear or wrong. I would strongly advice going through the text carefully to find these places, maybe by a native English-speaking person. I have mentioned some places, but I would like to focus on the science and not the language.<br>Answer: Thanks for your advice. Yes, with kind assistance from the editor and ISE (a professional English editing company), we made a thorough revision of our manuscript, especially paid much attention to those places where you mentioned for changes.<br><br>## Specific comments ##<br>Title: I prefer "population sequencing" rather than "resequencing", but that is a personal preference and not a recommendation for a change.<br>Answer: Thanks for your advice. Yes, we changed “resequencing” to “population sequencing”.<br><br>Abstract: The 'Results' part is a bit heavy to read. You could spilt the first to sentences to get a better flow.<br>Answer: Thanks for your advice. Yes, we split this sentence into two on lines 35-38.<br><br>Line 60: "of" is not necessary.<br>Line 63: "molecular mechanisms have still been unknown" should be "molecular mechanisms are still unknown".<br>Answer: Thanks for your comments. Yes, we made corresponding changes on lines 58 and 61.<br><br>Line 69: Does the cited article actually discuss the fish' "nutritional content and delicate flavor"? Anyhow, I am uncertain of the validity of having this kind of statements in a scientific paper. People would not buy food that tastes bad, and over time, would not buy food without enough nutrition.<br>Answer: Thanks for your comments. We removed this sentence and the cited article on line 67.<br><br>Line 76: I don't think the approximation symbol is used as this usually.

Answer: We changed ‘3~4 more months’ to ‘3 to 4 months’ on lines 73-74.

Line 76-77: Could be written better.
Answer: Yes, it is done on lines 73-75.

Line 78: "always resident" and "their whole lifetime" means the same.
Answer: We removed ‘always’ in this sentence on line 75.

Line 87: RAD actually used for creating the assembly? Rewrite, the important part is that you created a linkage map and used that, not the RAD itself.
Answer: Sorry for the misleading description. According to your advice, we rewrote this sentence as follows on lines 83-86.
Thus, in this study, we firstly produced the chromosome-level genome assembly of C. nasus, on basis of the genetic linkage map constructed with the digest restriction-site associated DNA (RAD) sequencing.

Line 90: What does "high-evidence for examination of the detailed molecular" mean?
Answer: Thanks for your question. We revised part of this sentence as follows on lines 87-89: we identified numerous SNPs (single nucleotide polymorphisms) to detect the molecular clues for adaptive mechanisms between the migratory and freshwater residential groups.

Line 92-93: Could be written better.
Answer: Yes, it is done on lines 88-90. We rewrote it as follows.
The identified candidate genes for migratory adaptation will provide valuable resources for genetic research on fish migration.

Line 104: You could specify that this was using the actinopterygii dataset.
Answer: Thanks for your advice. Yes, we add “with actinopterygii_odb9 orthologues” in this sentence on line 104.

Line 105: "A BUSCO evaluation of our assembly was 90.1%," does not make sense. Please rewrite.
Answer: Thanks for your question. Yes, we revised this sentence as follows on lines 105-107.
The assessment result of our assembly was 90.1%, where C=87.1% [D=4.6%], F=3.0%, M=9.9%, and n=4584 (C: complete [D: duplicated], F: fragmented, M: missed, n: number of genes), thereby suggesting a high level of completeness for the C. nasus assembly

Line 125-126: Is this normal? Seems a bit low.
Answer: Yes, a mapping ratio over 60% is normal in a population sequencing.

Lines 128-130: You cannot call 25 million SNPs for 25 Mb SNPs. You could say 25 M SNPs however.
Answer: Yes, you are right. We changed ‘Mb’ to ‘M’ on lines 129-133.

Line 134: The "SNP set" is all 39 M SNPs? Or is it a reduced set? How many SNPs in that case?
Answer: Yes, the tree was constructed using the entire 39 M SNP set.

Line 136: Why so few non-migratory? Would it not be better comparing two groups of equal size than this large difference?
Answer: Thanks for your comments. Theoretically, it would be more accurate to collect various individuals with the same quantity. However, the freshwater residents were extremely rare and difficult for collection. In the present study, only 11 non-migratory samples were available after validation. However, we will try to collect more samples in different water areas.

Lines 158-159: "suggesting that the migratory adaptation may require a preference for selected genes in adjacent locations". What does this mean? How can you tell if this is a real phenomenon?
Answer: Sorry for this misleading description. We changed it to the following sentence

on lines 155-156.
These genes had potentially undergone independent selection for involvement in migratory adaptation.

Line 171: I do not think you can say that "genes were clustered in terms of [..] physical (chromosomal) positions" if your example in line 162 is typical. 3 genes spread across 5 Mbp is not a lot. Further, how can you say that the genes are "clustered [..] in [..] physiological functions"? Would not this be expected? A migratory behavior would need some triggers, which some of the GO pathways might be, and would need some neurological connection. I do not find this surprising. Is as expected, I would say.
Answer: Thanks for your comments. We changed ‘the migratory adaptation-related genes were clustered in terms of both physical (chromosomal) positions and physiological function ‘to ‘these gene terms could be related to the migratory adaptation’ on line 173.

Section "Differentially expressed genes (DEGs) in the Ca2+-related pathways for the migrator group": If these genes are always more expressed in migratory fish than non-migratory fish, how can you tell if they are actually connected to migration? Would not the expectation be that something creates a cascade and up-regulates a bunch of genes, which then make the fish do the migration. If the genes are up-regulated the whole time, how would that contribution to the migration? When were the analysis done? I see from the section called "Genetic adaptation to complex environments during migration" that you say that these might be needed for the migratory fish to handle freshwater, which seems logical.
Answer: Thanks for your advice. It is indeed that many genes have higher expression levels in migratory fishes than in non-migratory individuals, which could be difficult to identify the real relationships for migration. However, in this DEG term, we mainly focused on the genes with non-synonymous SNPs. We thus speculated that these genomic variants potentially lead to the changes of transcription, and further influence the migration ability.

Line 303: Has the SOAPdenovo assembly been used for anything? That is not clear to me.

Answer: Thanks for your question. In fact, the SOAPdenovo assembly was used for SNP calling of population sequencing. Please find more details on lines 390-391.

Line 314: Why did you generate a Platanus assembly if you had a SOAPdenovo assembly already?
Answer: Thanks for the question. In fact, we independently run SOAPdenovo and Platanus. However, the Platanus data were more suitable for the combined assembling of Illumina and PacBio sequencing reads.

Line 332: Which assembly was annotated?
Answer: Both assemblies were used for genome annotation, independently. However, the results were combined at the end of annotation.

Line 408: Why have you used both assemblies in your analysis? You should in that case report the statistics and BUSCO scores for the SOAPdenovo assembly in the results, so that we as readers can assess its quality better.
Answer: Thanks for your advice. We added the BUSCO results of our SOAPdenovo assembly. Please find the details of two BUSCO scores on lines 106-108 and 316-318, respectively.

Section "Availability of supporting data": I could not find any of the genome assemblies on the two provided project IDs. Please make sure that they are released.
Answer: Thanks for your comments. Yes, the SOAPdenovo assembly was released under the project ID PRJNA421870, and the Pacbio assembly was uploaded to Gigascience FTP. Please find more details on lines 485.

Figure 2: Why do you have that particular order of the chromosomes? Why not 1,2,3,4 etc? What is the naming of the chromosomes based on? It is common to call the largest sequence for chromosome 1, second largest for chromosome 2 and so on. This does not seem to be the case here.

Powered by Editorial Manager® and ProduXion Manager® from Aries Systems Corporation

Answer: Thanks for your good question. In our present study, however, we did not use the chromosome size as the key factor to name the chromosomes. In fact, we named these chromosomes based on our newly constructed genetic linkage map. For example, the chromosome assembled from LG1 was named as Chr1. Thus, the size of Chr1 is not the largest. Please find more details of each chromosome in Supplementary Table 6.

Supporting data: Please provide the SOAPdenovo assembly here also.

Answer: Thanks for your advice. Yes, we added a brief table, Supplementary Table 7, to provide statistics of the SOAPdenovo assembly.

Reviewer #2:
The article reports the chromosome-wide genome assembly of the Chinese tapertail anchovy, and its respective use for population genomics studies. The genomic tools and data generated are of high relevance, as demonstrated here by its usefulness to address a biological question, the evolutionary aspects of migratory dimorphism.

The major weakness of the manuscript is the overall description inaccuracy in the methods and results sections, as listed below:
* Results presented without any respective methodology description, for example synteny blocks in Figure 2, enrichment pathway analysis for 150 candidate genes, BUSCO assessment (guessed the actinopterygii dataset was used by the number of gene models), and DEGs Heatmap in Figure 5g.

Answer: Thanks for your advice. Yes, we added more detailed method descriptions for the sections of synteny blocks (lines 406-409), enrichment pathway analysis (lines 432-434 & 448-449), BUSCO analysis (lines 102-103) and DEGs Heatmap (lines 455-458).

* Regarding population structure the results presented are solely a phylogenetic tree figure without presenting consolidated statistical values. Additionally confirmed by otoliths microstructure results, but no value is presented (eg . correlation). In the respective methods part is mentioned "PLINK was applied to calculate the genetic distance between individuals,..". What was the actual analysis procedure done with PLINK?

Answer: Thanks for your questions and advice. In fact, the phylogenetic tree were constructed by SNPs, thereby it does not have any statistical numbers. For the otoliths microstructure results in Figure 1, we demonstrated the scale bar of Sr concentrate. Based on Fig 1c, we could determine that the Sr concentrate in freshwater resident fishes was around 0.2%,whlie in migratory fishes it was around 0.4%. These differences could distinguish the two groups of fishes. The PLINK with parameters "--distance 1-ibs flat-missing" was used to calculate the genetic distances among individuals. Please find more details of the corresponding procedure on lines 419-424.

* Results mixed in the methods section (eg. lines 280-282; line 286-287; 302; 315-316; 319-320; 326-327; 329-331; and 399-406).

Answer: Sorry for the mixture of methods and results. We removed these sentences from the Methods section.

* Resequencing methodology for the population genomic study mentioned pooled samples but supplementary file sequencing libraries are for each 96 individual samples.

Answer: Sorry for the mistake. We changed it to "Population genome sequencing library (average insert size of about 350 bp) of each individual was independently constructed for DNAs from the 96 individuals" on lines 296-298.

* Methodologies in general are omissive regarding details necessary for reproducibility purpose or to understand the results reported, such as DNA amount used for libraries purposes, sampled tissues for qPCR, t-test assumption of data normality and equal variance, lack of the used software naming (line 306: "total reads were mapped onto the contigs") and/or respective parameters (if default was used state it).

Answer: Thanks for your advice. Yes, we added the detailed DNA amount extracted from denovo (about 90 μg) and population sequencing individuals. Please find more details on lines 282 and 294.
The sampled tissue for qRT-PCR is muscle (lines 450-452). For the t-test assumption, we changed this sentence to be ‘Normal distribution and homogeneity of variance of data was tested with the Shapiro-Wilk and Levene tests (α = 0.05), respectively. Then differences in the mRNA levels were compared by the students' t-test using IBM SPSS Statistics 22.0 (IBM Inc., Chicago, IL, USA). P values of < 0.05 were considered statistically significant’. Please find more details on lines 459-469.
The sentence “total reads were mapped onto the contigs” was changed to “total reads were mapped onto the contigs by the third step of SOAPdenovo with default parameters” on lines 310-313.

* One example of methodology description not being accurate is the RNAseq expression analysis (line431-440). No description to the raw data editing and quality control filtering procedure. The mapping procedure solely names the HISAT2 without any parameters used specified. The usage of Cufflinks 2.2.1 suite of tools description is confusing; solely mentioning the Cufflinks program itself (which assembles the mapped reads into transcriptomes) and Cuffdiff to identify DEGs. No parameters of the programs are described, and no reference to the intermediate softwares in the workflow is done, such as Cuffcompare, Cuffmerge, CuffQuant and downstream the Cuffnorm. Presuming the usage of Cuffnorm to normalize the expression values across samples required for the heatmap presented in Figure 5g.

Answer: Thanks for your advice and comments. We added information of the detailed filter step of transcriptome reads on lines 446-449 as follows:
Raw data produced from the sequencing platform were filtered by removing reads contaminated with adaptors, more than 10% of N bases and more than 50% of low-quality bases (base quality score ≤ 10).

We also added the detailed parameters “--phred33 --sensitive --no-discordant --no-mixed -I 1 -X 1000” for HISAT2 on lines 450-451.
More details about ‘The Cuffdiff in Cufflink package with parameters “-FDR 0.05 --geometric-norm TRUE –c 10” was added ‘to identified the significantly differentially expressed genes (DEGs)’ on lines 453-455.
For the Heatmap, edgeR software was used to normalize the expression values and transform the values to log2 values. Please find more details on lines 445-448.

* I'm not against the use of personal pronouns in scientific manuscript writing, but the extensive use of "We" in the results and in methods sections should be avoided, because doing so removes objectivity required in these sections.

Answer: Thanks for your advice. The “We” manner in our manuscript was reduced in the methods section.

Without having addressed these main points listed above, is difficult to evaluate the fullness and implications of the results presented. Therefore, any other revision queries are not relevant until methods & results sections are revised. As exception, just to raise one point regarding one particular analysis and respective conclusion. This is about the identification of the 150 candidate genes related to the migratory adaptation, solely based on the colocalization with selective sweep regions. These genomic signatures of divergence don't necessarily have a cis effect on the neighbouring gene, as many have a functional trans effect on other loci further away in the linearity of the DNA. If you wanted to focus on the functional gene unit, why didn't you complemented it with the Non-synonymous/Synonymous mutation ratio (Ka/Ks) analysis of those genes, since you identified those coding region located SNPs?

Answer: Thanks for your advice. However, the Ka/Ks analysis is more available for the

	gene comparisons among diverse species, although there are some but too little diverse nuclear sites in coding regions for a Ka/Ks analysis in the same species. Fst and selective sweeping analysis were widely used in population sequencing or resequencing studies. We mainly focused on those genes with non-synonymous SNPs. We thus speculated that these genomic variants potentially led to changes of expression values, and further influenced the migration ability.<br><br>To summarise it, the genomic tools, the data generated and the biological question addressed are of high relevance, but hampered by the manuscript methods and results reporting.<br><br>Answer: Thanks for your nice advice. We added more detailed method descriptions according to your suggestions in the revised manuscript.
**Additional Information:**	
**Question**	**Response**
Are you submitting this manuscript to a special series or article collection?	No
**Experimental design and statistics**<br><br>Full details of the experimental design and statistical methods used should be given in the Methods section, as detailed in our Minimum Standards Reporting Checklist. Information essential to interpreting the data presented should be made available in the figure legends.<br><br>Have you included all the information requested in your manuscript?	Yes
**Resources**<br><br>A description of all resources used, including antibodies, cell lines, animals and software tools, with enough information to allow them to be uniquely identified, should be included in the Methods section. Authors are strongly encouraged to cite Research Resource Identifiers (RRIDs) for antibodies, model organisms and tools, where possible.<br><br>Have you included the information requested as detailed in our Minimum Standards Reporting Checklist?	Yes
**Availability of data and materials**	Yes

All datasets and code on which the conclusions of the paper rely must be either included in your submission or deposited in publicly available repositories (where available and ethically appropriate), referencing such data using a unique identifier in the references and in the "Availability of Data and Materials" section of your manuscript.<br><br>Have you have met the above requirement as detailed in our Minimum Standards Reporting Checklist?	

Powered by Editorial Manager® and ProduXion Manager® from Aries Systems Corporation

Manuscript

Click here to access/download;Manuscript;Manuscript 9-29 with endnote.docx

Click here to view linked References

Genome and population sequencing of a chromosome-level genome assembly of Chinese tapertail anchovy (*Coilia nasus*) provides novel insights into migratory adaptation

Gangchun Xu[1,2,†], Chao Bian[3,4,†], Zhijuan Nie[2†], Jia Li[3†], Yuyu Wang[2], Dongpo Xu[2], Xinxin You[3,5], Hongbo Liu[2], Jiancao Gao[2], Hongxia Li[2], Kai Liu[2], Jian Yang[2], Quanjie Li[2], Nailin Shao[2], Yanbing Zhuang[2], Dian Fang[2], Tao Jiang[2], Yunyun Lv[3,5], Yu Huang[3,5], Ruobo Gu[2], Junmin Xu[3], Wei Ge[4], Qiong Shi[3,5,*], Pao Xu[1,2,*]

[1]Wuxi Fisheries College, Nanjing Agricultural University, Wuxi, Jiangsu 214081, China.

[2]Key Laboratory of Freshwater Fisheries and Germplasm Resources Utilization, Ministry of Agriculture, Freshwater Fisheries Research Center, Chinese Academy of Fishery Sciences, Wuxi, Jiangsu 214081, China.

[3]Shenzhen Key Lab of Marine Genomics, Guangdong Provincial Key Lab of Molecular Breeding in Marine Economic Animals, BGI Academy of Marine Sciences, BGI Marine, BGI, Shenzhen, Guangdong 518083, China.

[4]Centre of Reproduction, Development and Aging, Faculty of Health Sciences, University of Macau, Taipa, Macau, China.

[5]BGI Education Center, University of Chinese Academy of Sciences, Shenzhen, Guangdong 518083, China.

*Correspondence address. Pao Xu, Freshwater Fisheries Research Center, Chinese Academy of Fishery Sciences, Wuxi, Jiangsu 214081, China. Tel: +86-138 0619 0669; E-mail: xup@ffrc.cn; Qiong Shi, BGI Academy of Marine Sciences, BGI Marine, BGI, Shenzhen, Guangdong 518083, China. Tel: +86-185 6627 9826; E-mail: shiqiong@genomics.cn

†Contributed equally to this work.

Abstract

Background: Seasonal migration is one of the most spectacular events in nature; however, detailed mechanisms related to this interesting phenomenon have not been investigated in detail. Chinese tapertail anchovy, *Coilia nasus*, is a valuable migratory fish of high economic importance and special migratory dimorphism (with certain individuals as non-migratory residents). **Results:** In this study, an 870.0 Mb high-quality genome was assembled by the combination of Illumina and PacBio sequencing. 812.1 Mb of scaffolds were linked to 24 chromosomes using a high-density genetic map from a family of 104 full siblings and their parents. In addition, population sequencing of 96 representative individuals from diverse areas along the putative migration path confirmed the involvement of 150 genes in migratory adaption. Based on integrative genomic and transcriptomic analyses, we determined that three Ca^{2+}-related pathways are critical for the promotion of migratory adaption. A large number of molecular markers were also identified, which distinguished migratory individuals and non-migratory freshwater residents.

Conclusions: We assembled a chromosome-level genome for the Chinese tapertail anchovy. The genome provided a valuable genetic resource for understanding migratory adaption and population genetics, and will benefit the aquaculture and management of this economically important fish.

Keywords: Chinese tapertail anchovy (*Coilia nasus*); genome and population sequencing; genome assembly; migratory dimorphism and adaptation

Introduction

Migration is one of the most spectacular events in nature. Every year, billions of animals take part in a seasonal movement to find food or mates, avoid predators, or escape a severe living environment. Hence, seasonal migration can influence the distribution of animals across space and time. Determining related mechanisms of migratory adaptation is critical for understanding evolutionary processes, and for facilitating management of stocks and conservation of endangered species. Many studies have aimed to understand this interesting phenomenon [1, 2]; however, the detailed molecular mechanisms are still unknown.

Chinese tapertail anchovy, *Coilia nasus* (Figure 1a), is a valuable migratory fish with high economic importance in China and can be classified into two groups according to living habitats. One is the routine migratory group, with a wide distribution in sea areas close to Korea, China and Japan. In China, this fish is mainly captured from the Yellow Sea, East China Sea, and Yangtze River [3]. Similar to

Pacific salmon (*Oncorhynchus* spp.) [2], *C. nasus* adults are known to migrate from February to April each year anadromously to the Yangtze River before their final gonadal maturation in order to spawn in the middle and lower reaches of the Yangtze River (more details in Figure 1b). This represents a long distance of thousands of kilometers between the open ocean (for growth) and the natal stream (for reproduction) [4]. After spawning, adult fish migrate to the sea. The juveniles remain in fresh water for 3 to 4 months until they acquire the ability to tolerate sea water, they then follow the path of their parents and migrate to the sea [5]. The other group has been reported to be resident in some freshwater lakes during their entire lifetime [6]. This phenomenon, known as partial migration or migratory dimorphism [1], provides an opportunity to obtain insights into migratory adaptation.

Many studies have investigated this process but most simply described the patterns of migratory dimorphism [7] or its occurrence in a given population [8]. These data provided little information on the related genetic variations from the perspective of the whole genome. In addition, the detailed mechanisms related to migratory dimorphism in fish are disputed and poorly understood. Thus, in this study, we first produced the chromosome-level genome assembly of *C. nasus*, based on the genetic linkage map constructed with the digest restriction-site associated DNA (RAD) sequencing [9]. After population genome sequencing of 96 individuals from diverse areas along the putative migration path (Figure 1b and Table 1), we identified numerous SNPs (single nucleotide polymorphisms) to detect molecular clues for adaptive mechanisms between the migratory and freshwater resident groups. The

identified candidate genes for migratory adaptation will provide valuable resources for genetic research on fish migration.

Results

Sequencing and assembly of a chromosome-level genome

We sequenced approximately 277.9 gigabases (Gb) of short reads (100–150 bp) using an Illumina Hiseq 2500 platform (Illumina, San Diego, CA, USA) and 68.6 Gb of long reads (an average of 14,743 bp) from a PacBio RSII platform (Pacific Biosciences, Menlo Park, CA, USA) (see more details in Supplementary Table 1). After removal of low-quality raw reads, we assembled a high-quality genome with a scaffold N50 and a contig N50 of 2.1 Mb and 1.6 Mb, respectively. Our genome assembly spanned about 870.0 Mb, which is consistent with the predicted genome size of 857.5 Mb based on a *K*-mer analysis (Supplementary Figure 1 and Supplementary Table 2)[10]. The BUSCO (University of Geneva Medical School and Swiss Institute of Bioinformatics, Geneva, Switzerland; version 3.03, RRID:SCR_015008) [11] with actinopterygii_odb9 orthologues was used to evaluate the completeness of our assembly. The assessment result of our assembly was 90.1%, where C=87.1% [D=4.6%], F=3.0%, M=9.9%, and n=4584 (C: complete [D: duplicated], F: fragmented, M: missed, n: number of genes), thereby suggesting a high level of completeness for the *C. nasus* assembly.

In addition, a high density linkage map of *C. nasus* based on the RAD sequencing of a family of 104 full siblings with their parent pairs was constructed. Subsequently,

we localized a total of 15,300 high-quality SNPs into 24 linkage groups with a genetic distance up to 7,651.0 cM. Finally, 93.3% of the assembled genome sequences (812.1 Mb/870.0 Mb) were allocated to the 24 putative pairs of chromosomes (Figure 2, Supplementary Figure 2).

Repeat sequences were predicted to comprise approximately 31.1% of the *C. nasus* genome (Supplementary Table 3). These repeat sequences were classified into several representative types, and it was observed that Simple and hAT repeat sequences were the most abundant types (accounting for 5.75% and 5.65%, respectively) in the genome assembly (Supplementary Table 3). We also annotated 20,837 genes with an average length of 16.8 kb (Supplementary Table 4), of which 20,300 genes have functional assignments with public databases (Supplementary Table 5). Details of the chromosomal map and markers (Supplementary Table 6), density of genes, GC content, and repeat sequences are summarized in Figure 2.

Population genome sequencing and identification of variations

Whole-genome population sequencing generated approximately 4.5 billion of 125-bp paired-end reads (i.e., 684.0 Gb of raw data). The mapping ratio for each sample ranged from 64.0% to 71.0%, and the average mapping depth was determined to be approximately 10 folds (Supplementary Table 8). In total, 39.4 million (M) high-confidence SNPs were called, and they were then annotated based on their positions in the chromosomes. Most of the SNPs (25.3 M, 64.1%) were identified in intergenic regions, while 1.31 M of the SNPs (33.3%) were distributed in intron regions, and only 1.0 M of the SNPs (2.6%) were distributed in coding regions.

Among the SNPs within coding regions, we identified 472,322 synonymous SNPs and 545,212 non-synonymous SNPs (Supplementary Table 9).

In order to identify the detailed divergence at the genome level among the 96 examined individuals, we constructed a phylogenetic tree based on the entire SNP set. Interestingly, the tree demonstrated that these individuals could be clearly divided into two groups, in which 11 were freshwater residents and 85 were migratory individuals (Figure 1c). For confirmation of this grouping, we also employed electron probe microanalysis [12] to check whether these fish were migratory. As we reported previously, the migratory group can be discriminated based on the Sr (strontium) and Ca (calcium) signatures in otoliths [12-14]. As different environmental conditions can lead to variations in the Sr contents and Sr:Ca ratios in otoliths, we employed blue (Sr:Ca ratio ≤3.0), green or yellow (Sr:Ca ratio = 3.0–7.0), and red (Sr:Ca ratio >7.0) regions in Figure 1d and Supplementary Figure 3 to represent fresh water, brackish water, and sea water patterns, respectively [3, 15]. It appears that our SNP set could clearly distinguish the divergence between these freshwater residents and migratory individuals (Figure 1c), which was validated by the electron probe microanalysis (Figure 1d). Therefore, this SNP set (detailed in Supplementary Table 9) can be used as genetic markers for a complement of the common performance of otolith microstructures.

Identification of 150 genes related to migratory adaption

We screened 661 windows with the top 5% Fst (fixation index for diversity differentiation) and ROD (reduction of diversity) values, where 150 functional genes

were identified (Figure 3a, Supplementary Table 10). These genes had potentially undergone independent selection for involvement in migratory adaptation. Interestingly, some of the selected genes were physically clustered in the assembled genome. For example, among the 150 migratory adaptation-related genes, 90 (60.0%) were distributed on six chromosomes (Figure 3b, Supplementary Table 10). In particular, chromosomes 23, 4, and 15 were the three main chromosomes related to migratory adaptation, and 19 genes were localized on the chromosome 23 (Figure 3b). Moreover, genes with selective sweep signals were identified based on $\pi_{migration}/\pi_{freshwater}$ (Figure 3c), Fst, and ROD (Figure 3d) using a 5-kb sliding window between the 26th Mb and 31st Mb. Three migration-related genes, including *Tgfbr2*, *Smad4*, and *Gbp* were localized within this region (Figure 3d).

In order to further clarify the functions of these 150 genes, we performed GO (gene ontology) and pathway enrichment. These genes were predicted to participate in several important functions, such as “substrate-specific transporter activity” (GO:0022892), “ion transmembrane transporter activity” (GO:0015075), “cation channel activity” (GO:0005261), “potassium channel activity” (GO:0005267), and “neuropeptide hormone activity” (GO:0005184) (Supplementary Table 11). They were significantly enriched in 11 pathways (Supplementary Table 12), which suggested that these gene terms could be related to migratory adaptation. Three pathways related to Ca^{2+} metabolism were enriched, including the calcium signaling pathway, MAPK signaling pathway, and Wnt signaling pathway (Figure 4). These data indicate that Ca^{2+}-related pathways may play key roles in adaptation to

migration.

Differentially expressed genes (DEGs) in the Ca^{2+}-related pathways in the migratory group

We analyzed the variable sites in 14 genes within the three Ca^{2+}-related pathways (red in Figure 4). In total, 277 non-synonymous SNPs were distributed in the coding sequence regions, and their allele frequencies were significantly different in the two fish groups (Supplementary Table 13). In order to validate whether the DNA variations affected gene transcription, we quantified the mRNA changes for several randomly selected genes (Figure 5a–d) by real-time quantitative RT-PCR. Our results demonstrated that *Smad4* (Figure 5a), *Gbp* (Figure 5b), *Fzd1* (Figure 5d), *Tgfbr2* (Figures 3b, 3c, and 5e), and *Slc8a1* (Figure 5f) were transcribed more in the liver of the migratory group than that of freshwater residents. Moreover, *Cacnalg* had a higher transcription level (Figure 5c) in the heart of the migratory group than the freshwater residents. We also compared the transcription values in brain tissues of the migratory and resident groups, and 648 DEGs were identified (Supplementary Table 14). In particular, 27 genes were from the three Ca^{2+}-related pathways, and most of the genes (23) had higher transcription values in the migratory group than the freshwater residents (Figure 5g). It seems that the migratory group maintained gene transcription of the three Ca^{2+}-related pathways at a high level for migratory adaptation. In addition, the DNA variations may have caused changes in the tertiary structure of proteins to allow variable protein functions. For example, the 298V site in Tgfbr2 located in the protein kinase domain (Figure 5h), catalyzes transfer of the gamma phosphate from

nucleotide triphosphates to one or more amino acid residues in a protein substrate side chain, resulting in a conformational change to potentially affect the corresponding protein function [16, 17].

Discussion

Fish migration is an interesting natural phenomenon. The migratory adaptation mechanisms in fish have been studied from various perspectives, such as ecology, physiology, genetics, and morphology [1, 2]. However, they have rarely been examined from a whole genome view. After analyzing the genome sequencing and population genome sequencing data, we identified 150 candidate genes embedded in the selected sweep regions that are potentially involved in migratory adaptation. It seems that the molecular mechanisms of migratory adaptation can be interpreted at the following three major levels: reproductive adaptation, long-distance migratory adaptation, and complex environmental adaption.

Genetic basis of reproductive adaptation

The main aim of migration is to spawn to ensure a wide distribution of species. Thus, migratory adaptation should first involve endocrine and reproductive adaptation. In our previous study [18] , we reported that unsaturated fatty acid metabolism and steroid hormone biosynthesis are involved in the regulation of ovarian development in *C. nasus*. Of the 150 candidate genes identified in the present study, *Acoxl* is known to play an important role in the biosynthesis of unsaturated fatty acids (Supplementary Table 10 and 11). In addition, four genes from oocyte meiosis and maturation

pathways were included in this list of 150 genes (Supplementary Table 10 and 11), which were also potentially involved in reproductive adaptation. Several genes with selective sweep signals in the migratory group, including *Fzd1, Ppp2r1b, Cacna1a* and *Smad4*, were also confirmed to affect the reproductive capacity of females and males in previous knockout experiments [19-24]. Hence, they are expected to play important roles in reproductive adaptation in our migratory group.

Positive selection of candidate genes for long-distance migratory adaptation

The *C. nasus* migratory group must undergo long-term countercurrent migration, which requires high athletic capacity. Some selective sweeping regions in the migratory group covered several important genes, such as *Atp2a3*, *Flnb* and *Acna1g*, that are associated with cardiovascular, hematopoietic and muscle functions [25-27]; these genes could participate in adaptation to long-distance migration. Moreover, genes related to nervous system development and spatial recognition, such as *Egfr*, *Adcy1*, *Flnb, Acna1g* and *Tgfbr2,* also harbored selective sweep signals, suggesting that evolution of these genes could be important for the orientation recognition of open water in the migratory group [28, 29]. In addition, fish rarely feed during migration [30, 31]. Several digestion- and metabolism-related genes (including *Tgfbr2, Smad4*, *Ryr2*, *Cacna1a*, *Pdgfrb*, and *Slc8a1*) have undergone selective sweeping, which may have contributed to the highly efficient digestion and metabolism in the migratory group [24].

Genetic adaptation to complex environments during migration

Salinity and osmotic pressure adaptations are essential for migration. It has been reported that the Ca^{2+} signaling pathway is important for regulation of osmotic pressure [32]. . The critical 14 genes (red in Figure 4) in the list of 150 DEGs with selective sweep signals were significantly enriched in the three central Ca^{2+}-related pathways (P<0.01; Supplementary Tables 12 and 13). We also observed that DNA-level variations elevated the transcription of genes in these three pathways to affect their functions (Figure 5). These three central Ca^{2+}-related pathways have a key role in cell proliferation and osmotic pressure regulation [33-35]. We also found that six genes with strong selective sweep signals were significantly enriched in GO terms of metal and calcium ion transport (P<0.05; Supplementary Table 11), which could also be related to salinity and osmotic pressure adaptation.

In addition, some genes (such as *Flnb*, *Tgfbr2*, *Pdgfrb*, and *Smad4*; Supplementary Table 13) related to renal function and homeostasis also underwent selective sweeping, suggesting their potential contribution to the alternative adaptation to salt water and freshwater [36]. Previous studies showed that the visual and olfactory systems were essential for migratory fish [37, 38]. Interestingly, some visual and olfactory related genes were also identified among the 150 candidate genes in the migratory group of *C. nasus* (Supplementary Table 11).

Conclusions

In summary, we performed whole genome sequencing of the Chinese tapertail anchovy (*C. nasus*) and constructed a high density genetic linkage map to generate a

high-quality chromosomal map. In total, 96 individuals were collected over a range of 618 km during reproductive migration for population genome sequencing. Based on these data and otolith X-ray electron microprobe validation, we determined 11 individuals as freshwater residents whereas the remaining individuals were migratory fish. Our high-quality reference genome and large amount of population genome sequencing data provide a good opportunity to examine the migration process, and reveal a more comprehensive image of *C. nasus* population genetics that will facilitate practical aquaculture and management of this economically important fish. Identification of 150 candidate genes with significant enrichment in three critical Ca^{2+}-related pathways supports the molecular mechanisms of migratory adaptation at the following three major levels: reproductive adaptation, long-distance migratory adaptation and complex environmental adaptation.

Materials and Methods

Sample collection and sequencing

A healthy female *C. nasus*, cultivated at our local base in Yixing city (Jiangsu Province, China) with a body weight of 167.0 g, was used for whole genome sequencing. Skeletal muscle was collected and immediately stored in liquid nitrogen. Genomic DNA (a total of approximately 90 μg) was extracted using Qiagen Genomic Tip100 (Qiagen, Hilden, Germany). The traditional whole-genome shotgun sequencing strategy was employed [39]. Three short-insert libraries (250, 500, and 800 bp) and four long-insert libraries (2, 5, 10, and 20 kb) were constructed according

to the manufacturer's instructions (Illumina, San Diego, CA, USA).

AMPure PB magnetic beads (Pacific Biosciences, Menlo Park, CA, USA) were utilized to concentrate the extracted high-quality genomic DNA for library construction with the SMRTbell template prep kit 2.1 (Pacific Biosciences). Sequencing was performed on a Pacific Bioscience (PacBio) Sequel platform.

Based on the putative migration path of *C. nasus*, 96 individuals were sampled from different localities in the Yellow Sea, Chongming, Nantong, Jingjiang, Anqing, Hukou, and Duchang (see more details in Figure 1b and Table 1). Genomic DNA (approximately 3 μg DNA from each individual) was isolated from skeletal muscle using Qiagen Genomic Tip100 (Qiagen, Hilden, Germany). The population genome sequencing library (average insert size of about 350 bp) of each individual was independently constructed for DNAs from the 96 individuals, and 2 × 150 bp paired-end reads were generated by an Illumina HiSeq2500 platform.

All animal experiments in this study were performed in accordance with the guidelines of the Animal Ethics Committee and were approved by the Institutional Review Board on Bioethics and Biosafety of BGI (No. 18134).

Estimation of genome size and assembly of the genome

The *C. nasus* genome size (G) was estimated by a *K*-mer analysis [10] according to the following formula: G = Kmer_num/Kmer_depth, where Kmer_num is the total number of reads and Kmer_depth represents the frequency of occurring more frequently than others.

SOAP*denovo*2 (version 2.04.4; RRID:SCR_014986) [40] with optimized

parameters (pregraph -K 27 -d 1; contig -M 1; scaff -F -b 1.5 -p 16) was employed to construct contigs and original scaffolds based on the sequenced reads. Subsequently, total reads were mapped onto the contigs by the third step of SOAPdenovo with default parameters for scaffolding according to the long-insert paired-end information, which led to linkage of contigs to scaffolds in a stepwise manner. Approximately 109.2 Gb of cleaned reads from the short-insert (250, 500, and 800 bp) libraries were then used to fill gaps in scaffolds with the GapCloser (v1.12-r6; RRID:SCR_015026; default parameters and -p set at 25). Finally, the first version of the genome assembly was generated. The BUSCO value achieved was 88.6%, where C = 86.8% [D = 4.5%], F = 1.8%, M = 11.4%, and n = 3023 (C: complete [D: duplicated], F: fragmented, M: missed, n: number of genes).

To improve the *de novo* assembly, 68.6 Gb of PacBio reads were also sequenced. Platanus (version 1.2.1, RRID:SCR_015531) [41] was used to generate a *de novo* assembly with a total of 1.0 Gb and a contig N50 of 764 bp using Illumina reads from the short-insert (250, 500, and 800 bp) libraries. Subsequently, all PacBio reads and the above assembled contigs were used for further assembly by utilizing the DBG2OLC pipeline (default version) [42] with the following parameters: LD10, MinLen 200, KmerCovTh 6, MinOverlap 80, AdaptiveTh 0.012, and RemoveChimera 1. A polishing step for this assembly was then performed using Illumina reads from the short-insert libraries. These reads were mapped onto the contigs using BWA-MEM (version 0.6.2, RRID:SCR_010910) [43]. Pilon (version 1.22, RRID:SCR_014731) [44] was also used to correct the assembly according to the

alignment. SSPACE (version 3.0, RRID:SCR_005056) [45] was then used to generate scaffolds with the Illumina reads from the long-insert libraries (2, 5, 10 kb and 20 kb). Redundans (version 0.14a) [46] with parameters (--identity 0.3 --overlap 0.3 --minLength 1000) was used to remove redundant scaffolds caused by the high heterozygosity of *C. nasus* genome.

Genome annotation

For repeat annotation, Repeat Modeler (version 1.04, RRID:SCR_015027) [47] and LTR_FINDER (version 1.06, RRID:SCR_015247) [48] were employed to construct a *de novo* repeat library with default parameters. RepeatMasker (version 3.2.9; RRID:SCR_012954) [49] was then used to search the repeat sequences against Repbase TE (version 14.04) [50] and the *de novo* repeat libraries in order to identify known and novel transposable elements (TEs) in *C. nasus* genome. The tandem repeats were identified by using Tandem Repeat Finder (version 4.04) [51], where the core parameters were set as "Match = 2, Mismatch = 7, Delta = 7, PM = 80, PI = 10, Minscore = 50, and MaxPerid = 2000." Furthermore, the relevant TE proteins were screened in the *C. nasus* assembly using RepeatProteinMask (version 3.2.2) [49].

A combined annotation pipeline of three separate approaches, including homology, *de novo*, and transcriptome-based annotations were employed to predict gene structures and functions. For the homology annotation, protein sequences from zebrafish, Japanese fugu, spotted green pufferfish, Japanese medaka, and stickleback (Ensembl release 75) were downloaded to map onto the *C. nasus* genome using Blat (e-value ≤1E-5; version 319, RRID:SCR_011919) [52]. Genewise (version 2.2.0,

RRID:SCR_015054) [53] was then employed to predict the potential gene structures based on all the alignments generated from the previous step. Short genes (less than 150 bp) and prematurely terminated or frame-shifted genes were discarded. For the *de novo* annotation, 1,000 complete genes were randomly chosen from the homology annotation set to train parameters for AUGUSTUS (version 3.0.2, RRID:SCR_008417) [54]. Repeat regions were masked by "N" in our genome assembly. AUGUSTUS was then utilized to make *de novo* predictions based on the repeat-masked genome assembly. The *de novo* annotation results were filtered using the same method for the homology prediction. For the transcriptome-based annotation, total RNA was extracted from the muscle and liver tissues from the same female fish for whole genome sequencing. The sequencing reads were aligned onto the genome assembly using HISAT2 (version 0.1.6, RRID:SCR_015530) [55]. The sorted and integrated steps were performed in these alignments. Cufflink (version 2.2.1, RRID:SCR_014597) [56] was then employed to identify potential gene structures. The results obtained by all three annotation methods were merged to produce a comprehensive and non-redundant gene set using Maker (version 2.31.8, RRID:SCR_005318) [57].

All the protein sequences obtained from the Maker results were mapped onto the SwissProt and TrEMBL databases [58] by BLASTP (version 2.2.25, RRID:SCR_001010) [59] with an E-value ≤ 1e-5 to find the best hit for each protein. We also employed the InterProScan (version 4.7, RRID:SCR_005829) [60] to align the protein sequences against other public databases, including Pfam [61], PRINTS

[62], ProDom [63] and SMART [64], in order to determine the known motifs and domains in our protein sequences. Finally, 20,300 genes proved to contain at least one functional assignment from public databases, including Swiss-Prot and TrEMBL [58], Gene Ontology (GO; [65]) and Kyoto Encyclopedia of Genes and Genomes (KEGG) [66] (Supplementary Table 5).

RAD sequencing and genotyping

RAD sequencing [9] was performed to generate a set of SNP markers from a full-sib family F1 group. In brief, the procedure was described as follows.

DNA extraction and sequencing. Genomic DNA from the 104 offspring individuals and their parents was separately extracted from the fin clips using a Mag Attract HMW DNA Kit (Qiagen, Gaithersburg, MD, USA). *Pst*I restriction enzyme was used for digestion of DNA, and for constructing the RAD sequencing libraries, which were subsequently sequenced on an Illumina HiSeq 2500 platform. The adapters of raw reads and the reads with low quality were filtered with a local perl script.

SNP calling. The BWA-MEM (version: 0.7.12, RRID:SCR_010910) [43] was used to align cleaned reads upon the second version of genome assembly. Subsequently, GATK (version: 3.1, RRID:SCR_001876) [67] was used to perform SNP calling. Related parameters for GATK was set as “QD < 2.0 || FS > 60.0 || MQ <40.0 || MQRankSum < -12.5 || ReadPosRankSum < -8.0”.

Construction of the genetic linkage map, chromosomal map and identification of

synteny blocks

JoinMap (version 4.1, RRID:SCR_009248) [68] with logarithm of odds values ranging from 2–12 was employed to evaluate the map distance under regression mapping algorithm. Subsequently, we constructed a high density genetic linkage map with 24 linkage groups, which is consistent with the results of a previous report [69].

Based on the SNP markers and genetic linkage map, a preliminary chromosomal-level assembly was generated. Locations of the scaffolds in each chromosome were fixed according to the following rules. For the scaffolds with sufficient SNP markers (more than two), we chose the two markers with the highest quality to determine their location and direction. However, directions of those scaffolds with insufficient SNP markers (only one) were not fixed, but instead they were placed directly onto the chromosomes. The protein alignments were conducted by performing BLASTP with an E-value < 1e-5. Then, MCscan (version 0.8) [70] was used to identify the gene-level syteny blocks from the BLASTP alignments with the parameter setting as "-a -e 1e-5 -s 5 -u 1".

SNP calling and phylogenetic analysis

The population sequencing reads were aligned onto our genome assembly (first version) using BWA-MEM (version 0.7.1, RRID:SCR_010910) [43]. SNP calling was performed using a standard GATK (version 3.1, RRID:SCR_001876) [67]. Quality filtering was realized for the raw variant calls using GATK with the following cut-offs: QD < 2.0, MQ < 40.0, FS > 60.0, MQRankSum ≤ 12.5, ReadPosRankSum ≤ 8.0, and DP < 100. The variants with more than 10% missing data were excluded and used a

minor allele frequency filter of 10%. Then SnpEff (version 3.4, RRID:SCR_005191) [71] was used to annotate the genetic variants and categorized the variants into coding (synonymous and non-synonymous), upstream/downstream, and intronic/intergenic classes. PLINK (version 1.07, RRID:SCR_001757) [72] with parameters "--distance 1-ibs flat-missing" was used to calculate the genetic distances among individuals, which were subsequently used to generate neighbor-joining trees with fneighbor (PHYLIPNEW v3.69.650 within the package of EMBOSS v 6.6.0.0, RRID:SCR_006244) [73].

Identification of selective sweep regions

Reduction of diversity was defined as ROD = $1 - \pi_{freshwater}/\pi_{migration}$, in which the $\pi_{freshwater}$ and $\pi_{migration}$ are the average numbers of nucleotide differences per site [74] from the freshwater and the migratory groups, respectively. The Fst and ROD values in a sliding window of 5 kb along the genome assembly were calculated using the entire SNP set. Genomic regions located in the top right corner of Figure 3a, corresponding to a 5% significant level of the FST and ROD values (above 0.79 and 0.59, respectively), were considered the selective sweep regions. Finally, 150 genes were identified in this region and these genes were enriched in GO terms using the Enrich Pipeline as described previously [75]. EnrichmentPipeline (http://www.ipm.ioz.ac.cn/kang/webpages/locusttranscriptome.html) for a given gene list was carried out based on the algorithm implemented in GOstat, with the whole annotated gene set as the background. GOstat tests for GO terms that are represented by significantly more genes in a given gene set using chi-square test. Fisher's exact

test was used when expected counts are below 5, which makes the chi-square test inaccurate.

Transcriptome analysis of freshwater and migratory individuals and validation by quantitative RT-PCRs

For transcriptome sequencing, total RNA was extracted from the brain tissues of three randomly selected individuals in the migratory or freshwater groups using TRIzol reagent (Invitrogen, Carlsbad, CA, USA). 125-bp paired-end Illumina reads were generated by a HiSeq 4000 platform for transcriptome sequencing. Raw data produced from the sequencing platform were filtered by removing reads contaminated with adaptors, more than 10% of N bases and more than 50% of low-quality bases (base quality score $\leqslant$ 10). These cleaned RNA reads were aligned onto the reference genome (first version) using HISAT2 (version 0.1.6, RRID:SCR_015530) with parameters "--phred33 --sensitive --no-discordant --no-mixed -I 1 -X 1000" [55]. Expression values were calculated by Cufflink (version 2.2.1, RRID:SCR_014597) with defaulted parameters [56]. The Cuffdiff in Cufflink package with parameters "-FDR 0.05 --geometric-norm TRUE –c 10" was used to identified the significantly differentially expressed genes (DEGs). The edgeR software (RRID:SCR_012802) [76] was to draw the heatmap view with the threshold: P-value <0.05 and folds >2. Finally, the enriched GO terms were identified for these DEGs using the Enrich Pipeline as described previously [75].

For the quantitative RT-PCR, brain tissues were obtained from five individuals in each group, and total RNA was extracted separately with TRIzol reagent (Invitrogen,

Carlsbad, CA, USA). First-strand cDNA was subsequently synthesized using a PrimeScript™ RT reagent kit with gDNA Eraser (Takara, Kusatsu, Shiga, Japan), and 18S RNA was used as the internal control. Sequences of the primer pairs are provided in Supplementary Table 15. Transcription of the target genes was calculated as the relative increase according to the $2^{-\Delta\Delta CT}$ method [77]. Normal distribution and homogeneity of variance of data was tested with the Shapiro-Wilk and Levene tests ($\alpha = 0.05$), respectively. Then differences in the mRNA levels were compared by the students' t-test using IBM SPSS Statistics 22.0 (IBM Inc., Chicago, IL, USA). P values of < 0.05 were considered statistically significant.

Measurement of Sr and Ca contents in otoliths

The Sr and Ca contents in otoliths were measured as described in our previous report [3]. In brief, the otoliths were embedded in epoxy resin (EpoFix, Struers, Copenhagen, Denmark) for grinding and polishing to expose their cores with an automated grinding machine (Roto Pol-35, Struers, Kentucky, USA). After cleaning in an ultrasonic bath, rinsed by deionized water, and carbon-coated with a high-vacuum evaporator (JEE-420, JEOL Ltd., Tokyo, Japan), the samples were measured using a wave-dispersive X-ray electron probe micro-analyzer (JXA-8100, JEOL Ltd, Welwyn Garden City, UK). Tausonite ($SrTiO_3$) and calcite ($CaCO_3$) were used as the internal standards.

Supplementary materials

Supplementary data associated with this article can be found in the online version.

Availability of supporting data

Genome assemblies reported here have been deposited at the GenBank under the project ID PRJNA421870. Genome *de novo*, population genome sequencing, RAD and transcriptome sequencing data have been deposited at the NCBI Short Read Archive (SRA) under the project ID PRJNA422339.

Abbreviations

Adcy1, adenylate cyclase 1; *Acoxl*, acyl-coenzyme A oxidase-like protein; *Atp2a3*, ATPase sarcoplasmic/endoplasmic reticulum Ca2+ transporting 3; Ca, calcium; *Cacna1a*, calcium voltage-gated channel subunit alpha1 A; *Cacnalg*, voltage-dependent T-type calcium channel subunit alpha-1G; DEGs, differentially expressed genes; *Egfr*, epidermal growth factor receptor; Fst, fixation index for diversity differentiation; *Flnb*, filamin B; *Fzd1*, frizzled-1; Gb, gigabase; Gbp, GSK-3-binding protein; GO, gene ontology; KEGG, Kyoto Encyclopedia of Genes and Genomes; *Pdgfrb*, platelet derived growth factor receptor beta; Ppp2r1b, serine/threonine-protein phosphatase 2A 65 kDa regulatory subunit A beta isoform; RAD, restriction-site associated DNA; ROD, reduction of diversity; *Ryr2*, ryanodine receptor 2; *Slc8a1*, solute carrier family 8 member A1; *Smad4*, SMAD family member 4; SNP, single nucleotide polymorphism; Sr, strontium; TE, transposable element; *Tgfbr2*, transforming growth factor beta receptor 2.

Competing interests

The authors declare no competing financial interests.

Funding

This study was supported by grants from the National Natural Science Foundation of China (Nos. 31672643, 31372533, 31502152), the General Program of Natural Science Foundation of Jiangsu Province of China (No. BK20191145), Three New Projects of Agricultural Aquaculture Program of Jiangsu Province (No. Y2018-17), and the Special Fund of Jiangsu Province for the Transformation of Scientific and Technological Achievements (No. BA2015167).

Author contributions

P.X. conceived the study and designed the project. G.X. managed the project. K.L., D.X., Y.W., Q.L., N.S., J. C., C. S., Y. Z. and Z.J.N. prepared all samples used in this study. C.B. performed genome assembly, annotation, resequencing data analyses and transcriptome expression calculation. J.L. constructed the genetic map and chromosomal map. Y.H. and Y.L. implemented phylogenetic analysis. H.L., J.Y. and T.J. measured the Sr and Ca contents of otoliths. P.X., Q.S., G.X., C.B., J.L., X.Y, R.G., W.G. and J.X. discussed the data. C.B., G.X. and J.L. wrote the manuscript. Q.S., G.X and P.X. revised the manuscript. All authors contributed to data interpretation.

References

1. Chapman BB, Hulthen K, Brodersen J, Nilsson PA, Skov C, Hansson LA, Bronmark C: **Partial migration in fishes: causes and consequences**. *Journal of fish biology* 2012, **81**(2):456-478.
2. Ueda H: **Physiological mechanism of homing migration in Pacific salmon from behavioral to molecular biological approaches**. *General and comparative endocrinology* 2011, **170**(2):222-232.
3. Jiang T, Yang J, Lu MJ, Liu HB, Chen TT, Gao YW: **Discovery of a spawning area for anadromous Coilia nasus Temminck et Schlegel, 1846 in Poyang Lake, China**. *Journal of Applied Ichthyology* 2017.
4. Jiang T, Yang J, Liu H, Shen X-q: **Life history of Coilia nasus from the Yellow Sea inferred from otolith Sr:Ca ratios**. *Environmental Biology of Fishes* 2012, **95**(4):503-508.
5. Li WX, Song R, Wu SG, Zou H, Nie P, Wang GT: **Seasonal Occurrence of Helminths in the Anadromous Fish Coilia nasus**. *Journal of Parasitology* 1937, **97**(2):192.
6. Liu D, Li Y, Tang W, Yang J, Guo H, Zhu G, Li H: **Population structure of Coilia nasus in the Yangtze River revealed by insertion of short interspersed elements**. *Biochemical Systematics & Ecology* 2014, **54**(Complete):103-112.
7. Secor DH, Kerr L: **Lexicon of life cycle diversity in diadromous and other fishes**, vol. 69; 2009.
8. Chapman BB, Skov C, Hulthén K, Brodersen J, Nilsson PA, Hansson LA, Brönmark C: **Partial migration in fishes: definitions, methodologies and taxonomic distribution**. *Journal of Fish Biology* 2012, **81**(2):479.
9. Yu H, You X, Li J, Zhang X, Zhang S, Jiang S, Lin X, Lin HR, Meng Z, Shi Q: **A genome-wide association study on growth traits in orange-spotted grouper (Epinephelus coioides) with RAD-seq genotyping**. *Science China Life sciences* 2018, **61**(8):934-946.
10. Song L, Bian C, Luo Y, Wang L, You X, Li J, Qiu Y, Ma X, Zhu Z, Ma L: **Draft genome of the Chinese mitten crab, Eriocheir sinensis**. *GigaScience,5,1(2016-01-28)* 2016, **5**(1):5.
11. Simao FA, Waterhouse RM, Ioannidis P, Kriventseva EV, Zdobnov EM: **BUSCO: assessing genome assembly and annotation completeness with single-copy orthologs**. *Bioinformatics* 2015, **31**(19):3210-3212.
12. Brown RJ, Severin KP: **Otolith chemistry analyses indicate that water Sr:Ca is the primary factor influencing otolith Sr:Ca for freshwater and diadromous fish but not for marine fish**. *Canadian Journal of Fisheries & Aquatic Sciences* 2009, **66**(10):1790-1808.
13. Limburg KE, Olson C, Walther Y, Dale D, Slomp CP, Høie H: **Tracking Baltic hypoxia and cod migration over millennia with natural tags**. *Proceedings of the National Academy of Sciences of the United States of America* 2011, **108**(22):E177.
14. Yang J, Jiang T, Liu H: **Are there habitat salinity markers of the Sr:Ca ratio in the otolith of wild diadromous fishes? A literature survey**. *Ichthyological Research* 2011, **58**(3):291-294.
15. Chen TT, Jiang T, Liu HB, Li MM, Yang J: **Do all long supermaxilla - type estuarine tapertail anchovies (Coilia nasus Temminck et Schlegel, 1846) migrate anadromously?** *Journal of Applied Ichthyology* 2017, **33**(2).
16. Knighton DR, Zheng JH, Eyck LT, Ashford VA, Xuong NH, Taylor SS, Sowadski JM: **Crystal structure of the catalytic subunit of cyclic adenosine monophosphate-dependent protein kinase**. *Science* 1991, **253**(5018):407.

17. Kohli G, Hu S, Clelland E, Di Muccio T, Rothenstein J, Peng C: **Cloning of Transforming Growth Factor-β1 (TGF-β1) and Its Type II Receptor from Zebrafish Ovary and Role of TGF-β1 in Oocyte Maturation**. *Endocrinology* 2003, **144**(5):1931-1941.

18. Xu G, Du F, Li Y, Nie Z, Xu P: **Integrated application of transcriptomics and metabolomics yields insights into population-asynchronous ovary development inCoilia nasus**. *Scientific Reports* 2016, **6**:31835.

19. Lapointe E, Boyer A, Rico C, Paquet M, Franco HL, Gossen J, DeMayo FJ, Richards JS, Boerboom D: **FZD1 regulates cumulus expansion genes and is required for normal female fertility in mice**. *Biology of reproduction* 2012, **87**(5):104.

20. Dickinson ME, Flenniken AM, Ji X, Teboul L, Wong MD, White JK, Meehan TF, Weninger WJ, Westerberg H, Adissu H *et al*: **High-throughput discovery of novel developmental phenotypes**. *Nature* 2016, **537**(7621):508-514.

21. Zwingman TA, Neumann PE, Noebels JL, Herrup K: **Rocker is a new variant of the voltage-dependent calcium channel gene Cacna1a**. *The Journal of neuroscience : the official journal of the Society for Neuroscience* 2001, **21**(4):1169-1178.

22. Miki T, Zwingman TA, Wakamori M, Lutz CM, Cook SA, Hosford DA, Herrup K, Fletcher CF, Mori Y, Frankel WN *et al*: **Two novel alleles of tottering with distinct Ca(v)2.1 calcium channel neuropathologies**. *Neuroscience* 2008, **155**(1):31-44.

23. Yamaguchi T, Kato M, Fukui M, Akazawa K: **Rolling mouse Nagoya as a mutant animal model of basal ganglia dysfunction: determination of absolute rates of local cerebral glucose utilization**. *Brain research* 1992, **598**(1-2):38-44.

24. Alberici P, Jagmohan-Changur S, De Pater E, Van Der Valk M, Smits R, Hohenstein P, Fodde R: **Smad4 haploinsufficiency in mouse models for intestinal cancer**. *Oncogene* 2006, **25**(13):1841-1851.

25. Elaib Z, Adam F, Berrou E, Bordet JC, Prevost N, Bobe R, Bryckaert M, Rosa JP: **Full activation of mouse platelets requires ADP secretion regulated by SERCA3 ATPase-dependent calcium stores**. *Blood* 2016, **128**(8):1129-1138.

26. Zhou X, Tian F, Sandzen J, Cao R, Flaberg E, Szekely L, Cao Y, Ohlsson C, Bergo MO, Boren J *et al*: **Filamin B deficiency in mice results in skeletal malformations and impaired microvascular development**. *Proceedings of the National Academy of Sciences of the United States of America* 2007, **104**(10):3919-3924.

27. Yamaguchi N, Takahashi N, Xu L, Smithies O, Meissner G: **Early cardiac hypertrophy in mice with impaired calmodulin regulation of cardiac muscle Ca release channel**. *The Journal of clinical investigation* 2007, **117**(5):1344-1353.

28. Threadgill DW, Dlugosz AA, Hansen LA, Tennenbaum T, Lichti U, Yee D, LaMantia C, Mourton T, Herrup K, Harris RC *et al*: **Targeted disruption of mouse EGF receptor: effect of genetic background on mutant phenotype**. *Science* 1995, **269**(5221):230-234.

29. Koitabashi N, Bedja D, Zaiman AL, Pinto YM, Zhang M, Gabrielson KL, Takimoto E, Kass DA: **Avoidance of transient cardiomyopathy in cardiomyocyte-targeted tamoxifen-induced MerCreMer gene deletion models**. *Circulation research* 2009, **105**(1):12-15.

30. Neverman D, Wurtsbaugh WA: **The thermoregulatory function of diel vertical migration for a juvenile fish, Cottus extensus**. *Oecologia* 1994, **98**(3-4):247-256.

31. Liu SH, Zhao-Li XU, Tian FG: **Study on feeding habit of Coilia mystus in Yangtze River Estuary**. *Journal of Shanghai Ocean University* 2012, **21**(4):589-597.
32. Cooke CJ, Smith CJ, Newton RP, Walton TJ: **Binding saturation analysis of inositol-1,4,5-trisphosphate in suspension cultures of lucerne cells**. *Biochemical Society transactions* 1991, **19**(4):359S.
33. **Calcium Signaling Pathways**. *Biophysical Journal* 2008, **94**(2Suppl):150–157.
34. Seger R, Krebs EG: **The MAPK signaling cascade**. *FASEB journal : official publication of the Federation of American Societies for Experimental Biology* 1995, **9**(9):726-735.
35. Komiya Y, Habas R: **Wnt signal transduction pathways**. *Organogenesis* 2008, **4**(2):68-75.
36. Jianying NI: **Rencular structural indices and urinary concentrating capacity of Neophocaena phocaenoides**. *Acta Zoologica Sinica* 1988.
37. Wang FY, Fu WC, Wang IL, Yan HY, Wang TY: **The giant mottled eel, Anguilla marmorata, uses blue-shifted rod photoreceptors during upstream migration**. *PLoS ONE* 2014, **9**(8):e103953.
38. Gardiner JM, Whitney NM, Hueter RE: **Smells Like Home: The Role of Olfactory Cues in the Homing Behavior of Blacktip Sharks, Carcharhinus limbatus**. *Integrative and comparative biology* 2015, **55**(3):495-506.
39. Lin Q, Qiu Y, Gu R, Xu M, Li J, Bian C, Zhang H, Qin G, Zhang Y, Luo W *et al*: **Draft genome of the lined seahorse, Hippocampus erectus**. *GigaScience* 2017, **6**(6):1-6.
40. Luo R, Liu B, Xie Y, Li Z, Huang W, Yuan J, He G, Chen Y, Pan Q, Liu Y *et al*: **Erratum: SOAPdenovo2: an empirically improved memory-efficient short-read de novo assembler**. *GigaScience* 2015, **4**:30.
41. Kajitani R, Toshimoto K, Noguchi H, Toyoda A, Ogura Y, Okuno M, Yabana M, Harada M, Nagayasu E, Maruyama H *et al*: **Efficient de novo assembly of highly heterozygous genomes from whole-genome shotgun short reads**. *Genome research* 2014, **24**(8):1384-1395.
42. Ye C, Hill CM, Wu S, Ruan J, Ma ZS: **DBG2OLC: Efficient Assembly of Large Genomes Using Long Erroneous Reads of the Third Generation Sequencing Technologies**. *Scientific reports* 2016, **6**:31900.
43. Li H, Durbin R: **Fast and accurate short read alignment with Burrows-Wheeler transform**. *Bioinformatics* 2009, **25**(14):1754-1760.
44. Walker BJ, Abeel T, Shea T, Priest M, Abouelliel A, Sakthikumar S, Cuomo CA, Zeng Q, Wortman J, Young SK *et al*: **Pilon: An Integrated Tool for Comprehensive Microbial Variant Detection and Genome Assembly Improvement**. *PloS one* 2014, **9**(11):e112963.
45. Boetzer M, Henkel CV, Jansen HJ, Butler D, Pirovano W: **Scaffolding pre-assembled contigs using SSPACE**. *Bioinformatics* 2011, **27**(4):578-579.
46. Pryszcz LP, Gabaldon T: **Redundans: an assembly pipeline for highly heterozygous genomes**. *Nucleic acids research* 2016, **44**(12):e113.
47. Chen N: **Using RepeatMasker to identify repetitive elements in genomic sequences**. *Current protocols in bioinformatics / editoral board, Andreas D Baxevanis [et al]* 2004, **Chapter 4**:Unit 4 10.
48. Xu Z, Wang H: **LTR_FINDER: an efficient tool for the prediction of full-length LTR retrotransposons**. *Nucleic acids research* 2007, **35**(Web Server issue):W265-268.

49. Tarailo-Graovac M, Chen N: **Using RepeatMasker to identify repetitive elements in genomic sequences**. *Current protocols in bioinformatics / editoral board, Andreas D Baxevanis [et al]* 2009, **Chapter 4**:Unit 4 10.

50. Jurka J, Kapitonov VV, Pavlicek A, Klonowski P, Kohany O, Walichiewicz J: **Repbase Update, a database of eukaryotic repetitive elements**. *Cytogenetic and genome research* 2005, **110**(1-4):462-467.

51. Benson G: **Tandem repeats finder: a program to analyze DNA sequences**. *Nucleic acids research* 1999, **27**(2):573-580.

52. Bhagwat M, Young L, Robison RR: **Using BLAT to find sequence similarity in closely related genomes**. *Current protocols in bioinformatics / editoral board, Andreas D Baxevanis [et al]* 2012, **Chapter 10**:Unit10 18.

53. Birney E, Clamp M, Durbin R: **GeneWise and Genomewise**. *Genome research* 2004, **14**(5):988-995.

54. Stanke M, Keller O, Gunduz I, Hayes A, Waack S, Morgenstern B: **AUGUSTUS: ab initio prediction of alternative transcripts**. *Nucleic acids research* 2006, **34**(Web Server issue):W435-439.

55. Kim D, Langmead B, Salzberg SL: **HISAT: a fast spliced aligner with low memory requirements**. *Nature methods* 2015, **12**(4):357-360.

56. Trapnell C, Hendrickson DG, Sauvageau M, Goff L, Rinn JL, Pachter L: **Differential analysis of gene regulation at transcript resolution with RNA-seq**. *Nature biotechnology* 2013, **31**(1):46-53.

57. Cantarel BL, Korf I, Robb SM, Parra G, Ross E, Moore B, Holt C, Sanchez Alvarado A, Yandell M: **MAKER: an easy-to-use annotation pipeline designed for emerging model organism genomes**. *Genome research* 2008, **18**(1):188-196.

58. Bairoch A, Apweiler R: **The SWISS-PROT protein sequence database and its supplement TrEMBL in 2000**. *Nucleic acids research* 2000, **28**(1):45-48.

59. Mount DW: **Using the Basic Local Alignment Search Tool (BLAST)**. *CSH protocols* 2007, **2007**:pdb top17.

60. Zdobnov EM, Apweiler R: **InterProScan--an integration platform for the signature-recognition methods in InterPro**. *Bioinformatics* 2001, **17**(9):847-848.

61. Finn RD, Tate J, Mistry J, Coggill PC, Sammut SJ, Hotz HR, Ceric G, Forslund K, Eddy SR, Sonnhammer EL *et al*: **The Pfam protein families database**. *Nucleic acids research* 2008, **36**(Database issue):D281-288.

62. Attwood TK, Croning MD, Flower DR, Lewis AP, Mabey JE, Scordis P, Selley JN, Wright W: **PRINTS-S: the database formerly known as PRINTS**. *Nucleic acids research* 2000, **28**(1):225-227.

63. Bru C, Courcelle E, Carrere S, Beausse Y, Dalmar S, Kahn D: **The ProDom database of protein domain families: more emphasis on 3D**. *Nucleic acids research* 2005, **33**(Database issue):D212-215.

64. Letunic I, Copley RR, Pils B, Pinkert S, Schultz J, Bork P: **SMART 5: domains in the context of genomes and networks**. *Nucleic acids research* 2006, **34**(Database issue):D257-260.

65. Ashburner M, Ball CA, Blake JA, Botstein D, Butler H, Cherry JM, Davis AP, Dolinski K, Dwight SS, Eppig JT *et al*: **Gene ontology: tool for the unification of biology. The Gene Ontology Consortium**. *Nature genetics* 2000, **25**(1):25-29.

66. Kanehisa M, Goto S: **KEGG: kyoto encyclopedia of genes and genomes**. *Nucleic acids research* 2000, **28**(1):27-30.

67. Mckenna A, Hanna M, Banks E, Sivachenko A, Cibulskis K, Kernytsky A, Garimella K, Altshuler D, Gabriel S, Daly M: **The Genome Analysis Toolkit: a MapReduce framework for analyzing next-generation DNA sequencing data**. *Genome Research* 2010, **20**(9):1297-1303.

68. Stam P: **Construction of integrated genetic linkage maps by means of a new computer package: Join Map**. *The Plant Journal* 2005, **3**(5):739-744.

69. Shijie Xu YL, Guanbao Fu, Haowei Wu, Qian Wang, Qigen Liu, Xiancheng Qu **Chromosome karyotype analysis of Coilia nasus**. *Guangdong Agricultural Sciences* 2014, **7**:155-157.

70. Tang H, Wang X, Bowers JE, Ming R, Alam M, Paterson AH: **Unraveling ancient hexaploidy through multiply-aligned angiosperm gene maps**. *Genome research* 2008, **18**(12):1944-1954.

71. Cingolani P, Platts A, Wang le L, Coon M, Nguyen T, Wang L, Land SJ, Lu X, Ruden DM: **A program for annotating and predicting the effects of single nucleotide polymorphisms, SnpEff: SNPs in the genome of Drosophila melanogaster strain w1118; iso-2; iso-3**. *Fly (Austin)* 2012, **6**(2):80-92.

72. Purcell S, Neale B, Todd-Brown K, Thomas L, Ferreira MA, Bender D, Maller J, Sklar P, de Bakker PI, Daly MJ *et al*: **PLINK: a tool set for whole-genome association and population-based linkage analyses**. *American journal of human genetics* 2007, **81**(3):559-575.

73. Retief JD: **Phylogenetic analysis using PHYLIP**. *Methods in molecular biology* 2000, **132**:243-258.

74. Berg PR, Jentoft S, Star B, Ring KH, Knutsen H, Lien S, Jakobsen KS, Andre C: **Adaptation to Low Salinity Promotes Genomic Divergence in Atlantic Cod (Gadus morhua L.)**. *Genome biology and evolution* 2015, **7**(6):1644-1663.

75. Chen S, Yang P, Jiang F, Wei Y, Ma Z, Kang L: **De novo analysis of transcriptome dynamics in the migratory locust during the development of phase traits**. *PloS one* 2010, **5**(12):e15633.

76. Robinson MD, McCarthy DJ, Smyth GK: **edgeR: a Bioconductor package for differential expression analysis of digital gene expression data**. *Bioinformatics* 2010, **26**(1):139-140.

77. Livak KJ, Schmittgen TD: **Analysis of relative gene expression data using real-time quantitative PCR and the 2(-Delta Delta C(T)) Method**. *Methods* 2001, **25**(4):402-408.

Figures legends

Figure 1. Seasonal migration and migratory dimorphism of the Chinese tapertail anchovy. (**a**) A representative image of this economically important fish. (**b**) Geographic distribution of the collected samples along the putative migration route. The red five pointed stars represent the sample collection sites (see more details in Table 1) and the green arrows indicate the direction of reproductive migration. (**c**) Neighbor-joining phylogenetic tree constructed with genome-wide SNPs. The scale bar represents the similarity level. (**d**) Representative X-ray intensity maps of the Sr content in the otoliths of *C. nasus*. The constant blue color represents the freshwater residential pattern, while the alternative blue and green colors indicate the migratory pattern.

Figure 2. A Circos figure of the genome assembly. The rings from the outside to the inside are in the order of (**A**) pseudo-chromosomes, (**B**) a genetic map, (**C**) a heat map of gene density (in orange) in 100 kb of non-overlapping windows, (**D**) line chart of the genome GC content in 100 kb of non-overlapping windows, and (**E**) a heat map of repeat density (in violet) in 100 kb of non-overlapping windows. Syntenic blocks are connected with navy lines and each line indicates one paralog gene pair in the assembled genome.

Figure 3. Comparison of selection sweep regions in the freshwater residential and migratory groups. (**a**) Distributions of ROD and Fst values in 5-kb non-overlapping windows. Red dots denote windows with the top 5% ROD and Fst values. (**b**) Migratory adaptation-related genes distributed on 11 chromosomes. Examples of

genes (**c, d**) with selection sweep signals identified by $\pi_{migration}/\pi_{freshwater}$, Fst, and ROD values using a 5-kb sliding window. Blue and red lines represent the $\pi_{migration}$ and $\pi_{freshwater}$, respectively. Dashed lines denote the threshold of top 5%.

Figure 4. Three enriched Ca^{2+}-related pathways. The genes highlighted in red were positively selected for the migratory adaptation. The green lines and arrows indicate positive regulation and the red ones indicate negative regulation. Interestingly, 14 of the selected genes (highlighted in red) potentially participate in the three critical Ca^{2+}-related pathways, including calcium signaling pathway, MAKP signaling pathway, and Wnt signaling pathway.

Figure 5. Representative mRNA transcription and protein structural changes in the selected genes within the three Ca^{2+}-related pathways. (**a–f**) Quantitative RT-PCR validation of the mRNA transcription differences in six representative genes. (**g**) A heatmap of the DEGs in the three Ca^{2+}-related pathways based on the brain transcriptome. (**i**) Changes in the tertiary protein structure of Tgfr2.

Tables

Table 1. Summary of sample information for the genome resequencing.

Type	Locality	Sample	No.	Position
**Sea**	Yellow Sea	S	15	N 31°30’ E 122°24’
**River**	Chongming	E	15	N 31°46’ E 121°7’
	Nantong	2R	15	N 31°58’ E 120°49’
	Jingjiang	3R	11	N 31°56’ E 120°14’
	Anqing	4R	13	N 30°30’ E 117°4’
**Lake**	Hukou	5R	13	N 29°44’ E 116°12’
	Duchang	6R	14	N 29°14’ E 116°17’

Table 2. Statistics of the genome assembly of *C. nasus*.

**Genome assembly**	**Parameter**
Contig N50 (Mb)	1.6
Contig number (>100 bp)	1,327
Scaffold N50 (Mb)	2.1
Scaffold number (>100 bp)	727
Total length (Mb)	870.0
Genome coverage (×)	404.4
The longest scaffold (Mb)	12.0
**Genome annotation**	
Protein-coding gene number	20,837
Mean transcript length (bp)	16,775.5
Mean exons per gene	10.1
Mean exon length (bp)	1,759.7
Mean intron length (bp)	1,476.0

Figure 1 Click here to access/download;Figure;Figure 1.pdf

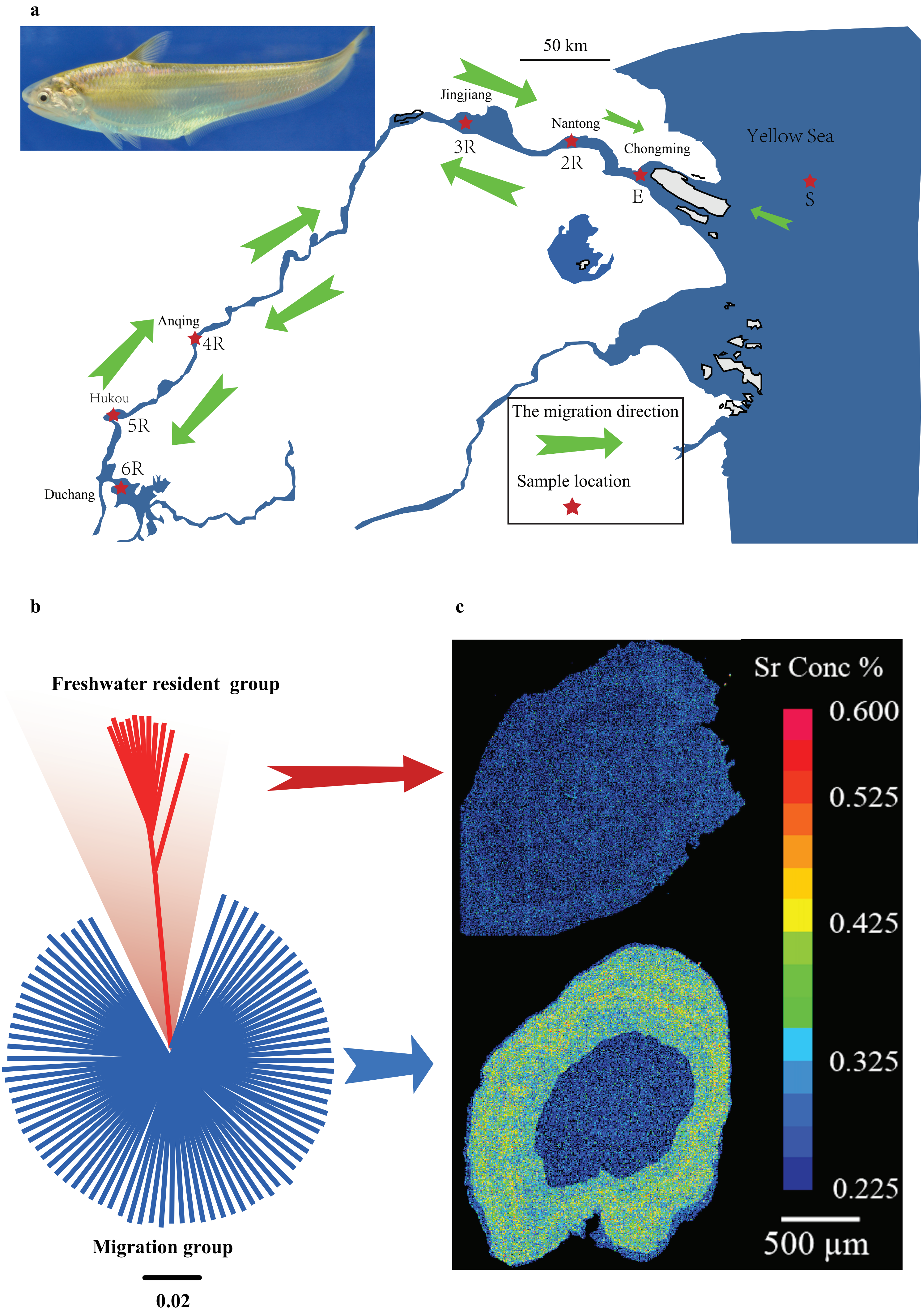

Figure 2

Click here to access/download;Figure;Figure 2.pdf

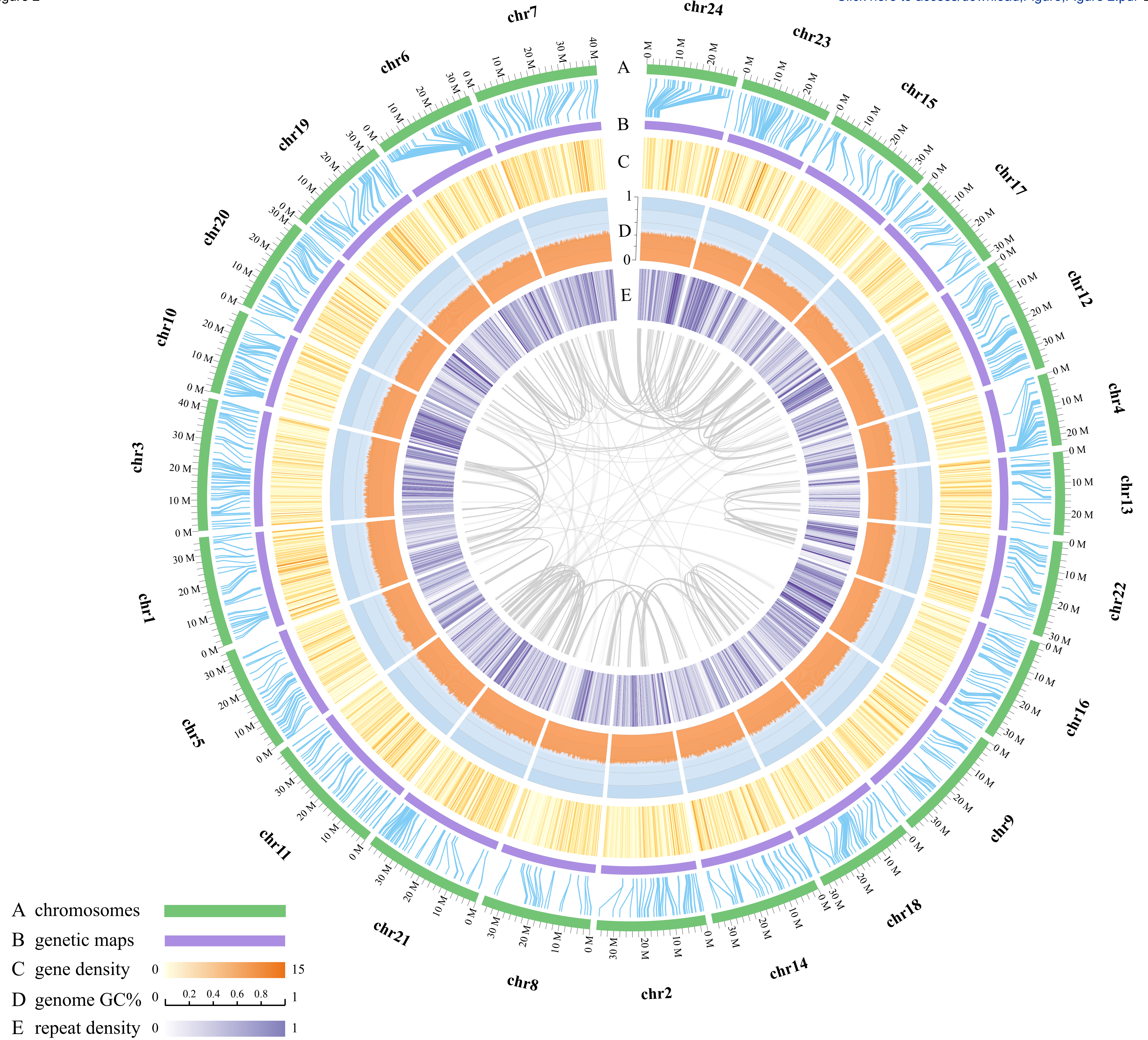

Figure 3

Click here to access/download;Figure;Figure 3.pdf

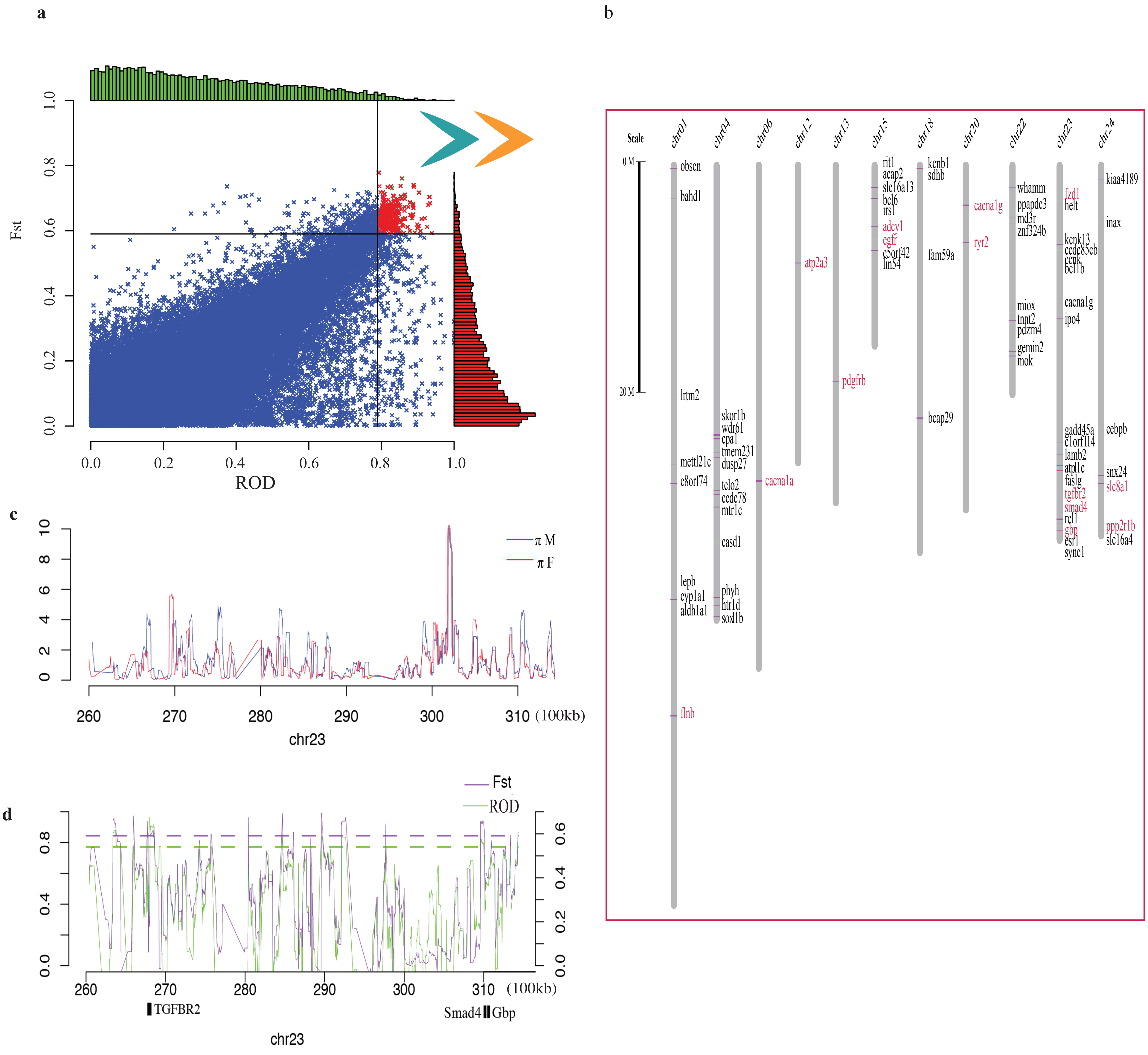

Figure 4

Click here to access/download;Figure;Figure 4.pdf

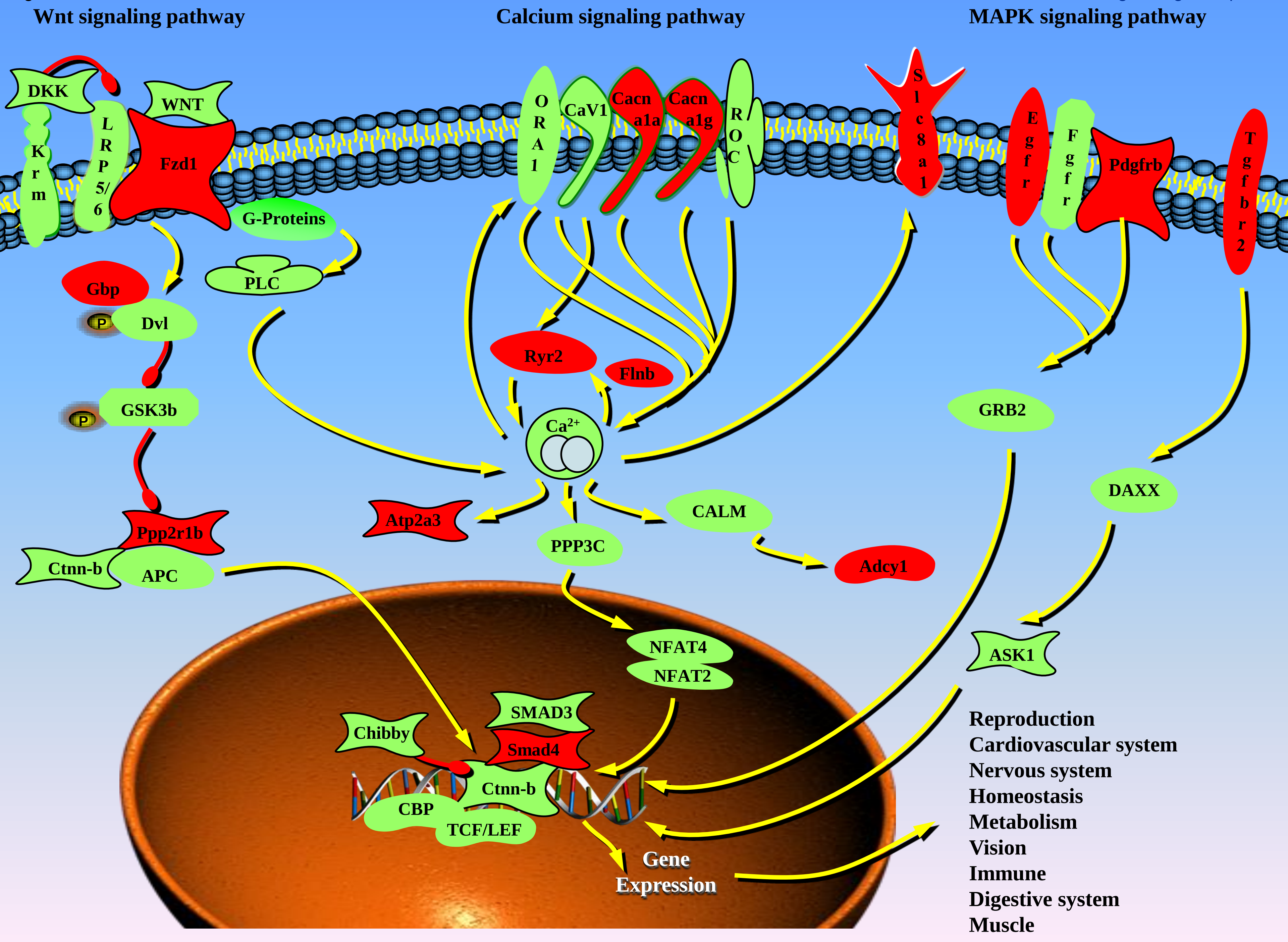

Figure 5

Click here to access/download;Figure;Figure 5.pdf

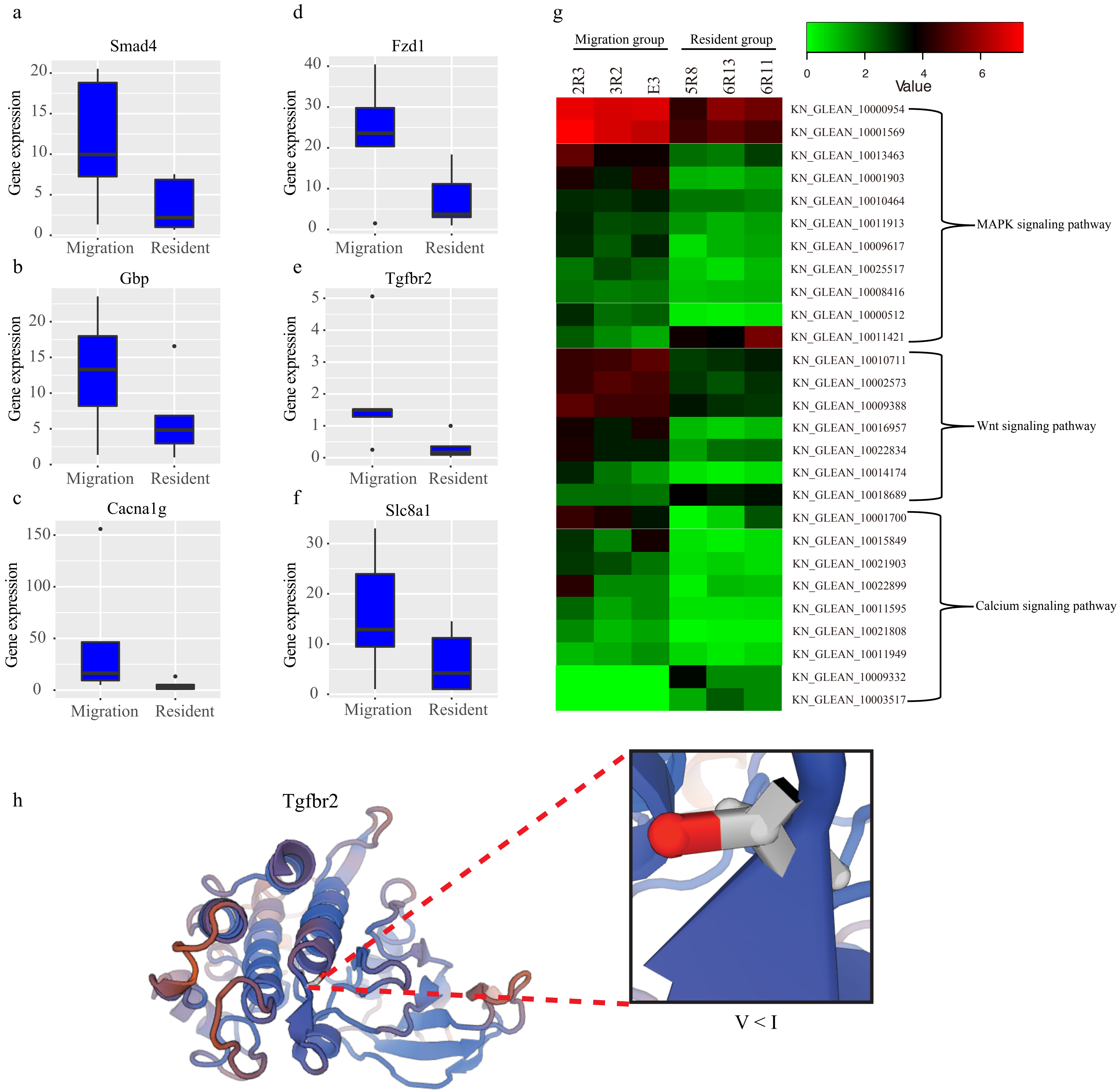

Supplementary Table 11

Click here to access/download
Supplementary Material
Supplementary Table 11.xlsx

Supplementary Table 13

Click here to access/download
Supplementary Material
Supplementary Table 13.xlsx

Click here to access/download
Supplementary Material
Supplementary Table 14.xlsx

Click here to access/download
Supplementary Material
Supplementary materials revised.docx
