# Supplementary material for: Genome and population sequencing of a chromosome-level genome assembly of the Chinese tapertail anchovy (Coilia nasus) provides novel insights into migratory adaptation
Source: Gigascience. 2020 Jan 2;9(1):giz157. doi: 10.1093/gigascience/giz157 (PMC6939831; doi:10.1093/gigascience/giz157)
Supplement: giz157_Supplemental_Figures_and_Tables [file giz157_supplemental_figures_and_tables.zip › Supplementary materials revised.docx]

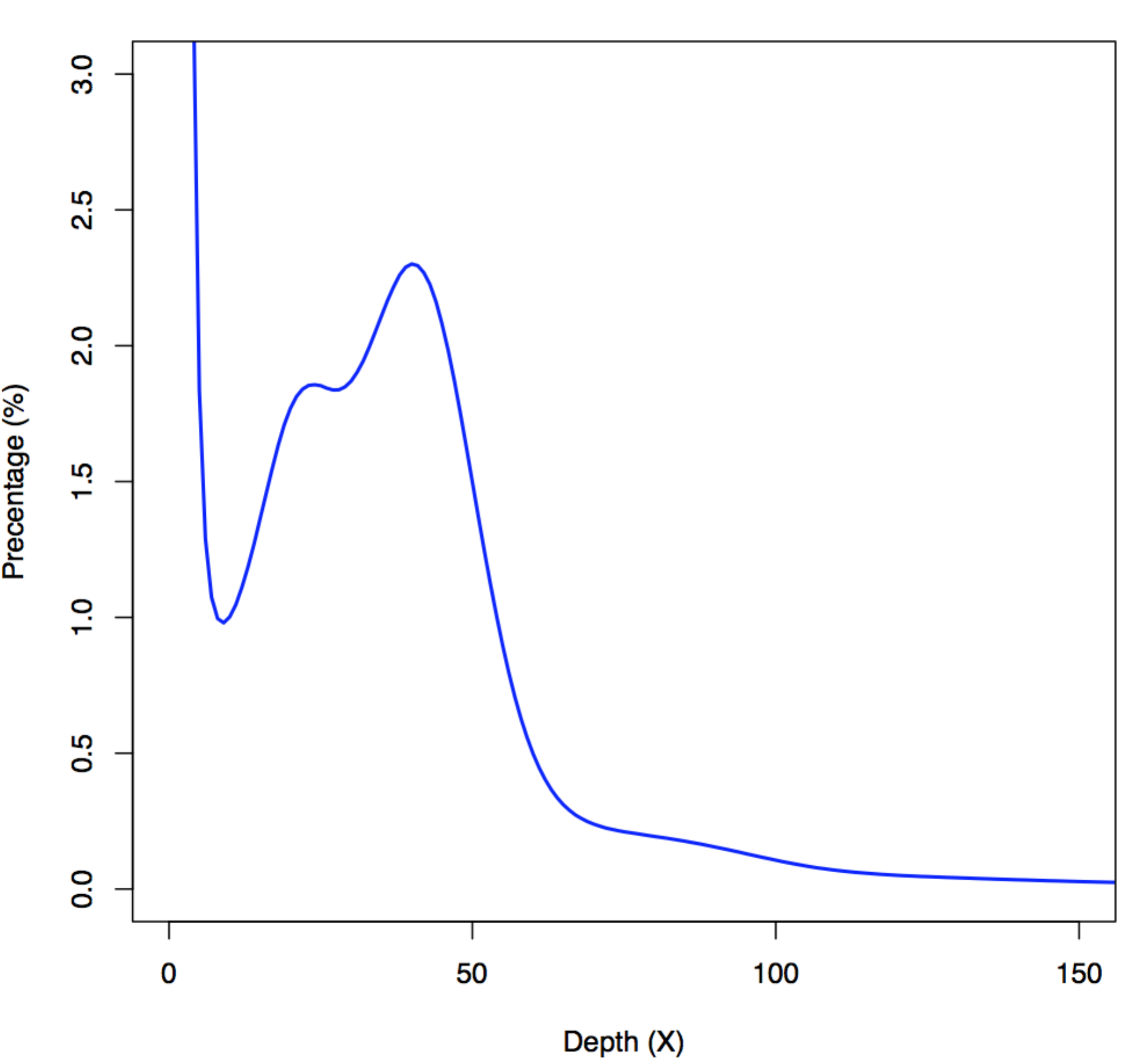


**Supplementary Figure 1.** A *K*-mer view of the sequenced *Coilia nasus* genome.

**
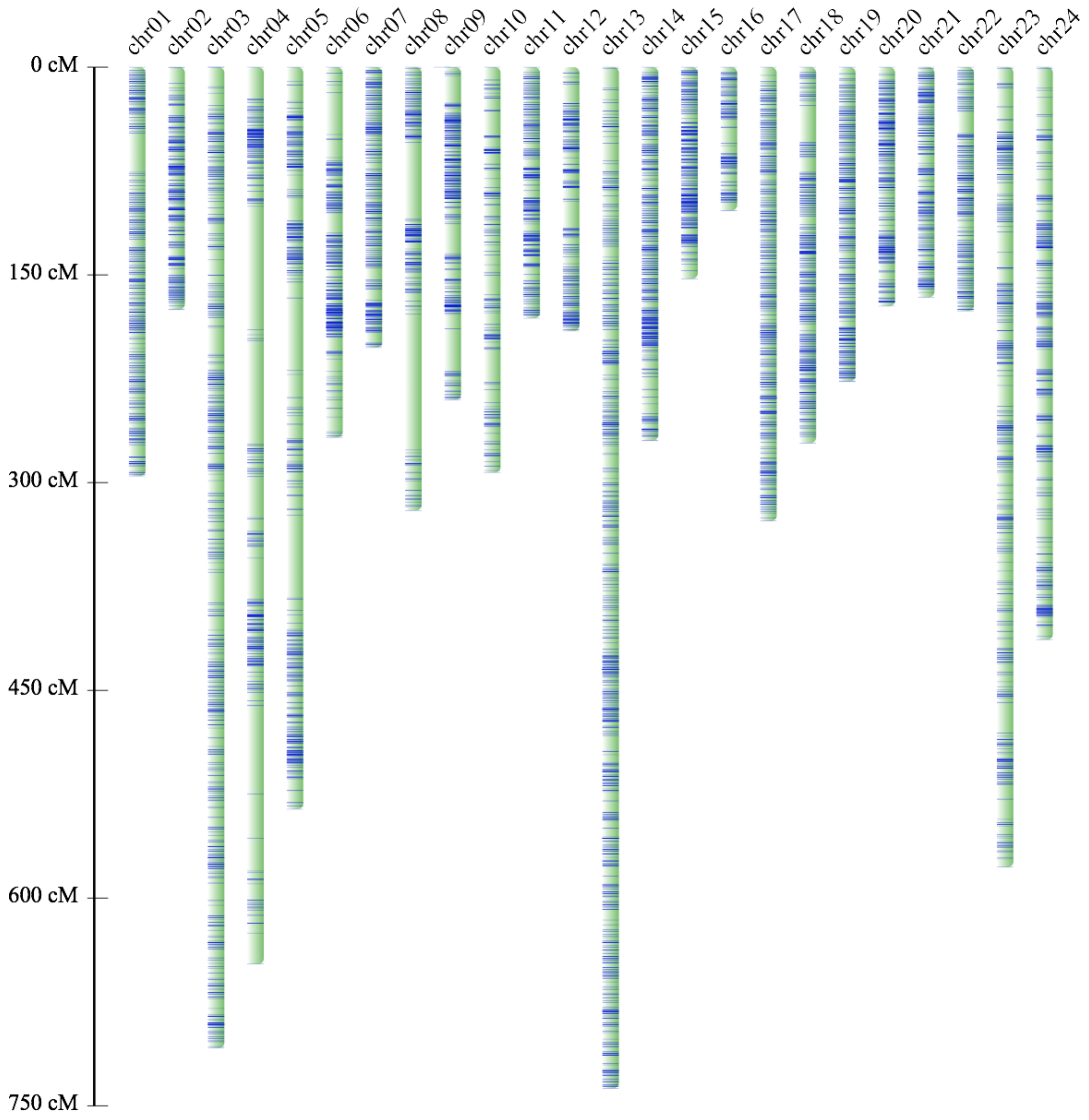
**

**Supplementary Figure 2.** Construction of linkage groups (or pseudo-chromosomes) of *C. nasus* by RAD sequencing. The light green bars depict the pseudo-chromosomes, and the blue lines represent the position of SNP markers in each chromosome.


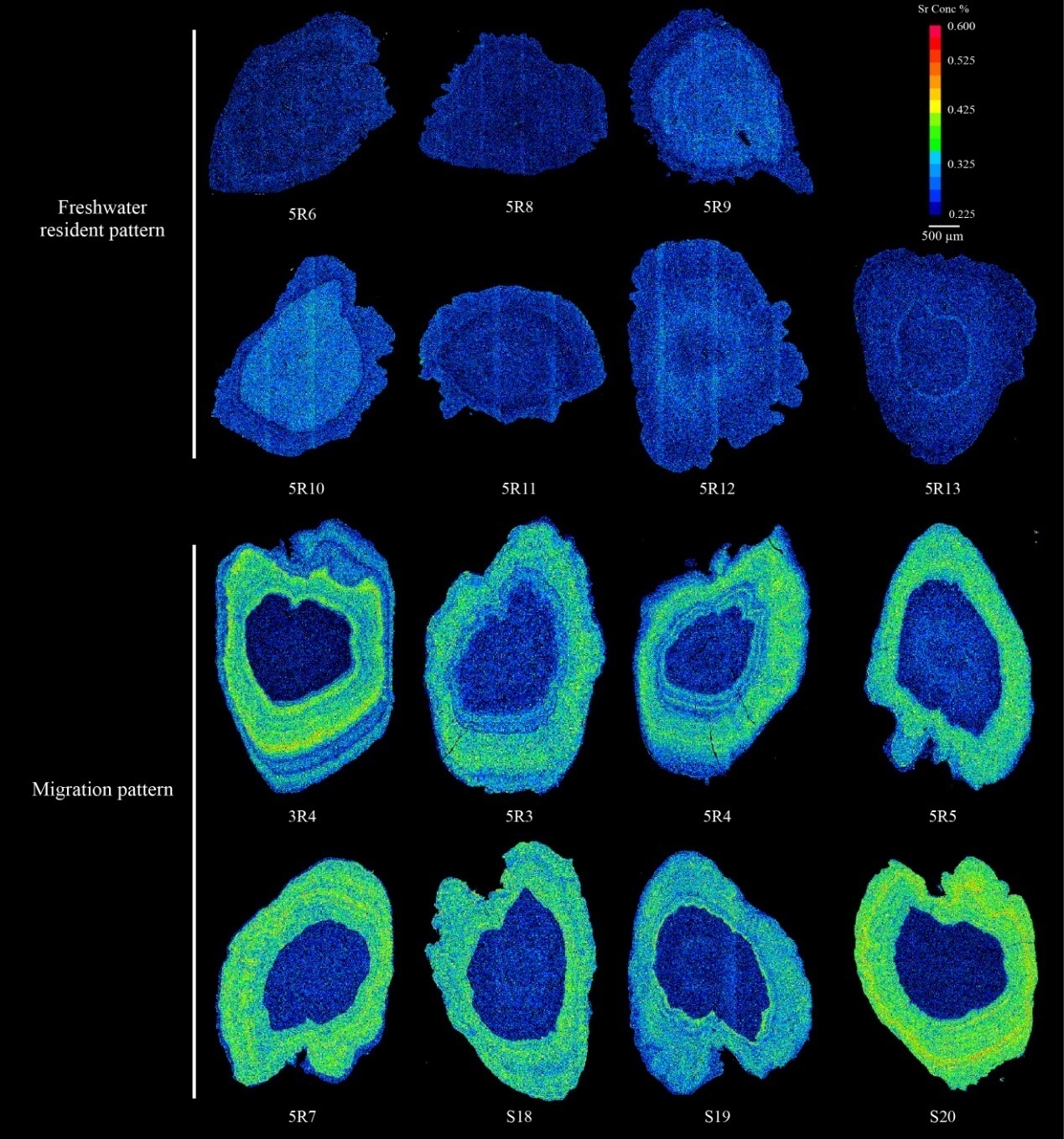


**Supplementary Figure 3.** X-ray intensity maps of Sr content in otoliths of *C. nasus*. The constant blue color represents the freshwater resident pattern, while the alternating blue and green colors indicate the migratory pattern.

**Supplementary Table 1.** Statistics of sequencing reads from Illumina Hiseq 2500 and Pacbio platforms

| **Sequencing platform** | **Insert size**  **(bp)** | **Read length**  **(bp)** | **Raw reads**  **(Gb)** | **Clean reads**  **(Gb)** |
| --- | --- | --- | --- | --- |
| Hiseq 2500 | 250 | 150 | 58.47 | 45.88 |
|  | 500 | 100 | 40.83 | 33.95 |
|  | 800 | 100 | 35.83 | 29.37 |
|  | 2,000 | 125 | 36.43 | 18.11 |
|  | 5,000 | 125 | 38.73 | 16.57 |
|  | 10,000 | 125 | 37.61 | 24.65 |
|  | 20,000 | 125 | 30.02 | 13.21 |
| Pacbio | 20,000 | 14,743 (average) | 68.62 | 67.77 |

**Supplementary Table 2.** Summary of the *k*-mer data

| **kmer** | **Kmer num** | **Kmerdepth** | **Genome size** | **Used bases** | **Used reads** | **×** |
| --- | --- | --- | --- | --- | --- | --- |
| 17 | 34,298,654,040 | 40 | 857,466,351 | 40,831,731,000 | 408,317,310 | 47.62 |

**Supplementary Table 3.** Detailed classifications of repeat sequences

| **Type of**  **Transposable elements** | **Parameter** | | |
| --- | --- | --- | --- |
|  | **Number** | **Base (bp)** | **% of the genome** |
| **DNA transponson** | | | |
| other | 204,646 | 41,634,437 | 4.86 |
| TcMar | 125,064 | 23,879,589 | 2.78 |
| PIF | 33,761 | 6,272,174 | 0.73 |
| Sola | 232,865 | 47,257,315 | 5.51 |
| Ginger | 153,840 | 24,514,560 | 2.86 |
| Kolobok | 50,845 | 8,931,277 | 1.04 |
| hAT | 338,959 | 48,464,320 | 5.65 |
| **LTR Retrotransposon** | | | |
| other | 144 | 150,961 | 0.02 |
| ERVK | 54,668 | 4,966,273 | 0.58 |
| ERV1 | 67,295 | 8,400,313 | 0.98 |
| Gypsy | 93,150 | 23,711,064 | 2.77 |
| Copia | 8,680 | 1,426,284 | 0.17 |
| **Non-LTR Retrotransposon** | | | |
| LINE/Penelope | 39,869 | 6,467,416 | 0.75 |
| LINE/L2 | 134,956 | 25,736,029 | 3.00 |
| LINE/L1 | 51,935 | 9,066,692 | 1.06 |
| SINE | 751 | 647,277 | 0.08 |
| tRNA | 9,987 | 1,297,530 | 0.15 |
| Simple_repeat | 248,976 | 49,329,916 | 5.75 |
| Unclassified | 93,244 | 25,167,777 | 2.94 |
| Total (Non-redundance) |  | 270,634,569 | 31.11 |

**Supplementary Table 4.** Summary of gene annotations

| **Evidence** | **Software/**  **Species** | | **Numbers** | | **Average transcript length (bp)** | **Average CDS length (bp)** | **Average exon per gene** | **Average exon length (bp)** | **Average intron length (bp)** |
| --- | --- | --- | --- | --- | --- | --- | --- | --- | --- |
| ***De novo*** | | AUGUSTUS | | 48197 | 9955.5 | 993.7 | 5.4 | 185.4 | 2056.2 |
| **transcriptome**  **Homolog**  **Glean** | | Cufflink | | 52328 | 7298.7 | 1601.0 | 5.1 | 312.4 | 1381.0 |
|  |  | *Danio rerio* | | 17069 | 25844.1 | 1542.9 | 9.1 | 169.4 | 2997.3 |
|  |  | *Gasterosteus aculeatus* | | 15418 | 17194.8 | 1487.4 | 9.1 | 163.8 | 1943.3 |
|  |  | *Oryzias latipes* | | 14234 | 16520.9 | 1463.0 | 9.0 | 162.8 | 1885.7 |
|  |  | *Takifugu rubripes* | | 14336 | 17770.9 | 1518.1 | 9.3 | 163.9 | 1967.7 |
|  |  | *Tetraodon nigroviridis* | | 17181 | 17709.5 | 1515.9 | 9.6 | 158.1 | 1886.1 |
|  |  |  | | 20837 | 16775.5 | 1759.7 | 10.1 | 173.9 | 1476.1 |

**Supplementary Table 5.** Summary of function annotations

| **Type** | **Number** | **Percentage (%)** |
| --- | --- | --- |
| Total | 20,837 |  |
| InterPro | 19,398 | 93.09 |
| KEGG | 18,313 | 87.89 |
| Swissprot | 19,398 | 93.09 |
| TrEMBL | 20,221 | 97.04 |
| Annotated | 20,300 | 97.42 |
| Unanotated | 537 | 2.58 |

**Supplementary Table 6.** Summary of marker number, genetic distance and physical length of each pseudochromosome

| Chr | First version | | |  | Second version | | |
| --- | --- | --- | --- | --- | --- | --- | --- |
| **Pseudo-**  **chromosome** | **Marker number** | **Genetic distance (cM)** | **Physical length (Mb)** |  | **Marker number** | **Genetic distance (cM)** | **Physical length (Mb)** |
| chr01 | 158 | 290.82 | 64.85 |  | 614 | 295.28 | 35.96 |
| chr02 | 46 | 107.77 | 10.20 |  | 604 | 174.89 | 35.62 |
| chr03 | 176 | 211.81 | 47.37 |  | 985 | 708.28 | 43.13 |
| chr04 | 188 | 232.79 | 40.10 |  | 613 | 647.61 | 23.63 |
| chr05 | 94 | 99.89 | 21.52 |  | 826 | 535.80 | 34.53 |
| chr06 | 191 | 133.79 | 44.27 |  | 627 | 267.35 | 32.83 |
| chr07 | 132 | 82.87 | 23.81 |  | 592 | 202.54 | 40.28 |
| chr08 | 195 | 262.90 | 32.66 |  | 432 | 320.05 | 35.79 |
| chr09 | 105 | 124.97 | 36.33 |  | 584 | 240.09 | 37.21 |
| chr10 | 162 | 204.54 | 16.58 |  | 303 | 292.68 | 27.83 |
| chr11 | 132 | 182.48 | 10.20 |  | 555 | 181.30 | 39.44 |
| chr12 | 140 | 180.73 | 26.43 |  | 434 | 190.50 | 37.58 |
| chr13 | 137 | 177.28 | 29.90 |  | 1,272 | 737.30 | 27.10 |
| chr14 | 83 | 116.14 | 18.55 |  | 884 | 269.58 | 35.69 |
| chr15 | 73 | 75.66 | 16.30 |  | 632 | 152.76 | 34.85 |
| chr16 | 107 | 178.07 | 34.59 |  | 251 | 103.51 | 33.87 |
| chr17 | 132 | 182.48 | 14.50 |  | 785 | 327.69 | 31.37 |
| chr18 | 190 | 225.29 | 34.21 |  | 722 | 271.41 | 31.85 |
| chr19 | 89 | 162.85 | 15.53 |  | 709 | 226.73 | 33.21 |
| chr20 | 120 | 158.79 | 30.52 |  | 594 | 172.55 | 30.97 |
| chr21 | 88 | 118.14 | 28.40 |  | 473 | 166.10 | 38.87 |
| chr22 | 82 | 186.97 | 20.52 |  | 394 | 176.00 | 31.37 |
| chr23 | 91 | 153.88 | 33.16 |  | 710 | 577.63 | 29.60 |
| chr24 | 77 | 137.59 | 32.78 |  | 705 | 413.32 | 29.55 |
| Total | 2,988 | 3,988.5 | 683.28 |  | 15,300 | 7,650.95 | 812.13 |

**Supplementary Table 7.** Statistics of the SOAPdenovo assembly of *C. nasus*

| Genome assembly |  |
| --- | --- |
| Contig N50 size (bp) | 19,022 |
| Contig number (>100bp) | 84,691 |
| Scaffold N50 size (Mb) | 1.8 |
| Scaffold number (>100bp) | 1,159 |
| Total length (Mb) | 786.3 |
| Genome coverage (X) | 389.2 |
| The longest scaffold (Mb) | 10.4 |
| Genome annotation |  |
| Protein-coding gene number | 20,353 |
| Mean transcript length (bp) | 19109.5 |
| Mean exons per gene | 9.0 |
| Mean exon length (bp) | 1695.0 |
| Mean intron length (bp) | 2177.9 |

**Supplementary Table 8.** Summary of map ratio for the 96 resequenced samples

| **Library** | **Sample** | **Mapped reads (bp)** | **Map ratio** |
| --- | --- | --- | --- |
| wHAXPI033721-13 | S12-MA | 45,489,893 | 64.99% |
| wHAXPI033722-15 | S13-MA | 45,316,593 | 64.74% |
| wHAXPI033723-16 | S14-MA | 45,283,722 | 64.69% |
| wHAXPI033724-17 | S15-MA | 45,371,518 | 64.82% |
| wHAXPI033725-18 | S16-MA | 45,455,201 | 64.94% |
| wHAXPI033726-19 | S17-MA | 45,642,777 | 65.20% |
| wHAXPI033727-20 | S18-MA | 45,571,972 | 65.10% |
| wHAXPI033728-21 | S19-MA | 45,136,703 | 64.48% |
| wHAXPI033729-22 | S20-MA | 46,097,741 | 65.85% |
| wHAXPI033730-23 | S21-MA | 47,387,120 | 67.70% |
| wHAXPI033731-24 | S22-MA | 45,892,701 | 65.56% |
| wHAXPI033732-25 | S23-MA | 45,692,067 | 65.26% |
| wHAXPI033733-26 | S24-MA | 45,692,068 | 65.27% |
| wHAXPI033734-27 | S25-MA | 45,940,919 | 65.63% |
| wHAXPI033735-28 | S26-MA | 45,531,842 | 65.05% |
| wHAXPI033736-29 | E1-MA | 46,162,881 | 65.95% |
| wHAXPI033737-30 | E2-MA | 45,984,957 | 65.69% |
| wHAXPI033738-31 | E3-MA | 45,770,595 | 65.39% |
| wHAXPI033739-32 | E4-MA | 45,823,290 | 65.46% |
| wHAXPI033740-34 | E5-MA | 45,943,251 | 65.63% |
| wHAXPI033741-35 | E6-MA | 45,943,250 | 65.62% |
| wHAXPI033742-36 | E7-MA | 45,770,594 | 65.39% |
| wHAXPI033743-39 | E8-MA | 46,432,831 | 66.33% |
| wHAXPI033744-40 | E9-MA | 46,197,973 | 66.00% |
| wHAXPI033745-41 | E10-MA | 46,816,560 | 66.88% |
| wHAXPI033746-42 | E11-MA | 46,370,074 | 66.24% |
| wHAXPI033747-45 | E12-MA | 46,432,830 | 66.33% |
| wHAXPI033748-47 | E13-MA | 46,845,498 | 66.92% |
| wHAXPI033749-50 | E14-MA | 46,517,873 | 66.45% |
| wHAXPI033834-54 | E15-MA | 45,869,671 | 65.53% |
| wHAXPI033835-58 | 2R-M1A | 45,956,463 | 65.65% |
| wHAXPI033836-59 | 2R-M2A | 45,972,226 | 65.67% |
| wHAXPI033837-61 | 2R-M3A | 46,233,495 | 66.05% |
| wHAXPI033838-63 | 2R-M4A | 46,113,473 | 65.88% |
| wHAXPI033839-68 | 2R-M5A | 46,168,622 | 65.96% |
| wHAXPI033840-72 | 2R-M6A | 46,168,255 | 65.95% |
| wHAXPI033841-73 | 2R-M7A | 45,959,668 | 65.66% |
| wHAXPI033842-74 | 2R-M8A | 46,212,508 | 66.02% |
| wHAXPI033843-75 | 2R-M9A | 46,499,937 | 66.43% |
| wHAXPI033844-78 | 2R-M10A | 46,493,302 | 66.42% |
| wHAXPI033845-80 | 2R-M11A | 46,195,125 | 65.99% |
| wHAXPI033846-81 | 2R-M12A | 46,233,313 | 66.05% |
| wHAXPI033847-84 | 2R-M13A | 46,038,983 | 65.77% |
| wHAXPI033848-87 | 2R-M14A | 46,039,662 | 65.77% |
| wHAXPI033849-88 | 2R-M15A | 46,227,763 | 66.04% |
| wHAXPI033850-89 | 3R-M1A | 47,060,434 | 67.23% |
| wHAXPI033851-92 | 3R-M2A | 46,952,941 | 67.08% |
| wHAXPI033852-93 | 3R-M3A | 46,970,317 | 67.10% |
| wHAXPI033853-94 | 3R-M4A | 46,906,336 | 67.01% |
| wHAXPI033854-95 | 3R-M5A | 46,893,059 | 66.99% |
| wHAXPI033855-98 | 3R-M6A | 47,069,390 | 67.24% |
| wHAXPI033856-101 | 3R-M7A | 46,840,023 | 66.91% |
| wHAXPI033858-108 | 3R-M9A | 47,222,760 | 67.46% |
| wHAXPI033859-111 | 3R-M10A | 47,304,204 | 67.58% |
| wHAXPI033860-112 | 3R-M11A | 47,595,383 | 67.99% |
| wHAXPI033861-133 | 3R-M12A | 44,040,138 | 62.91% |
| wHAXPI033865-142 | 4R-M3A | 46,839,267 | 66.91% |
| wHAXPI033866-17 | 4R-M7A | 47,035,757 | 67.19% |
| wHAXPI033867-13 | 4R-M4A | 47,047,091 | 67.21% |
| wHAXPI033869-140 | 4R-M1A | 47,042,671 | 67.20% |
| wHAXPI033870-18 | 4R-M8A | 46,986,051 | 67.12% |
| wHAXPI033874-15 | 4R-M5A | 47,137,864 | 67.34% |
| wHAXPI033877-16 | 4R-M6A | 46,958,856 | 67.08% |
| wHAXPI033881-19 | 4R-M9A | 46,874,029 | 66.96% |
| wHAXPI033916-20 | 4R-M10A | 46,647,208 | 66.64% |
| wHAXPI033917-21 | 4R-M11A | 46,507,581 | 66.44% |
| wHAXPI033918-22 | 4R-M13A | 49,498,495 | 70.71% |
| wHAXPI033919-23 | 4R-M14A | 44,134,359 | 63.05% |
| wHAXPI033920-24 | 4R-M15A | 44,761,673 | 63.95% |
| wHAXPI033921-25 | 5R-M1A | 44,306,458 | 63.29% |
| wHAXPI033922-26 | 5R-M2A | 44,802,586 | 64.00% |
| wHAXPI033923-27 | 5R-M3A | 44,971,013 | 64.24% |
| wHAXPI033924-28 | 5R-M4A | 47,335,676 | 67.62% |
| wHAXPI033925-29 | 5R-M5A | 47,530,765 | 67.90% |
| wHAXPI033926-30 | 5R-M6A | 49,412,007 | 70.59% |
| wHAXPI033927-31 | 5R-M7A | 47,478,783 | 67.83% |
| wHAXPI033928-32 | 5R-M8A | 49,419,671 | 70.60% |
| wHAXPI033929-34 | 5R-M9A | 49,452,201 | 70.65% |
| wHAXPI033930-35 | 5R-M10A | 49,569,443 | 70.81% |
| wHAXPI033931-36 | 5R-M11A | 48,564,785 | 69.38% |
| wHAXPI033932-39 | 5R-M12A | 48,272,482 | 68.96% |
| wHAXPI033933-40 | 5R-M13A | 48,017,430 | 68.60% |
| wHAXPI033934-41 | 6R-M1A | 45,657,949 | 65.23% |
| wHAXPI033935-42 | 6R-M2A | 47,782,865 | 68.26% |
| wHAXPI033965-45 | 6R-M3A | 47,506,428 | 67.87% |
| wHAXPI033966-47 | 6R-M4A | 47,488,711 | 67.84% |
| wHAXPI033967-50 | 6R-M5A | 47,601,083 | 68.00% |
| wHAXPI033968-54 | 6R-M6A | 47,628,709 | 68.04% |
| wHAXPI033969-58 | 6R-M7A | 45,901,915 | 65.57% |
| wHAXPI033970-59 | 6R-M8A | 45,585,547 | 65.12% |
| wHAXPI033971-61 | 6R-M9A | 45,553,050 | 65.08% |
| wHAXPI033972-63 | 6R-M10A | 46,071,540 | 65.82% |
| wHAXPI033973-68 | 6R-M11A | 48,173,855 | 68.82% |
| wHAXPI033974-72 | 6R-M12A | 48,032,919 | 68.62% |
| wHAXPI033975-73 | 6R-M13A | 48,072,171 | 68.67% |
| wHAXPI033976-74 | 6R-M15A | 47,710,969 | 68.16% |

**Supplementary Table 9.** Location sites of the genetic variants

| LG | Total | Intergenic | Intron |  | Exon |  |
| --- | --- | --- | --- | --- | --- | --- |
|  |  |  |  | **Total** | **Nonsynonymous** | **Synonymous** |
| LG1 | 4,043,744 | 2,619,792 | 1,312,804 | 111,148 | 62,970 | 48,178 |
| LG2 | 615,345 | 452,648 | 145,572 | 17,125 | 9,751 | 7,374 |
| LG3 | 2,867,517 | 1,768,701 | 1,016,347 | 82,469 | 43,487 | 38,982 |
| LG4 | 2,407,749 | 1,573,472 | 775,900 | 58,377 | 29,551 | 28,826 |
| LG5 | 1,253,663 | 812,571 | 411,534 | 29,558 | 15,193 | 14,365 |
| LG6 | 2,081,086 | 1,341,593 | 682,227 | 57,266 | 30,543 | 26,723 |
| LG7 | 1,610,353 | 1,063,562 | 508,395 | 38,396 | 20,694 | 17,702 |
| LG8 | 2,165,843 | 1,412,487 | 700,680 | 52,676 | 29,104 | 23,572 |
| LG9 | 2,298,646 | 1,431,203 | 811,539 | 55,904 | 29,729 | 26,175 |
| LG10 | 1,062,094 | 698,500 | 342,346 | 21,248 | 10,807 | 10,441 |
| LG11 | 184,686 | 113,609 | 65,372 | 5,705 | 3,175 | 2,530 |
| LG12 | 173,042 | 96,321 | 73,004 | 3,717 | 1,942 | 1,775 |
| LG13 | 1,905,934 | 1,226,808 | 628,890 | 50,236 | 27,571 | 22,665 |
| LG14 | 1,163,587 | 755,210 | 378,821 | 29,556 | 15,937 | 13,619 |
| LG15 | 653,728 | 464,444 | 173,953 | 15,331 | 8,106 | 7,225 |
| LG16 | 2,159,466 | 1,394,738 | 711,942 | 52,786 | 29,655 | 23,131 |
| LG17 | 886,957 | 601,386 | 265,422 | 20,149 | 10,969 | 9,180 |
| LG18 | 2,094,807 | 1,307,611 | 733,476 | 53,720 | 28,521 | 25,199 |
| LG19 | 948,289 | 600,656 | 320,716 | 26,917 | 15,038 | 11,879 |
| LG20 | 1,918,374 | 1,193,293 | 678,446 | 46,635 | 23,865 | 22,770 |
| LG21 | 1,703,664 | 1,057,757 | 602,904 | 43,003 | 22,350 | 20,653 |
| LG22 | 1,201,049 | 705,721 | 459,198 | 36,130 | 20,191 | 15,939 |
| LG23 | 2,013,862 | 1,244,294 | 712,699 | 56,869 | 29,071 | 27,798 |
| LG24 | 2,002,211 | 1,339,465 | 610,133 | 52,613 | 26,992 | 25,621 |
| Total | 39,415,696 | 25,275,842 | 13,122,320 | 1,017,534 | 545,212 | 472,322 |

**Supplementary Table 10.** List of the150 candidate genes

| Gene ID | Chromosome | Start | END | Gene name |
| --- | --- | --- | --- | --- |
| KN_GLEAN_10000913 | LG1 | 26238647 | 26262350 | Plakophilin-4 |
| KN_GLEAN_10000972 | LG1 | 527280 | 610150 | Obscurin |
| KN_GLEAN_10004101 | LG1 | 38006861 | 38007139 | ETS domain-containing transcription factor ERF |
| KN_GLEAN_10004102 | LG1 | 38009102 | 38009380 | ETS domain-containing transcription factor ERF |
| KN_GLEAN_10004103 | LG1 | 38014188 | 38016541 | ETS domain-containing transcription factor ERF |
| KN_GLEAN_10009227 | LG1 | 3196401 | 3224127 | BMP-binding endothelial regulator protein |
| KN_GLEAN_10015338 | LG1 | 27919754 | 27987627 | Oxidation resistance protein 1 |
| KN_GLEAN_10019668 | LG1 | 20483681 | 20490957 | Probable ATP-dependent RNA helicase DDX23 |
| KN_GLEAN_10020664 | LG1 | 31517515 | 31555285 | Acyl-coenzyme A oxidase-like protein |
| KN_GLEAN_10020696 | LG1 | 32629783 | 32674152 | Polypeptide N-acetylgalactosaminyltransferase 2 |
| KN_GLEAN_10020740 | LG1 | 48040179 | 48161837 | Filamin-B |
| KN_GLEAN_10020742 | LG1 | 48174076 | 48177756 | Sarcolemmal membrane-associated protein |
| KN_GLEAN_10020758 | LG1 | 48964284 | 48995194 | Prolyl 3-hydroxylase 2 |
| KN_GLEAN_10006490 | LG3 | 36301117 | 36362138 | Bicaudal D-related protein 1 |
| KN_GLEAN_10002020 | LG4 | 37791362 | 37865198 | Cadherin-16 |
| KN_GLEAN_10009288 | LG4 | 25661964 | 25671547 | Multiple C2 and transmembrane domain-containing protein 2 |
| KN_GLEAN_10009714 | LG4 | 29954352 | 30006693 | Ras GTPase-activating-like protein IQGAP1 |
| KN_GLEAN_10015286 | LG4 | 39429228 | 39480698 | Diacylglycerol kinase zeta |
| KN_GLEAN_10015320 | LG4 | 38475775 | 38503500 | Gamma-aminobutyric acid receptor subunit alpha-4 |
| KN_GLEAN_10016404 | LG4 | 33088981 | 33109508 | Protein CBFA2T3 |
| KN_GLEAN_10017298 | LG4 | 28533331 | 28598316 | CUGBP Elav-like family member 4 |
| KN_GLEAN_10017302 | LG4 | 28879912 | 28891394 | Trans-1,2-dihydrobenzene-1,2-diol dehydrogenase |
| KN_GLEAN_10019785 | LG4 | 24108906 | 24130459 | Solute carrier family 12 member 4 |
| KN_GLEAN_10019790 | LG4 | 23990544 | 24038124 | Nuclear factor of activated T-cells, cytoplasmic 3 |
| KN_GLEAN_10019800 | LG4 | 23656036 | 23777467 | Sodium/hydrogen exchanger 5 |
| KN_GLEAN_10023042 | LG4 | 21295794 | 21383422 | A disintegrin and metalloproteinase with thrombospondin motifs 17 |
| KN_GLEAN_10025421 | LG4 | 5230588 | 5254682 | Inactive phospholipase C-like protein 1 |
| KN_GLEAN_10025429 | LG4 | 5445995 | 5451861 | UBX domain-containing protein 4 |
| KN_GLEAN_10025464 | LG4 | 6314970 | 6380442 | Collagen alpha-2(V) chain |
| KN_GLEAN_10025465 | LG4 | 6388681 | 6389913 | Titin |
| KN_GLEAN_10025643 | LG4 | 13615366 | 13618435 | Nuclear receptor subfamily 4 group A member 2 |
| KN_GLEAN_10025650 | LG4 | 13812980 | 13815548 | Uridine phosphorylase 2 |
| KN_GLEAN_10025651 | LG4 | 13896736 | 13995347 | Plakophilin-4 |
| KN_GLEAN_10025663 | LG4 | 14481939 | 14543189 | Potassium voltage-gated channel subfamily H member 7 |
| KN_GLEAN_10004804 | LG5 | 12864794 | 12973980 | Receptor-type tyrosine-protein phosphatase gamma |
| KN_GLEAN_10004807 | LG5 | 12710675 | 12837472 | Calcium-dependent secretion activator 1 |
| KN_GLEAN_10009810 | LG6 | 38021701 | 38029710 | 3-ketodihydrosphingosine reductase |
| KN_GLEAN_10009813 | LG6 | 38091491 | 38151786 | Histone-lysine N-methyltransferase MLL3 |
| KN_GLEAN_10009815 | LG6 | 38284412 | 38345558 | Rho GTPase-activating protein 21 |
| KN_GLEAN_10011595 | LG6 | 27642405 | 27760003 | Voltage-dependent P/Q-type calcium channel subunit alpha-1A |
| KN_GLEAN_10007149 | LG7 | 3539980 | 3562211 | Target of Nesh-SH3 |
| KN_GLEAN_10008087 | LG7 | 501892 | 502641 | Transmembrane protein FAM155A |
| KN_GLEAN_10016720 | LG7 | 7457617 | 7489009 | Copine-7 |
| KN_GLEAN_10016743 | LG7 | 7081026 | 7094769 | Receptor-type tyrosine-protein phosphatase eta |
| KN_GLEAN_10017690 | LG7 | 19292717 | 19294555 | Mpv17-like protein 2 |
| KN_GLEAN_10017697 | LG7 | 18589026 | 18591237 | Aminoglycoside phosphotransferase domain-containing protein 1 |
| KN_GLEAN_10020514 | LG8 | 16155258 | 16178248 | TBC1 domain family member 20 |
| KN_GLEAN_10001731 | LG9 | 6184227 | 6187862 | Dermatan-sulfate epimerase-like protein |
| KN_GLEAN_10013515 | LG9 | 10944040 | 10987638 | Alpha-2-macroglobulin-like protein 1 |
| KN_GLEAN_10018306 | LG9 | 15306172 | 15393345 | Ubiquitin-protein ligase E3C |
| KN_GLEAN_10018315 | LG9 | 2839255 | 2839708 | Mannose-specific lectin |
| KN_GLEAN_10009884 | LG11 | 5457743 | 5502567 | Protein FAM190A |
| KN_GLEAN_10013268 | LG11 | 8772655 | 8817808 | Myomesin-3 |
| KN_GLEAN_10013302 | LG11 | 9931509 | 9975135 | Syntaxin-binding protein 5 |
| KN_GLEAN_10019226 | LG12 | 22345592 | 22394534 | Cytoplasmic polyadenylation element-binding protein 3 |
| KN_GLEAN_10022423 | LG12 | 8744917 | 8804011 | Sarcoplasmic/endoplasmic reticulum calcium ATPase 3 |
| KN_GLEAN_10022469 | LG12 | 7229368 | 7235247 | Transient receptor potential cation channel subfamily V member 1 |
| KN_GLEAN_10008751 | LG13 | 18874033 | 18877697 | Pre-mRNA-splicing factor RBM22 |
| KN_GLEAN_10008760 | LG13 | 19028682 | 19076137 | Platelet-derived growth factor receptor beta |
| KN_GLEAN_10011050 | LG13 | 26973457 | 27052122 | Protein jagged-2 |
| KN_GLEAN_10015659 | LG13 | 9449134 | 9519932 | Neurexin-3-alpha |
| KN_GLEAN_10016914 | LG13 | 23881864 | 23898960 | Homeobox protein Meis2 |
| KN_GLEAN_10017741 | LG13 | 16378399 | 16459453 | Tubulin polyglutamylase TTLL5 |
| KN_GLEAN_10017742 | LG13 | 16354728 | 16396342 | Pol polyprotein |
| KN_GLEAN_10006275 | LG14 | 10271575 | 10301616 | Paired amphipathic helix protein Sin3b |
| KN_GLEAN_10009772 | LG14 | 9834871 | 9870709 | Semaphorin-6B |
| KN_GLEAN_10001526 | LG15 | 2246231 | 2251813 | Methyl-CpG-binding domain protein 3 |
| KN_GLEAN_10007017 | LG15 | 339709 | 352711 | Protein APCDD1 |
| KN_GLEAN_10007018 | LG15 | 268383 | 335752 | RalA-binding protein 1 |
| KN_GLEAN_10010763 | LG15 | 3166379 | 3231398 | A disintegrin and metalloproteinase with thrombospondin motifs 16 |
| KN_GLEAN_10012466 | LG15 | 7691698 | 7694997 | Retrovirus-related Pol polyprotein from type-1 retrotransposable element R2 (Fragment) |
| KN_GLEAN_10012467 | LG15 | 7699756 | 7750058 | Vasoactive intestinal polypeptide receptor |
| KN_GLEAN_10021149 | LG15 | 5601383 | 5648372 | Adenylate cyclase type 1 |
| KN_GLEAN_10021165 | LG15 | 5957197 | 5961939 | Retinoid isomerohydrolase |
| KN_GLEAN_10021182 | LG15 | 6772358 | 6787368 | Epidermal growth factor receptor |
| KN_GLEAN_10021183 | LG15 | 6793350 | 6812686 | Niemann-Pick C1 protein |
| KN_GLEAN_10021214 | LG15 | 7602442 | 7605380 | Actin-binding protein IPP |
| KN_GLEAN_10024396 | LG15 | 12566438 | 12568051 | Growth hormone secretagogue receptor type 1 |
| KN_GLEAN_10004177 | LG16 | 3156069 | 3172707 | 26S proteasome non-ATPase regulatory subunit 12 |
| KN_GLEAN_10013190 | LG17 | 2948387 | 2958050 | Homocysteine-responsive endoplasmic reticulum-resident ubiquitin-like domain member 2 protein |
| KN_GLEAN_10001473 | LG18 | 22201156 | 22293584 | Glycine N-methyltransferase |
| KN_GLEAN_10008052 | LG18 | 585961 | 587357 | Peptidyl-prolyl cis-trans isomerase |
| KN_GLEAN_10008054 | LG18 | 517561 | 564171 | Tetratricopeptide repeat protein 33 |
| KN_GLEAN_10011739 | LG18 | 8102376 | 8122460 | Cytospin-A |
| KN_GLEAN_10020980 | LG18 | 26769166 | 26801505 | Minor histocompatibility protein HA-1 |
| KN_GLEAN_10020981 | LG18 | 26814233 | 26823986 | Protein strawberry notch homolog 2 |
| KN_GLEAN_10021050 | LG18 | 5821430 | 5825324 | RNA-binding protein 8A |
| KN_GLEAN_10021051 | LG18 | 5833230 | 5833649 | Type-4 ice-structuring protein LS-12 |
| KN_GLEAN_10021063 | LG18 | 6186466 | 6247906 | Raftlin |
| KN_GLEAN_10022638 | LG18 | 12589475 | 12681768 | Tensin-1 |
| KN_GLEAN_10005792 | LG19 | 14863712 | 14924000 | SH3 and multiple ankyrin repeat domains protein 1 |
| KN_GLEAN_10005795 | LG19 | 14931197 | 14937328 | BTB/POZ domain-containing protein 9 |
| KN_GLEAN_10005801 | LG19 | 15159905 | 15182138 | Otoferlin |
| KN_GLEAN_10019978 | LG19 | 10494757 | 10507799 | Zinc finger protein Rlf |
| KN_GLEAN_10020035 | LG19 | 11680378 | 11701623 | Vam6/Vps39-like protein |
| KN_GLEAN_10020036 | LG19 | 11710296 | 11735648 | Putative polypeptide N-acetylgalactosaminyltransferase-like protein 1 |
| KN_GLEAN_10007396 | LG20 | 27141497 | 27293463 | Protein sidekick-2 |
| KN_GLEAN_10007405 | LG20 | 27543565 | 27580416 | Putative all-trans-retinol 13,14-reductase |
| KN_GLEAN_10012288 | LG20 | 6947717 | 7062542 | Ryanodine receptor 2 |
| KN_GLEAN_10015531 | LG20 | 3722312 | 3863386 | Voltage-dependent T-type calcium channel subunit alpha-1G |
| KN_GLEAN_10015580 | LG20 | 4878377 | 4889387 | Protein archease |
| KN_GLEAN_10005945 | LG21 | 23143888 | 23196745 | Protein QN1 homolog |
| KN_GLEAN_10005946 | LG21 | 23146660 | 23147169 | Zinc finger BED domain-containing protein 1 |
| KN_GLEAN_10014470 | LG21 | 24228107 | 24389206 | Plexin A3 |
| KN_GLEAN_10023337 | LG21 | 5045798 | 5092174 | Protein FAM19A5 |
| KN_GLEAN_10013449 | LG22 | 10018810 | 10049513 | Sterol 26-hydroxylase, mitochondrial |
| KN_GLEAN_10014754 | LG22 | 5323071 | 5343323 | Intraflagellar transport protein 88 homolog |
| KN_GLEAN_10018097 | LG22 | 2246725 | 2291118 | Rho guanine nucleotide exchange factor 7 |
| KN_GLEAN_10018165 | LG22 | 4252897 | 4265204 | SH3 domain-binding protein 4 |
| KN_GLEAN_10018178 | LG22 | 4821685 | 4823106 | Ly6/PLAUR domain-containing protein 6B |
| KN_GLEAN_10019392 | LG22 | 13048015 | 13060572 | Protein FAM5C |
| KN_GLEAN_10019439 | LG22 | 13760758 | 13775967 | pre-mRNA 3' end processing protein WDR33 |
| KN_GLEAN_10019446 | LG22 | 14006949 | 14015079 | Transferrin receptor protein 1 |
| KN_GLEAN_10020093 | LG22 | 16225463 | 16226970 | Homeobox protein Hox-D3a |
| KN_GLEAN_10020098 | LG22 | 16383080 | 16385685 | Nuclear factor erythroid 2-related factor 2 |
| KN_GLEAN_10020100 | LG22 | 16459009 | 16510780 | Dual 3',5'-cyclic-AMP and -GMP phosphodiesterase 11A |
| KN_GLEAN_10020112 | LG22 | 16819530 | 16877661 | FERM, RhoGEF and pleckstrin domain-containing protein 1 |
| KN_GLEAN_10003836 | LG23 | 3316827 | 3318488 | Frizzled-1 |
| KN_GLEAN_10003837 | LG23 | 3378945 | 3410220 | Probable RNA-directed DNA polymerase from transposon BS |
| KN_GLEAN_10005052 | LG23 | 30977685 | 30986220 | Mothers against decapentaplegic homolog 4 |
| KN_GLEAN_10005053 | LG23 | 31004165 | 31057208 | Atrial natriuretic peptide receptor 1 |
| KN_GLEAN_10005072 | LG23 | 31436405 | 31436890 | GSK-3-binding protein |
| KN_GLEAN_10008950 | LG23 | 24367032 | 24415276 | Copine-4 |
| KN_GLEAN_10008979 | LG23 | 25279268 | 25279477 | Zinc finger protein 235 |
| KN_GLEAN_10011414 | LG23 | 32015694 | 32019406 | G protein-activated inward rectifier potassium channel 4 |
| KN_GLEAN_10011532 | LG23 | 12155263 | 12160447 | Lysophospholipase-like protein 1 |
| KN_GLEAN_10011601 | LG23 | 7678478 | 7679314 | Mediator of RNA polymerase II transcription subunit 18 |
| KN_GLEAN_10011602 | LG23 | 7663064 | 7670428 | Cytochrome P450 4B1 |
| KN_GLEAN_10011603 | LG23 | 7647407 | 7658389 | Nuclear inhibitor of protein phosphatase 1 |
| KN_GLEAN_10011621 | LG23 | 7128386 | 7182172 | Glutamate receptor, ionotropic kainate 3 |
| KN_GLEAN_10012192 | LG23 | 13613327 | 13669187 | Ceramide glucosyltransferase |
| KN_GLEAN_10012200 | LG23 | 13417602 | 13434272 | NADP-dependent malic enzyme |
| KN_GLEAN_10012891 | LG23 | 26838771 | 26857479 | TGF-beta receptor type-2 |
| KN_GLEAN_10012892 | LG23 | 26774171 | 26829610 | Dolichyl-diphosphooligosaccharide--protein glycosyltransferase subunit STT3B |
| KN_GLEAN_10012909 | LG23 | 26345194 | 26377714 | Tetratricopeptide repeat protein 35 |
| KN_GLEAN_10012920 | LG23 | 25500866 | 25507378 | Galanin receptor type 1 |
| KN_GLEAN_10012922 | LG23 | 25430624 | 25431452 | Myelin basic protein |
| KN_GLEAN_10014316 | LG23 | 28033790 | 28048135 | Nuclear receptor subfamily 2 group F member 5 |
| KN_GLEAN_10014364 | LG23 | 29211471 | 29266860 | Mitochondrial folate transporter/carrier |
| KN_GLEAN_10025109 | LG23 | 14373833 | 14390941 | Proton-coupled folate transporter |
| KN_GLEAN_10025392 | LG23 | 23662649 | 23776042 | Ral GTPase-activating protein subunit alpha-1 |
| KN_GLEAN_10003557 | LG24 | 27897254 | 27958902 | Sodium/calcium exchanger 1 |
| KN_GLEAN_10005931 | LG24 | 23179650 | 23201057 | ATP-binding cassette sub-family G member 4 |
| KN_GLEAN_10014560 | LG24 | 3624850 | 3641800 | Serrate RNA effector molecule homolog |
| KN_GLEAN_10016846 | LG24 | 32214973 | 32223673 | Serine/threonine-protein phosphatase 2A 65 kDa regulatory subunit A beta isoform (Fragment) |
| KN_GLEAN_10016847 | LG24 | 32242216 | 32244311 | Zona pellucida-like domain-containing protein 1 |
| KN_GLEAN_10017053 | LG24 | 27189945 | 27282816 | Pro-neuregulin-2, membrane-bound isoform |
| KN_GLEAN_10017611 | LG24 | 1511268 | 1529838 | RING finger protein 11 |
| KN_GLEAN_10024208 | LG24 | 18747234 | 18754261 | Protein YIPF5 |
| KN_GLEAN_10024209 | LG24 | 18767332 | 18769572 | Probable RNA-directed DNA polymerase from transposon X-element |

**Supplementary Table 11.** Go functions of the 150 candidate genes (see the separate excel file)

**Supplementary Table 12.** Pathway enrichments of the 150 candidate genes

| Pathway | Group No.  (134) | Gene No.  (20372) | *P*-value | Pathway ID | Gene ID |
| --- | --- | --- | --- | --- | --- |
| Glutamatergic synapse | 7 | 208 | 0.000459 | ko04724 | KN_GLEAN_10004101;KN_GLEAN_10004102;KN_GLEAN_10004103;KN_GLEAN_10005792;KN_GLEAN_10011621;KN_GLEAN_10021149;KN_GLEAN_10011595 |
| Calcium signaling pathway | 8 | 339 | 0.001838 | ko04020 | KN_GLEAN_10008760;KN_GLEAN_10012288;KN_GLEAN_10022423;KN_GLEAN_10021149;KN_GLEAN_10021182;KN_GLEAN_10015531;KN_GLEAN_10003557;KN_GLEAN_10011595 |
| Dilated cardiomyopathy | 9 | 455 | 0.003191 | ko05414 | KN_GLEAN_10013268;KN_GLEAN_10012288;KN_GLEAN_10025464;KN_GLEAN_10025465;KN_GLEAN_10022423;KN_GLEAN_10009227;KN_GLEAN_10021149;KN_GLEAN_10003557;KN_GLEAN_10015659 |
| Hypertrophic cardiomyopathy (HCM) | 8 | 414 | 0.006142 | ko05410 | KN_GLEAN_10013268;KN_GLEAN_10012288;KN_GLEAN_10025464;KN_GLEAN_10025465;KN_GLEAN_10022423;KN_GLEAN_10009227;KN_GLEAN_10003557;KN_GLEAN_10015659 |
| Adherens junction | 6 | 269 | 0.00883 | ko04520 | KN_GLEAN_10005052;KN_GLEAN_10025651;KN_GLEAN_10021182;KN_GLEAN_10016743;KN_GLEAN_10000913;KN_GLEAN_10012891 |
| Pancreatic cancer | 4 | 134 | 0.011908 | ko05212 | KN_GLEAN_10005052;KN_GLEAN_10007018;KN_GLEAN_10021182;KN_GLEAN_10012891 |
| Mucin type O-Glycan biosynthesis | 2 | 32 | 0.018724 | ko00512 | KN_GLEAN_10020696;KN_GLEAN_10020036 |
| PPAR signaling pathway | 3 | 102 | 0.029813 | ko03320 | KN_GLEAN_10020664;KN_GLEAN_10013449;KN_GLEAN_10012200 |
| Arrhythmogenic right ventricular cardiomyopathy (ARVC) | 6 | 376 | 0.038198 | ko05412 | KN_GLEAN_10012288;KN_GLEAN_10025464;KN_GLEAN_10022423;KN_GLEAN_10009227;KN_GLEAN_10003557;KN_GLEAN_10015659 |
| MAPK signaling pathway | 6 | 382 | 0.040739 | ko04010 | KN_GLEAN_10008760;KN_GLEAN_10020740;KN_GLEAN_10021182;KN_GLEAN_10015531;KN_GLEAN_10012891;KN_GLEAN_10011595 |
| Drug metabolism - other enzymes | 2 | 52 | 0.045986 | ko00983 | KN_GLEAN_10025650;KN_GLEAN_10009815 |

**Supplementary Table 13.** Summary of the 277-non-synonymous SNPs in the CDS regions of the 14 Ca2+-related genes (see the separate excel file)

**Supplementary Table 14.** Differential expression genes between the migratory group and the freshwater resident group (See the separate excel files).

**Supplementary Table 15.** Sequences of the primer pairs for quantitative RT-PCRs

| **Gene** | **Primer** | **Sequence (5’-3’)** |
| --- | --- | --- |
| *Smad4* | 182S | GGAGTGGACGACCTACGACG |
|  | 182A | GGTGTGCTTGATGCTCTGCC |
| *Frat* | 184S | ACACAACAATAGCGCAAACCAAA |
|  | 184A | GAAGGCGAATACAGCACCCG |
| *Cacnalg* | 202S | GCGGGATGGTTACTCGGACA |
|  | 202A | CGCTTCATCGGGCTGTTGTC |
| *Fzd1* | 189S | AGAGGATGCGGGACTGGAGG |
|  | 189A | CGCATACATGGAGCAGAGAAAGA |
| *Tgfbr2* | 195S | ATGTGACCATCCCGAGCAGG |
|  | 195A | TTGCACTCCTCCTGGTTGCA |
| *Slc8a1* | 197S | CTGCTGTCCGTCATCGAGGT |
|  | 197A | TAGGCGAAGATGCTCCAGGC |

S: forward; A: reverse.
