# Supplementary material for: Genome and population sequencing of a chromosome-level genome assembly of the Chinese tapertail anchovy (Coilia nasus) provides novel insights into migratory adaptation
Source: Gigascience. 2020 Jan 2;9(1):giz157. doi: 10.1093/gigascience/giz157 (PMC6939831; doi:10.1093/gigascience/giz157)
Supplement: giz157_Response_to_Reviewer_Comments_Original_Submission [file giz157_response_to_reviewer_comments_original_submission.pdf]

Author's Response To Reviewer Comments

Close

Reviewer #1:
General comments
The authors have sequenced and assembled the Chinese tapertail anchovy (Coilia nasus). The Chinese tapertail anchovy is intriguing because it has both migratory and non-migratory populations, facilitating investigation into the genetic basis for migration.
By sequencing 96 individuals (85 migratory and 11 non-migratory), the authors find 150 genes that have vastly different alleles between the migratory and non-migratory ecotypes.
There are multiple instances where the language is unclear or wrong. I would strongly advice going through the text carefully to find these places, maybe by a native English-speaking person. I have mentioned some places, but I would like to focus on the science and not the language.
Answer: Thanks for your advice. Yes, with kind assistance from the editor and ISE (a professional English editing company), we made a thorough revision of our manuscript, especially paid much attention to those places where you mentioned for changes.

Specific comments
Title: I prefer "population sequencing" rather than "resequencing", but that is a personal preference and not a recommendation for a change.
Answer: Thanks for your advice. Yes, we changed “resequencing” to “population sequencing”.

Abstract: The 'Results' part is a bit heavy to read. You could spilt the first to sentences to get a better flow.
Answer: Thanks for your advice. Yes, we split this sentence into two on lines 35-38.

Line 60: "of" is not necessary.
Line 63: "molecular mechanisms have still been unknown" should be "molecular mechanisms are still unknown".
Answer: Thanks for your comments. Yes, we made corresponding changes on lines 58 and 61.

Line 69: Does the cited article actually discuss the fish' "nutritional content and delicate flavor"? Anyhow, I am uncertain of the validity of having this kind of statements in a scientific paper. People would not buy food that tastes bad, and over time, would not buy food without enough nutrition.
Answer: Thanks for your comments. We removed this sentence and the cited article on line 67.

Line 76: I don't think the approximation symbol is used as this usually.
Answer: We changed ‘3~4 more months’ to ‘3 to 4 months’ on lines 73-74.

Line 76-77: Could be written better.
Answer: Yes, it is done on lines 73-75.

Line 78: "always resident" and "their whole lifetime" means the same.
Answer: We removed ‘always’ in this sentence on line 75.

Line 87: RAD actually used for creating the assembly? Rewrite, the important part is that you created a linkage map and used that, not the RAD itself.
Answer: Sorry for the misleading description. According to your advice, we rewrote this sentence as follows on lines 83-86.
Thus, in this study, we firstly produced the chromosome-level genome assembly of C. nasus, on basis of the genetic linkage map constructed with the digest restriction-site associated DNA (RAD) sequencing.

Line 90: What does "high-evidence for examination of the detailed molecular" mean?
Answer: Thanks for your question. We revised part of this sentence as follows on lines 87-89: we identified numerous SNPs (single nucleotide polymorphisms) to detect the molecular clues for adaptive

mechanisms between the migratory and freshwater residential groups.

Line 92-93: Could be written better.
Answer: Yes, it is done on lines 88-90. We rewrote it as follows.
The identified candidate genes for migratory adaptation will provide valuable resources for genetic research on fish migration.

Line 104: You could specify that this was using the actinopterygii dataset.
Answer: Thanks for your advice. Yes, we add “with actinopterygii_odb9 orthologues” in this sentence on line 104.

Line 105: "A BUSCO evaluation of our assembly was 90.1%," does not make sense. Please rewrite.
Answer: Thanks for your question. Yes, we revised this sentence as follows on lines 105-107.
The assessment result of our assembly was 90.1%, where C=87.1% [D=4.6%], F=3.0%, M=9.9%, and n=4584 (C: complete [D: duplicated], F: fragmented, M: missed, n: number of genes), thereby suggesting a high level of completeness for the C. nasus assembly

Line 125-126: Is this normal? Seems a bit low.
Answer: Yes, a mapping ratio over 60% is normal in a population sequencing.

Lines 128-130: You cannot call 25 million SNPs for 25 Mb SNPs. You could say 25 M SNPs however.
Answer: Yes, you are right. We changed ‘Mb’ to ‘M’ on lines 129-133.

Line 134: The "SNP set" is all 39 M SNPs? Or is it a reduced set? How many SNPs in that case?
Answer: Yes, the tree was constructed using the entire 39 M SNP set.

Line 136: Why so few non-migratory? Would it not be better comparing two groups of equal size than this large difference?
Answer: Thanks for your comments. Theoretically, it would be more accurate to collect various individuals with the same quantity. However, the freshwater residents were extremely rare and difficult for collection. In the present study, only 11 non-migratory samples were available after validation. However, we will try to collect more samples in different water areas.

Lines 158-159: "suggesting that the migratory adaptation may require a preference for selected genes in adjacent locations". What does this mean? How can you tell if this is a real phenomenon?
Answer: Sorry for this misleading description. We changed it to the following sentence on lines 155-156.
These genes had potentially undergone independent selection for involvement in migratory adaptation.

Line 171: I do not think you can say that "genes were clustered in terms of [..] physical (chromosomal) positions" if your example in line 162 is typical. 3 genes spread across 5 Mbp is not a lot. Further, how can you say that the genes are "clustered [..] in [..] physiological functions"? Would not this be expected? A migratory behavior would need some triggers, which some of the GO pathways might be, and would need some neurological connection. I do not find this surprising. Is as expected, I would say.
Answer: Thanks for your comments. We changed ‘the migratory adaptation-related genes were clustered in terms of both physical (chromosomal) positions and physiological function ‘to ‘these gene terms could be related to the migratory adaptation’ on line 173.

Section "Differentially expressed genes (DEGs) in the Ca2+-related pathways for the migrator group": If these genes are always more expressed in migratory fish than non-migratory fish, how can you tell if they are actually connected to migration? Would not the expectation be that something creates a cascade and up-regulates a bunch of genes, which then make the fish do the migration. If the genes are up-regulated the whole time, how would that contribution to the migration? When were the analysis done? I see from the section called "Genetic adaptation to complex environments during migration" that you say that these might be needed for the migratory fish to handle freshwater, which seems logical.
Answer: Thanks for your advice. It is indeed that many genes have higher expression levels in migratory fishes than in non-migratory individuals, which could be difficult to identify the real relationships for migration. However, in this DEG term, we mainly focused on the genes with non-synonymous SNPs. We thus speculated that these genomic variants potentially lead to the changes of transcription, and further influence the migration ability.

Line 303: Has the SOAPdenovo assembly been used for anything? That is not clear to me.

Answer: Thanks for your question. In fact, the SOAPdenovo assembly was used for SNP calling of population sequencing. Please find more details on lines 390-391.

Line 314: Why did you generate a Platanus assembly if you had a SOAPdenovo assembly already?
Answer: Thanks for the question. In fact, we independently run SOAPdenovo and Platanus. However, the Platanus data were more suitable for the combined assembling of Illumina and PacBio sequencing reads.

Line 332: Which assembly was annotated?
Answer: Both assemblies were used for genome annotation, independently. However, the results were combined at the end of annotation.

Line 408: Why have you used both assemblies in your analysis? You should in that case report the statistics and BUSCO scores for the SOAPdenovo assembly in the results, so that we as readers can assess its quality better.
Answer: Thanks for your advice. We added the BUSCO results of our SOAPdenovo assembly. Please find the details of two BUSCO scores on lines 106-108 and 316-318, respectively.

Section "Availability of supporting data": I could not find any of the genome assemblies on the two provided project IDs. Please make sure that they are released.
Answer: Thanks for your comments. Yes, the SOAPdenovo assembly was released under the project ID PRJNA421870, and the Pacbio assembly was uploaded to Gigascience FTP. Please find more details on lines 485.

Figure 2: Why do you have that particular order of the chromosomes? Why not 1,2,3,4 etc? What is the naming of the chromosomes based on? It is common to call the largest sequence for chromosome 1, second largest for chromosome 2 and so on. This does not seem to be the case here.
Answer: Thanks for your good question. In our present study, however, we did not use the chromosome size as the key factor to name the chromosomes. In fact, we named these chromosomes based on our newly constructed genetic linkage map. For example, the chromosome assembled from LG1 was named as Chr1. Thus, the size of Chr1 is not the largest. Please find more details of each chromosome in Supplementary Table 6.

Supporting data: Please provide the SOAPdenovo assembly here also.

Answer: Thanks for your advice. Yes, we added a brief table, Supplementary Table 7, to provide statistics of the SOAPdenovo assembly.

Reviewer #2:
The article reports the chromosome-wide genome assembly of the Chinese tapertail anchovy, and its respective use for population genomics studies. The genomic tools and data generated are of high relevance, as demonstrated here by its usefulness to address a biological question, the evolutionary aspects of migratory dimorphism.

The major weakness of the manuscript is the overall description inaccuracy in the methods and results sections, as listed below:
* Results presented without any respective methodology description, for example synteny blocks in Figure 2, enrichment pathway analysis for 150 candidate genes, BUSCO assessment (guessed the actinopterygii dataset was used by the number of gene models), and DEGs Heatmap in Figure 5g.

Answer: Thanks for your advice. Yes, we added more detailed method descriptions for the sections of synteny blocks (lines 406-409), enrichment pathway analysis (lines 432-434 & 448-449), BUSCO analysis (lines 102-103) and DEGs Heatmap (lines 455-458).

* Regarding population structure the results presented are solely a phylogenetic tree figure without presenting consolidated statistical values. Additionally confirmed by otoliths microstructure results, but no value is presented (eg . correlation). In the respective methods part is mentioned "PLINK was applied to calculate the genetic distance between individuals,..". What was the actual analysis procedure done with PLINK?

Answer: Thanks for your questions and advice. In fact, the phylogenetic tree were constructed by SNPs, thereby it does not have any statistical numbers. For the otoliths microstructure results in Figure 1, we demonstrated the scale bar of Sr concentrate. Based on Fig 1c, we could determine that the Sr concentrate in freshwater resident fishes was around 0.2%,whlie in migratory fishes it was around 0.4%. These differences could distinguish the two groups of fishes. The PLINK with parameters "--distance 1-ibs flat-missing" was used to calculate the genetic distances among individuals. Please find more details of the corresponding procedure on lines 419-424.

* Results mixed in the methods section (eg. lines 280-282; line 286-287; 302; 315-316; 319-320; 326-327; 329-331; and 399-406).

Answer: Sorry for the mixture of methods and results. We removed these sentences from the Methods section.

* Resequencing methodology for the population genomic study mentioned pooled samples but supplementary file sequencing libraries are for each 96 individual samples.

Answer: Sorry for the mistake. We changed it to "Population genome sequencing library (average insert size of about 350 bp) of each individual was independently constructed for DNAs from the 96 individuals" on lines 296-298.

* Methodologies in general are omissive regarding details necessary for reproducibility purpose or to understand the results reported, such as DNA amount used for libraries purposes, sampled tissues for qPCR, t-test assumption of data normality and equal variance, lack of the used software naming (line 306: "total reads were mapped onto the contigs") and/or respective parameters (if default was used state it).

Answer: Thanks for your advice. Yes, we added the detailed DNA amount extracted from denovo (about 90 μg) and population sequencing individuals. Please find more details on lines 282 and 294.
The sampled tissue for qRT-PCR is muscle (lines 450-452). For the t-test assumption, we changed this sentence to be 'Normal distribution and homogeneity of variance of data was tested with the Shapiro-Wilk and Levene tests (α = 0.05), respectively. Then differences in the mRNA levels were compared by the students' t-test using IBM SPSS Statistics 22.0 (IBM Inc., Chicago, IL, USA). P values of < 0.05 were considered statistically significant'. Please find more details on lines 459-469.
The sentence "total reads were mapped onto the contigs" was changed to "total reads were mapped onto the contigs by the third step of SOAPdenovo with default parameters" on lines 310-313.

* One example of methodology description not being accurate is the RNAseq expression analysis (line431-440). No description to the raw data editing and quality control filtering procedure. The mapping procedure solely names the HISAT2 without any parameters used specified. The usage of Cufflinks 2.2.1 suite of tools description is confusing; solely mentioning the Cufflinks program itself (which assembles the mapped reads into transcriptomes) and Cuffdiff to identify DEGs. No parameters of the programs are described, and no reference to the intermediate softwares in the workflow is done, such as Cuffcompare, Cuffmerge, CuffQuant and downstream the Cuffnorm. Presuming the usage of Cuffnorm to normalize the expression values across samples required for the heatmap presented in Figure 5g.

Answer: Thanks for your advice and comments. We added information of the detailed filter step of transcriptome reads on lines 446-449 as follows:
Raw data produced from the sequencing platform were filtered by removing reads contaminated with adaptors, more than 10% of N bases and more than 50% of low-quality bases (base quality score ≤ 10).

We also added the detailed parameters "--phred33 --sensitive --no-discordant --no-mixed -I 1 -X 1000" for HISAT2 on lines 450-451.
More details about 'The Cuffdiff in Cufflink package with parameters "-FDR 0.05 --geometric-norm TRUE –c 10" was added 'to identified the significantly differentially expressed genes (DEGs)' on lines 453-455.
For the Heatmap, edgeR software was used to normalize the expression values and transform the values to log2 values. Please find more details on lines 445-448.

* I'm not against the use of personal pronouns in scientific manuscript writing, but the extensive use of "We" in the results and in methods sections should be avoided, because doing so removes objectivity

required in these sections.

Answer: Thanks for your advice. The “We” manner in our manuscript was reduced in the methods section.

Without having addressed these main points listed above, is difficult to evaluate the fullness and implications of the results presented. Therefore, any other revision queries are not relevant until methods & results sections are revised. As exception, just to raise one point regarding one particular analysis and respective conclusion. This is about the identification of the 150 candidate genes related to the migratory adaptation, solely based on the colocalization with selective sweep regions. These genomic signatures of divergence don't necessarily have a cis effect on the neighbouring gene, as many have a functional trans effect on other loci further away in the linearity of the DNA. If you wanted to focus on the functional gene unit, why didn't you complemented it with the Non-synonymous/Synonymous mutation ratio (Ka/Ks) analysis of those genes, since you identified those coding region located SNPs?

Answer: Thanks for your advice. However, the Ka/Ks analysis is more available for the gene comparisons among diverse species, although there are some but too little diverse nuclear sites in coding regions for a Ka/Ks analysis in the same species. Fst and selective sweeping analysis were widely used in population sequencing or resequencing studies. We mainly focused on those genes with non-synonymous SNPs. We thus speculated that these genomic variants potentially led to changes of expression values, and further influenced the migration ability.

To summarise it, the genomic tools, the data generated and the biological question addressed are of high relevance, but hampered by the manuscript methods and results reporting.

Answer: Thanks for your nice advice. We added more detailed method descriptions according to your suggestions in the revised manuscript.

Close
