# Supplementary material for: Genome and population sequencing of a chromosome-level genome assembly of the Chinese tapertail anchovy (Coilia nasus) provides novel insights into migratory adaptation
Source: Gigascience. 2020 Jan 2;9(1):giz157. doi: 10.1093/gigascience/giz157 (PMC6939831; doi:10.1093/gigascience/giz157)
Supplement: giz157_Response_to_Reviewer_Comments_Revision_1 [file giz157_response_to_reviewer_comments_revision_1.pdf]

Author's Response To Reviewer Comments

Close

Reviewer #1:

Reviewer report.
Title: Genome sequencing and resequencing for a chromosome-level genome assembly of Chinese tapertail anchovy (Coilia nasus) to provide novel insights into migratory adaptation
General comments
I thank the authors for responding to mine and the other reviewer's comments. Overall, the manuscript reads much better now.
However, there are still some unresolved issues.

Specific comments
Line 126: You might have misunderstood me here. This low mapping ratios need to be explained. I usually see mapping ratios of more than 80 %. For instance, in a population sequencing project on cichlids, 96-98 % of the reads could be aligned to the reference: https://static-content.springer.com/esm/art%3A10.1038%2Fs41559-018-0717-x/MediaObjects/41559_2018_717_MOESM1_ESM.pdf. In another project on sea bream, more than 90 % of the reads align on average: https://www.nature.com/articles/s41598-019-42988-z. I do not understand why you have such a low alignment rate. It makes the data suspect.

Answer: Thanks for your detailed comments. In fact, these low alignment ratios may be caused by our relatively strict parameters of the alignment software (BWA with the following parameters: aln -n 0.04 -o 1 -e 30 -i 15 -d 10 -l 35 -k 2 -m 2000000 -t 4 -M 3 -O 11 -E 4 -R 30 -q 0 -I –f; added to the section of Materials and Methods on lines 391-394). On the other hand, we chose the formula (aligned reads/original reads) to calculate the mapping ratios. If we use the formula (aligned reads/filtered reads; for the majority of reported studies), these mapping ratios will increase about 5%. We mentioned the applied formula of aligned reads/original reads on lines 128-129 of the revised manuscript.

The Platanus genome assembly seems better than the SOAPdenovo one. Why did you not use the Platanus assembly for the population studies?

Answer: Thanks for your good question. You are right. The scaffold and contig N50 values of platanus and DBG2OLC (combined assembling of Illumina and PacBio sequencing reads) are usually better than the SOAPdenovo assembly (assembling of the Illumina reads). However, there is an inevitable defect for Pacbio reads in the former assembly method, since the error rate of Pacbio reads would be much higher than that of Illumina reads. Even if the Pacbio reads were undergone a series of filtering and correcting steps. We therefore chose the SOAPdenovo assembly as reference to increase the quality of SNPs.

Regarding the annotation: When I asked which assemblies were annotated, you answer both and that the "results were combined at the end of annotation". What does that mean? You cannot combine annotations in that way.

Answer: Sorry for the misleading description. In fact, we separately annotated the SOAPdenovo version and the combined version of platanus and DBG2OLC. For the final genome assembly, the combined version of platanus and DBG2OLC was applied (Table 2). Please find more details on lines 99-102 of the revised manuscript. However, we also provided the SOAPdenovo annotation (for high-quality SNPs in resequencing; Supplementary Table 7).
By the way, we did not combine both annotations for the genome assemblies due to the fact that the combined annotations would be seriously redundant. We are sorry for the previous misleading answer.

Reviewer #2:

The main concern about methods and results reporting format in the previous revision was properly addressed and most doubts properly answered. The minor details that now require attention are listed below:
The results subsection "Sequencing and assembly of the chromosome-level genome" should be named "Sequencing, assembly, and annotation of the chromosome-level genome"

Answer: Thanks for your advice. Yes, it is done on line 94.

The supplementary Material file is called "Overall opinion.docx".

Answer: Yes, it is done.

Line 98-100: Still not clear which assembly you are describing.

Answer: Thanks for your question. We rewrote this sentence as follows on lines 99-102.
“After removal of low-quality raw reads, we assembled a high-quality genome by using the combination of Platanus and DBG2OLC results with a scaffold N50 and a contig N50 of 2.1 Mb and 1.6 Mb, respectively”.
That is to say, for the final genome assembly, the combined version of platanus and DBG2OLC was applied. More details were also provided on lines 323-328 under the section of Materials and Methods.

Line 180-182: In "Supplementary Table 13. Summary of the 277-non-synonymous SNPs in the CDS regions of the 14 Ca2+-related genes (see the separate excel file)", only 45 SNPs are described and no significant statistical value is presented.

Answer: Sorry for this misleading descriptions. In fact, there are 45 non-synonymous SNPs with significantly statistical values in the CDS regions. We rewrote this sentence as follows (on lines 183-185).
In total, 45 non-synonymous SNPs were distributed in the coding sequence regions, and their allele frequencies were significantly different (P-value < 0.01, Fisher’s exact test) in the two fish groups (Supplementary Table 13).
By the way, we also employed the fisher test from R package to calculate the P-values that were listed in the Supplementary Table 13.

Line 184: (Figure 5a-f)

Answer: It was corrected on line 187.

Line 182 -189: No statistical values/significance presented in text or figure 5.

Answer: Thanks for your advice. We added the thresholds of fold change and P-value on lines 188-195 as follows.
Our results demonstrated that Smad4 (Figure 5a), Gbp (Figure 5b), Fzd1 (Figure 5d), Tgfbr2 (Figures 3b, 3c, and 5e), and Slc8a1 (Figure 5f) were transcribed more in the liver of the migratory group than that of freshwater residents (fold >2, P-value <0.05, t-test). Moreover, Cacnalg had a higher transcription level (fold >2, P-value <0.05, t-test; Figure 5c) in the heart of the migratory group than the freshwater residents. We also compared the transcription values in brain tissues of the migratory and resident groups, and 648 DEGs were identified (P-value <0.05 and fold >2). The detailed DEG IDs and their transcription values were provided in Supplementary Table 14.

Figure 5g: Gene name instead of your internal gene ID is more informative.

Answer: Thanks for your nice advice. Yes, it is done in the revised Fig 5g.

Supplementary Table 14. Describe the expression values presented.

Answer: Thanks for your advice. Yes, we add the following sentence on lines 194-195.
The detailed DEG IDs and their transcription values were provided in Supplementary Table 14.

Line 285: Missing kit and sequence platform name/references.

Answer: Thanks for the advice. Yes, we added related information in the following sentence on lines 286-289.
Three short-insert libraries (250, 500, and 800 bp) and four long-insert libraries (2, 5, 10, and 20 kb) were constructed using Illumina reagents (Illumina, San Diego, CA, USA) in accordance with the manufacturer's instructions.

Line 362-363: Don't understand this sentence: "The sorted and integrated 363 steps were performed in these alignments."

Answer: Sorry for this mistake. We rewrote this sentence as follows (on lines 365-366).
These alignments were sorted by using the samtools software (version 1.2, RRID:SCR_002105) [56].

Line 388: "SNP calling." Is it a subsection title?

Answer: Yes, you are right. Its format is similar to the above paragraph.

Line 445-448: Which software was used for quality control and editing of the raw reads?

Answer: Thanks for your question and advice. We add more details on lines 414-416 as follows.
SOAPfilter (version 2.2), a package from the SOAPdenovo2 (version 2.04.4; RRID:SCR_014986), was used to filter the population sequencing reads with adapters, low quality, undersize inserts, or PCR duplicates.

Line 452-457: Cuffdiff was used for DEG analysis and EdgeR to "draw the heatmap"? Do you mean you used EdgeR to normalize expression across samples required to draw the heatmap? If you used Cuffdiff why didn't you use Cuffnorm/CummRbund to output the heatmaps?

Answer: Thanks for your questions. Yes, we employed the EdgeR to draw the heatmap. In fact, we have done so in our previous reports, such as for mudskipper [1] and arowana [2] genome studies.
[1] You X, Bian C, Zan Q, Xu X, Liu X, Chen J, Wang J, Qiu Y, Li W, Zhang X et al: Mudskipper genomes provide insights into the terrestrial adaptation of amphibious fishes. Nature communications 2014, 5:5594.
[2] Bian C, Hu Y, Ravi V, Kuznetsova IS, Shen X, Mu X, Sun Y, You X, Li J, Li X et al: The Asian arowana (Scleropages formosus) genome provides new insights into the evolution of an early lineage of teleosts. Scientific reports 2016, 6:24501.

The main question which was not properly addressed from the previous revision: "about the identification of the 150 candidate genes related to the migratory adaptation, solely based on the colocalization with selective sweep regions. These genomic signatures of divergence don't necessarily have a cis effect on the neighboring gene, as many have a functional trans effect on other loci further away in the linearity of the DNA."
The presented analyses based on Fst and ROD are an insightful first step for identifying candidate genes that might be under selection, but it is not proved the causal variation improving fitness in adaptation is within all those genes or affecting those gene functions. Colocalization of 150 genes and selective sweep regions don't prove these are the genes involved in adaptation and such relationship can be explained by hitchhiking and recombination rates differences affecting linkage, for example.

Answer: Thanks for your comments. We agree with you that the analyses of selective sweep, Fst and ROD are the first step for identification of potential candidate genes [3]. We then mainly focused on those genes with non-synonymous SNPs. As previously reported, a non-synonymous or missence SNP may remarkably change the gene function. For example, in the Emily K. Don's study [4], three non-synonymous SNPs for amino acid substitutions (A78V and G79A) have led the function loss of tbx4 and further resulted in the pelvic finless of zebrafish. We thus identified the detailed distribution of those non-synonymous SNPs with significant P-value in the candidate genes to provide molecular guidance for in-depth studies on migratory adaptation. We also agree that these genes need further functional validation by using gene editing methods.
[3] Wang D, Zhang Y, Zhang Z, Zhu J, Yu J: KaKs_Calculator 2.0: a toolkit incorporating gamma-series methods and sliding window strategies. Genomics Proteomics Bioinformatics 2010, 8(1):77-80.
[4] Don EK, de Jong-Curtain TA, Doggett K, Hall TE, Heng B, Badrock AP, Winnick C, Nicholson GA, Guillemin GJ, Currie PD et al: Genetic basis of hindlimb loss in a naturally occurring vertebrate model. Biol Open 2016, 5(3):359-366.

Statements throughout the manuscript such as "In addition, population sequencing of 96 representative individuals from diverse areas along the putative migration path confirmed the involvement of 150 genes in migratory adaption." (abstract, line 37-40) are overstatements. The tone regarding that should be more like in the last sentence of the conclusion using the adjective candidate and/or putative, etc.(conclusion, line 271-274).

Answer: Thanks for your good advice. Yes, we changed the descriptive tone throughout the manuscript according to your instructions. Please find more details in the revised abstract (on lines 40-42) and conclusions (on lines 274-277).

The linkage map data is relevant by itself and you should consider publishing a data publication for further description, for example, to further look into for recombination rates (cM/Mb) and recombination hotspots relationship with the selective sweep and recombination role in adaptation.

Answer: Thanks for your good idea. We would like to have another publication in BMC Research Notes for a detailed description of the genetic linkage map.

Close
