# Supplementary material for: Genome and population sequencing of a chromosome-level genome assembly of the Chinese tapertail anchovy (Coilia nasus) provides novel insights into migratory adaptation
Source: Gigascience. 2020 Jan 2;9(1):giz157. doi: 10.1093/gigascience/giz157 (PMC6939831; doi:10.1093/gigascience/giz157)
Supplement: giz157_Reviewer_1_Report_Original_Submission — Ole K TÃ,rresen -- 7/1/2019 Reviewed [file giz157_reviewer_1_report_original_submission.pdf]

Reviewer Report

Title: Genome and population sequencing of a chromosome-level genome assembly of the Chinese tapertail anchovy (Coilia nasus) provides novel insights into migratory adaptation

Version: Original Submission **Date:** 7/1/2019

Reviewer name: Ole K TÃ¸rresen

Reviewer Comments to Author:

General comments
The authors have sequenced and assembled the Chinese tapertail anchovy (Coilia nasus). The Chinese tapertail anchovy is intriguing because it has both migratory and non-migratory populations, facilitating investigation into the genetic basis for migration.
By sequencing 96 individuals (85 migratory and 11 non-migratory), the authors find 150 genes that have vastly different alleles between the migratory and non-migratory ecotypes.
There are multiple instances where the language is unclear or wrong. I would strongly advice going through the text carefully to find these places, maybe by a native English-speaking person. I have mentioned some places, but I would like to focus on the science and not the language.
Specific comments
Title: I prefer "population sequencing" rather than "resequencing", but that is a personal preference and not a recommendation for a change.
Abstract: The 'Results' part is a bit heavy to read. You could spilt the first to sentences to get a better flow.
Line 60: "of" is not necessary.
Line 63: "molecular mechanisms have still been unknown" should be "molecular mechanisms are still unknown".
Line 69: Does the cited article actually discuss the fish' "nutritional content and delicate flavor"? Anyhow, I am uncertain of the validity of having this kind of statements in a scientific paper. People would not buy food that tastes bad, and over time, would not buy food without enough nutrition.
Line 76: I don't think the approximation symbol is used as this usually.
Line 76-77: Could be written better.
Line 78: "always resident" and "their whole lifetime" means the same.
Line 87: RAD actually used for creating the assembly? Rewrite, the important part is that you created a linkage map and used that, not the RAD itself.
Line 90: What does "high-evidence for examination of the detailed molecular" mean?
Line 92-93: Could be written better.
Line 104: You could specify that this was using the actinopterygii dataset.
Line 105: "A BUSCO evaluation of our assembly was 90.1%," does not make sense. Please rewrite.
Line 125-126: Is this normal? Seems a bit low.
Lines 128-130: You cannot call 25 million SNPs for 25 Mb SNPs. You could say 25 M SNPs however.

Line 134: The "SNP set" is all 39 M SNPs? Or is it a reduced set? How many SNPs in that case?
Line 136: Why so few non-migratory? Would it not be better comparing two groups of equal size than this large difference?
Lines 158-159: "suggesting that the migratory adaptation may require a preference for selected genes in adjacent locations". What does this mean? How can you tell if this is a real phenomenon?
Line 171: I do not think you can say that "genes were clustered in terms of [..] physical (chromosomal) positions" if your example in line 162 is typical. 3 genes spread across 5 Mbp is not a lot. Further, how can you say that the genes are "clustered [..] in [..] physiological functions"? Would not this be expected? A migratory behavior would need some triggers, which some of the GO pathways might be, and would need some neurological connection. I do not find this surprising. Is is as expected, I would say.
Section "Differentially expressed genes (DEGs) in the Ca2+-related pathways for the migrator group": If these genes are always more expressed in migratory fish than non-migratory fish, how can you tell if they are actually connected to migration? Would not the expectation be that something creates a cascade and up-regulates a bunch of genes, which then make the fish do the migration. If the genes are up-regulated the whole time, how would that contribution to the migration? When were the analysis done? I see from the section called "Genetic adaptation to complex environments during migration" that you say that these might be needed for the migratory fish to handle freshwater, which seems logical.
Line 303: Has the SOAPdenovo assembly been used for anything? That is not clear to me.
Line 314: Why did you generate a Platanus assembly if you had a SOAPdenovo assembly already?
Line 332: Which assembly was annotated?
Line 408: Why have you used both assemblies in your analysis? You should in that case report the statistics and BUSCO scores for the SOAPdenovo assembly in the results, so that we as readers can assess its quality better.
Section "Availability of supporting data": I could not find any of the genome assemblies on the two provided project IDs. Please make sure that they are released.
Figure 2: Why do you have that particular order of the chromosomes? Why not 1,2,3,4 etc? What is the naming of the chromosomes based on? It is common to call the largest sequence for chromosome 1, second largest for chromosome 2 and so on. This does not seem to be the case here.
Supporting data: Please provide the SOAPdenovo assembly here also.

Methods

Are the methods appropriate to the aims of the study, are they well described, and are necessary controls included? Choose an item.

Conclusions

Are the conclusions adequately supported by the data shown? Choose an item.

Reporting Standards

Does the manuscript adhere to the journal's guidelines on [minimum standards of reporting?]() Choose an item.

Choose an item.

Statistics

Are you able to assess all statistics in the manuscript, including the appropriateness of statistical tests used? Choose an item.

Quality of Written English

Please indicate the quality of language in the manuscript: Choose an item.

Declaration of Competing Interests

Please complete a declaration of competing interests, considering the following questions:

- Have you in the past five years received reimbursements, fees, funding, or salary from an organisation that may in any way gain or lose financially from the publication of this manuscript, either now or in the future?
- Do you hold any stocks or shares in an organisation that may in any way gain or lose financially from the publication of this manuscript, either now or in the future?
- Do you hold or are you currently applying for any patents relating to the content of the manuscript?
- Have you received reimbursements, fees, funding, or salary from an organization that holds or has applied for patents relating to the content of the manuscript?
- Do you have any other financial competing interests?
- Do you have any non-financial competing interests in relation to this paper?

If you can answer no to all of the above, write 'I declare that I have no competing interests' below. If your reply is yes to any, please give details below.

I declare that I have no competing interests

I agree to the open peer review policy of the journal. I understand that my name will be included on my report to the authors and, if the manuscript is accepted for publication, my named report including any attachments I upload will be posted on the website along with the authors' responses. I agree for my report to be made available under an Open Access Creative Commons CC-BY license (http://creativecommons.org/licenses/by/4.0/). I understand that any comments which I do not wish to be included in my named report can be included as confidential comments to the editors, which will not be published.

Choose an item.

To further support our reviewers, we have joined with Publons, where you can gain additional credit to further highlight your hard work (see: https://publons.com/journal/530/gigascience). On publication of this paper, your review will be automatically added to Publons, you can then choose whether or not to claim your Publons credit. I understand this statement.

Yes Choose an item.
