# Supplementary material for: Genome and population sequencing of a chromosome-level genome assembly of the Chinese tapertail anchovy (Coilia nasus) provides novel insights into migratory adaptation
Source: Gigascience. 2020 Jan 2;9(1):giz157. doi: 10.1093/gigascience/giz157 (PMC6939831; doi:10.1093/gigascience/giz157)
Supplement: giz157_Reviewer_2_Report_Original_Submission — Bruno Louro -- 7/8/2019 Reviewed [file giz157_reviewer_2_report_original_submission.pdf]

Reviewer Report

Title: Genome and population sequencing of a chromosome-level genome assembly of the Chinese tapertail anchovy (Coilia nasus) provides novel insights into migratory adaptation

Version: Original Submission **Date:** 7/8/2019

Reviewer name: Bruno Louro

Reviewer Comments to Author:

The article reports the chromosome-wide genome assembly of the Chinese tapertail anchovy, and its respective use for population genomics studies. The genomic tools and data generated are of high relevance, as demonstrated here by its usefulness to address a biological question, the evolutionary aspects of migratory dimorphism.
The major weakness of the manuscript is the overall description inaccuracy in the methods and results sections, as listed below:

* Results presented without any respective methodology description, for example synteny blocks in Figure 2, enrichment pathway analysis for 150 candidate genes, BUSCO assessment (guessed the actinopterygii dataset was used by the number of gene models), and DEGs Heatmap in Figure 5g.
* Regarding population structure the results presented are solely a phylogenetic tree figure without presenting consolidated statistical values. Additionally confirmed by otoliths microstructure results, but no value is presented (eg . correlation). In the respective methods part is mentioned "PLINK was applied to calculate the genetic distance between individuals,..". What was the actual analysis procedure done with PLINK?
* Results mixed in the methods section (eg. lines 280-282; line 286-287; 302; 315-316; 319-320; 326-327; 329-331; and 399-406).
* Resequencing methodology for the population genomic study mentioned pooled samples but supplementary file sequencing libraries are for each 96 individual samples.
* Methodologies in general are omissive regarding details necessary for reproducibility purpose or to understand the results reported, such as DNA amount used for libraries purposes, sampled tissues for qPCR, t-test assumption of data normality and equal variance, lack of the used software naming (line 306: "total reads were mapped onto the contigs") and/or respective parameters (if default was used state it).
* One example of methodology description not being accurate is the RNAseq expression analysis (line431-440). No description to the raw data editing and quality control filtering procedure. The mapping procedure solely names the HISAT2 without any parameters used specified. The usage of Cufflinks 2.2.1 suite of tools description is confusing; solely mentioning the Cufflinks program itself (which assembles the mapped reads into transcriptomes) and Cuffdiff to identify DEGs. No parameters of the programs are described, and no reference to the intermediate softwares in the workflow is done, such as Cuffcompare, Cuffmerge, CuffQuant and downstream the Cuffnorm. Presuming the usage of Cuffnorm to normalize the expression values across samples required for the heatmap presented in Figure 5g.

* I'm not against the use of personal pronouns in scientific manuscript writing, but the extensive use of "We" in the results and in methods sections should be avoided, because doing so removes objectivity required in these sections.
Without having addressed these main points listed above, is difficult to evaluate the fullness and implications of the results presented. Therefore, any other revision queries are not relevant until methods & results sections are revised. As exception, just to raise one point regarding one particular analysis and respective conclusion. This is about the identification of the 150 candidate genes related to the migratory adaptation, solely based on the colocalization with selective sweep regions. These genomic signatures of divergence don't necessarily have a cis effect on the neighbouring gene, as many have a functional trans effect on other loci further away in the linearity of the DNA. If you wanted to focus on the functional gene unit, why didn't you complemented it with the Non-synonymous/Synonymous mutation ratio (Ka/Ks) analysis of those genes, since you identified those coding region located SNPs?
To summarise it, the genomic tools, the data generated and the biological question addressed are of high relevance, but hampered by the manuscript methods and results reporting.

Methods

Are the methods appropriate to the aims of the study, are they well described, and are necessary controls included? Choose an item.

Conclusions

Are the conclusions adequately supported by the data shown? Choose an item.

Reporting Standards

Does the manuscript adhere to the journal's guidelines on minimum standards of reporting? Choose an item.

Choose an item.

Statistics

Are you able to assess all statistics in the manuscript, including the appropriateness of statistical tests used? Choose an item.

Quality of Written English

Please indicate the quality of language in the manuscript: Choose an item.

Declaration of Competing Interests

Please complete a declaration of competing interests, considering the following questions:

- Have you in the past five years received reimbursements, fees, funding, or salary from an organisation that may in any way gain or lose financially from the publication of this manuscript, either now or in the future?
- Do you hold any stocks or shares in an organisation that may in any way gain or lose financially from the publication of this manuscript, either now or in the future?
- Do you hold or are you currently applying for any patents relating to the content of the manuscript?
- Have you received reimbursements, fees, funding, or salary from an organization that holds or has applied for patents relating to the content of the manuscript?
- Do you have any other financial competing interests?
- Do you have any non-financial competing interests in relation to this paper?

If you can answer no to all of the above, write 'I declare that I have no competing interests' below. If your reply is yes to any, please give details below.

I declare that I have no competing interests.

I agree to the open peer review policy of the journal. I understand that my name will be included on my report to the authors and, if the manuscript is accepted for publication, my named report including any attachments I upload will be posted on the website along with the authors' responses. I agree for my report to be made available under an Open Access Creative Commons CC-BY license (http://creativecommons.org/licenses/by/4.0/). I understand that any comments which I do not wish to be included in my named report can be included as confidential comments to the editors, which will not be published.

Choose an item.

To further support our reviewers, we have joined with Publons, where you can gain additional credit to further highlight your hard work (see: https://publons.com/journal/530/gigascience). On publication of this paper, your review will be automatically added to Publons, you can then choose whether or not to claim your Publons credit. I understand this statement.

Yes Choose an item.
