# Supplementary material for: Genome and population sequencing of a chromosome-level genome assembly of the Chinese tapertail anchovy (Coilia nasus) provides novel insights into migratory adaptation
Source: Gigascience. 2020 Jan 2;9(1):giz157. doi: 10.1093/gigascience/giz157 (PMC6939831; doi:10.1093/gigascience/giz157)
Supplement: giz157_Reviewer_2_Report_Revision_1 — Bruno Louro -- 11/2/2019 Reviewed [file giz157_reviewer_2_report_revision_1.pdf]

Reviewer Report

Title: Genome and population sequencing of a chromosome-level genome assembly of the Chinese tapertail anchovy (Coilia nasus) provides novel insights into migratory adaptation

Version: Revision 1 **Date:** 11/2/2019

Reviewer name: Bruno Louro

Reviewer Comments to Author:

The main concern about methods and results reporting format in the previous revision was properly addressed and most doubts properly answered. The minor details that now require attention are listed below:
The results subsection "Sequencing and assembly of the chromosome-level genome" should be named "Sequencing, assembly, and annotation of the chromosome-level genome"
The supplementary Material file is called "Overall opinion.docx".
Line 98-100: Still not clear which assembly you are describing.
Line 180-182: In "Supplementary Table 13. Summary of the 277-non-synonymous SNPs in the CDS regions of the 14 Ca2+-related genes (see the separate excel file)", only 45 SNPs are described and no significant statistical value is presented.
Line 184: (Figure 5a-f)
Line 182 -189: No statistical values/significance presented in text or figure 5.
Figure 5g: Gene name instead of your internal gene ID is more informative.
Supplementary Table 14. Describe the expression values presented.
Line 285: Missing kit and sequence platform name/references.
Line 362-363: Don't understand this sentence: "The sorted and integrated 363 steps were performed in these alignments."
Line 388: "SNP calling." Is it a subsection title?
Line 445-448: Which software was used for quality control and editing of the raw reads?
Line 452-457: Cuffdiff was used for DEG analysis and EdgeR to "draw the heatmap"? Do you mean you used EdgeR to normalize expression across samples required to draw the heatmap? If you used Cuffdiff why didn't you use Cuffnorm/CummRbund to output the heatmaps?
The main question which was not properly addressed from the previous revision: "about the identification of the 150 candidate genes related to the migratory adaptation, solely based on the colocalization with selective sweep regions. These genomic signatures of divergence don't necessarily have a cis effect on the neighboring gene, as many have a functional trans effect on other loci further away in the linearity of the DNA."
The presented analyses based on Fst and ROD are an insightful first step for identifying candidate genes that might be under selection, but it is not proved the causal variation improving fitness in adaptation is within all those genes or affecting those gene functions. Colocalization of 150 genes and selective sweep regions don't prove these are the genes involved in adaptation and such relationship can be explained by hitchhiking and recombination rates differences affecting linkage, for example.

Statements throughout the manuscript such as "In addition, population sequencing of 96 representative individuals from diverse areas along the putative migration path confirmed the involvement of 150 genes in migratory adaption." (abstract, line 37-40) are overstatements. The tone regarding that should be more like in the last sentence of the conclusion using the adjective candidate and/or putative, etc.(conclusion, line 271-274).
The linkage map data is relevant by itself and you should consider publishing a data publication for further description, for example, to further look into for recombination rates (cM/Mb) and recombination hotspots relationship with the selective sweep and recombination role in adaptation.

Methods

Are the methods appropriate to the aims of the study, are they well described, and are necessary controls included? Choose an item.

Conclusions

Are the conclusions adequately supported by the data shown? Choose an item.

Reporting Standards

Does the manuscript adhere to the journal's guidelines on minimum standards of reporting? Choose an item.

Choose an item.

Statistics

Are you able to assess all statistics in the manuscript, including the appropriateness of statistical tests used? Choose an item.

Quality of Written English

Please indicate the quality of language in the manuscript: Choose an item.

Declaration of Competing Interests

Please complete a declaration of competing interests, considering the following questions:

- Have you in the past five years received reimbursements, fees, funding, or salary from an organisation that may in any way gain or lose financially from the publication of this manuscript, either now or in the future?
- Do you hold any stocks or shares in an organisation that may in any way gain or lose financially from the publication of this manuscript, either now or in the future?

- Do you hold or are you currently applying for any patents relating to the content of the manuscript?
- Have you received reimbursements, fees, funding, or salary from an organization that holds or has applied for patents relating to the content of the manuscript?
- Do you have any other financial competing interests?
- Do you have any non-financial competing interests in relation to this paper?

If you can answer no to all of the above, write 'I declare that I have no competing interests' below. If your reply is yes to any, please give details below.

I declare that I have no competing interests.

I agree to the open peer review policy of the journal. I understand that my name will be included on my report to the authors and, if the manuscript is accepted for publication, my named report including any attachments I upload will be posted on the website along with the authors' responses. I agree for my report to be made available under an Open Access Creative Commons CC-BY license (http://creativecommons.org/licenses/by/4.0/). I understand that any comments which I do not wish to be included in my named report can be included as confidential comments to the editors, which will not be published.

Choose an item.

To further support our reviewers, we have joined with Publons, where you can gain additional credit to further highlight your hard work (see: https://publons.com/journal/530/gigascience). On publication of this paper, your review will be automatically added to Publons, you can then choose whether or not to claim your Publons credit. I understand this statement.

Yes Choose an item.
